# Structure of core assembly of the *Clostridioides difficile* germinosome

**DOI:** 10.1038/s41467-026-74264-w

**Published:** 2026-06-17

**Authors:** Carlos Cal y Mayor Luna, Martín Alcorlo, Choon Kim, Giselle N. Jacobson, Melisa Lázaro, Mikel Valle, Mayland Chang, Juan A. Hermoso, Shahriar Mobashery

**Affiliations:** 1 Department of Chemistry and Biochemistry, University of Notre Dame, Notre Dame, IN USA; 2 Department of Crystallography and Structural Biology, Instituto de Química-Física “Blas Cabrera”, Consejo Superior de Investigaciones Científicas, Madrid, Spain; 3 Biophysics Instrumentation Core Facility, University of Notre Dame, Notre Dame, IN USA; 4 Center for Cooperative Research in Biosciences (CIC bioGUNE), Basque Research and Technology Alliance (BRTA), Bizkaia Technology Park, Derio, Spain

**Keywords:** Proteins, Cryoelectron microscopy, X-ray crystallography, Bacteriology

## Abstract

The germinosome is the machinery of *Clostridioides difficile* that sets in motion the process of spore germination to vegetative bacteria. Three highly-regulated proteins—CspA, CspB and CspC—serve as key instigators of germination. We report that CspA and CspB exist independently as homodimers in solution. In the presence of CspC, a picomolar complex of CspA:CspC forms. Furthermore, we document that CspA binds to the germinant, taurocholate, and that the complex CspA:CspC:taurocholate serves as the receptor for glycine, the co-germinant. We report high-resolution X-ray and cryo-EM structures for the three proteins, and for the CspA:CspC:taurocholate complex. These structures show how the CspA:CspC heterodimer recognizes taurocholate and reveal that specific structural features in the three Csp proteins avoid the recognition of the germinant by homodimers. Remarkably, the homodimer of CspB organizes itself in a supramolecular fibril assembly comprised of a three-stranded right-handed superhelix. These proteins are targets for interference with the transition from spore to the vegetative form of *C. difficile*.

## Abstract (web summary)

*Clostridioides difficile* spore germination is triggered by the germinosome protein machinery. Here the authors reveal the nature of the interactions and structures of Csp germinosome proteins underlying the process.

## Introduction

**Fig. 1:**
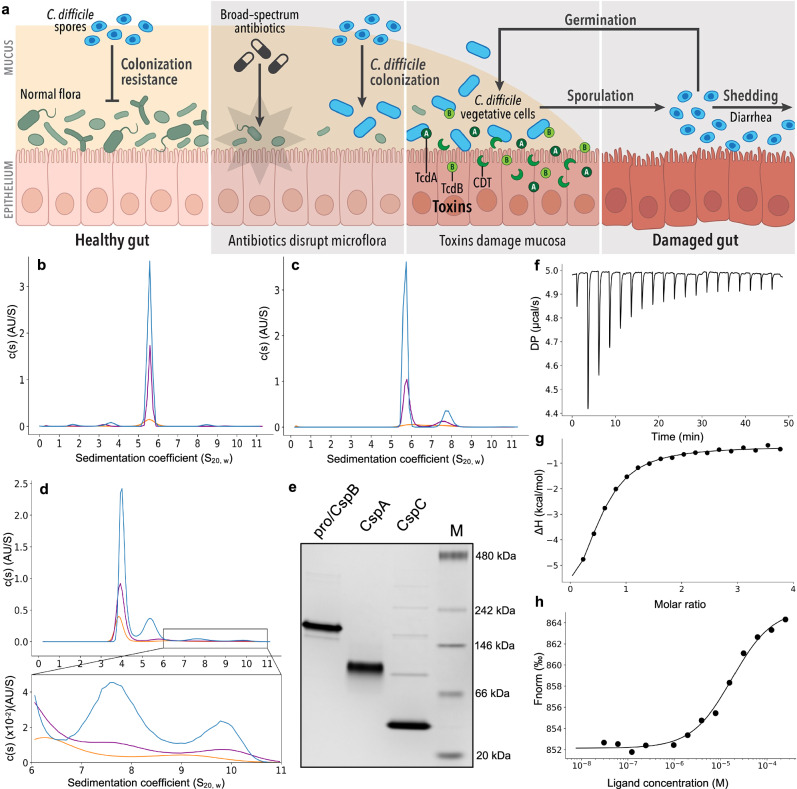
Overview of the *C. difficile* life cycle and oligomeric state and binding properties of the Csp proteins. **a** A schematic of the life cycle of *C. difficile* is given. Once spores germinate, *C. difficile* vegetative cells produce toxins TcdA, TcdB, and CDT, which affect the epithelial barrier function, leading to gut damage, inflammation, and diarrhea. In response to nutrient deprivation, *C. difficile* vegetative cells sporulate, and spores are shed. **b** Sedimentation velocity (SV) analysis of CspA at 1.3 µM (orange), 4.7 µM (purple) and 12.6 µM (blue) displayed a single peak at 5.5 S_20,w_ corresponding to a dimer, **c** SV analysis of pro/CspB at 1.5 µM (orange), 6.5 µM (purple) and 17.8 µM (blue) showed a predominant peak at 5.7 S_20,w_ and a minor peak at 7.8 S_20,w_, corresponding to a dimer and a trimer, respectively. **d** SV analysis of CspC at 1.9 µM (orange), 5.5 µM (purple), and 13.0 µM (blue) exhibited a major peak at 3.9 S_20,w_, consistent with a monomer. At 13 µM, CspC peaks were observed at 5.2, 7.6, and 9.8 S_20,w_ corresponding to dimeric, trimeric, and tetrameric species, respectively. The expanded view below (**d**) highlights the trimeric and tetrameric CspC. **e** Representative native-PAGE of pro/CspB, dimer; CspA, dimer; CspC, various oligomers (at ~12 µM). M is the molecular-weight marker. Experiment performed in triplicate. **f** Representative ITC thermogram showing the interaction of CspA and taurocholate. **g** Binding isotherm of the interaction of CspA and taurocholate. **h** A representative dose-response curve of MST is given for the interaction between CspA and taurocholate.

*Clostridioides difficile* is an anaerobic, Gram-positive, spore-forming bacterium classified as an urgent threat to human health by the U.S. Centers for Disease Control and Prevention^1^. This pathogen was responsible for an estimated 202,600 hospitalizations and 11,500 deaths in 2019 in the US alone^2^, with attributable healthcare costs reaching $1.8 billion in 2017^1^. *C. difficile* infections (CDIs) result in moderate diarrhea to life-threatening pseudomembranous colitis^3^. The complication is associated with microbiota dysbiosis, often linked to the use of broad-spectrum antibiotics for a different infection^4^. *C. difficile* produces the toxins TcdA, TcdB, and the binary toxin CDT. TcdA and TcdB are enzymes that glycosylate members of the Rho family of GTPases. The enzymatic reactions trap Rho in an inactive conformation, which disrupts actin cytoskeleton regulation and epithelial barrier function, leading to gut damage, inflammation, and diarrhea^5^ (Fig. 1a).

Vegetative *C. difficile* transforms into spores in response to stress during sporulation. Germination is the reversal of this process, facilitated by an assembly of proteins referred to as the germinosome. *C. difficile* spores survive in harsh and aerobic conditions^6,7^. Spores contain a partially dehydrated core, which holds up to 1 M calcium dipicolinate, a feature that plays a pivotal role in conferring heat resistance^8^. The core is surrounded by a protective inner phospholipid bilayer^7^, followed by a germ cell wall that eventually transforms to the vegetative cell wall^9^. Next is the cortex, consisting of a “thick” layer of modified peptidoglycan, composed of polymers of repeating disaccharide *N*-acetylglucosamine (NAG)-*N*-acetylmuramate (NAM)^10^. The NAM unit has a distinct bacterial stem peptide linked to it. The stem peptides are removed by the complex of CwlD and GerS during sporulation, followed by deacetylation of the NAM unit and the formation of a ${{{\rm{\delta }}}}$-lactam ring by the protein PdaA1^11–13^. This modification of the linked peptide was believed to be important to ensure that the lytic transglycosylase SleC degrades only the cortex and not the germ cell wall; however, this requirement does not seem to be strict^14^. Outside the cortex is an outer phospholipid bilayer, which is further surrounded by a spore-coat region. Finally, the outermost layer is the exosporium, a highly permeable carbohydrate-rich layer, likely involved in host-spore interactions^15^.

The process of germination involves a cascade of highly regulated events that are initiated by the germinant (taurocholate) and co-germinant (amino acids such as glycine). The onset of germination is mediated by three key proteins with subtilisin-like sequences—*Clostridium* serine proteases (Csp)—which are the subjects of the present report. CspA and CspC harbor amino-acid changes that render them as inactive pseudo-proteases, whereas CspB is a bona fide protease. The Csp proteins are encoded by a bicistronic gene *cspBA-cspC*^16^. CspC is translated as a single polypeptide, whereas CspA and the zymogenic proCspB are produced as a fused protein (proCspBA) that subsequently undergoes processing by the protease YabG during sporulation^17^. Meanwhile, as proCspB is generated by the reaction of YabG, it undergoes autoproteolysis to produce the mature form, CspB. Mature CspB hydrolytically cleaves the prodomain of proSleC, which degrades the spore cortex to set in motion the germination process^17–20^.

In the present report, we investigate the interactions among the three Csp proteins as the core of the germinosome, and the influences of the germinant and co-germinant. We report herein that CspA and CspC form a stable heterodimer, and that the generated CspB processes proSleC to set in motion degradation of the cortex peptidoglycan. We document that the complex of CspA:CspC serves as the receptor for the germinant taurocholate. None of the individual Csp proteins, nor the CspA:CspC complex, binds the co-germinant glycine. Instead, the CspA:CspC:taurocholate complex serves as the glycine receptor. These studies are further complemented by a set of X-ray and cryo-electron microscopy (cryo-EM) structures for all three CspA, CspB and CspC, including the CspA:CspC complex bound to taurocholate, which provides the molecular basis for the interactions among the proteins and the germinant. Collectively, the work sheds light on the core of the germinosome as a key catalytic machinery for the process of spore germination.

## Results

### Oligomeric states of the Csp proteins in solution

We produced the recombinant Csp proteins to apparent homogeneity (Supplementary Fig. 1a) and studied them with a variety of biophysical techniques (Supplementary Methods). Sedimentation velocity analytical ultracentrifugation (SV-AUC) was used to assess the oligomeric states of the individual Csp proteins. CspA exists exclusively as a dimer, at concentrations of 1.3–12.6 µM, with a sedimentation coefficient of 5.5 S_20,w_ (Fig. 1b) and an estimated molar mass of 110 kDa (Supplementary Fig. 1c), consistent with a dimer. Our efforts to produce mature CspB failed because of solubility issues (described in Supplementary Methods), hence the experiments were performed with pro/CspB [a construct produced by co-expressing the genes for the prodomain and CspB (Supplementary Methods)]. The majority of pro/CspB exists as a dimer with a sedimentation coefficient of 5.7 S_20,w_ and estimated molar mass of 97 kDa at concentrations of 1.5–17.8 µM (Fig. 1c and Supplementary Fig. 1d). A minor species of 7.8 S_20,w_ and estimated molar mass of 154 kDa, corresponding to the trimer, was also observed. Since CspA or pro/CspB monomers were not observed above 1 µM, we repeated the SV-AUC experiments with monitoring at 230 nm to investigate the Csp oligomeric state at submicromolar concentrations. However, even at 200 nM CspA or 180 nM pro/CspB (limit of the experiment), no monomeric protein was detected (Supplementary Fig. 1e, f). The absence of monomeric protein in this analysis indicates the monomer–dimer *K*_d_ is <200 nM for both CspA and pro/CspB. In contrast, CspC showed a more heterogeneous oligomeric distribution (Fig. 1d). Most CspC consists of a species of 3.9 S_20,w_, corresponding to the monomer. At 13 µM, a second species of 5.2 S_20,w_ representing the dimer, was observed. Small amounts of trimeric (7.6 S_20,w_) and tetrameric (9.8 S_20,w_) CspC were also observed (inset of Fig. 1d). This more complex oligomeric profile led to an underestimation of the molecular mass (Supplementary Fig. 1g), likely due to the limitations in the mathematical analysis of the polydispersed mixtures. The oligomeric states of CspA, pro/CspB, and CspC were further supported by native-polyacrylamide gel electrophoresis (native-PAGE) (Fig. 1e). At 12 µM, both CspA, and pro/CspB exist mainly as dimers, and CspC as various oligomeric species, but primarily as a monomer.

### Investigations of interactions of the Csp proteins with sodium taurocholate or glycine

Taurocholate (a bile salt) and glycine have been reported as germinant and co-germinant, respectively^20–22^. Both have been implicated in the activation of CspB. We assessed these interactions by isothermal-titration calorimetry (ITC) and microscale thermophoresis (MST) using purified proteins. We found that CspA interacts with taurocholate. ITC measurements revealed a dissociation constant (*K*_d_) of 9 ± 3 µM for taurocholate, when dimeric CspA (50 µM) was used (Fig. 1f, g). Analysis at lower concentrations could not be performed because the level of detection was smaller under those conditions. In systems with larger *K*_d_ values, a non-sigmoidal isotherm results, which preclude reliable determination of the binding stoichiometry^23^. The binding is enthalpically driven and displayed an average Δ*H* = –6.7 ± 0.9 kcal/mol, Δ*G* = –6.9 ± 0.2 kcal/mol, and –TΔ*S* = 0 ± 1 kcal/mol. MST experiments with the Cy5-labeled CspA (5 nM) revealed a *K*_d_ of 17 ± 2 μM (Fig. 1h). In contrast, no binding of taurocholate was detected (by ITC or MST) either to pro/CspB or to CspC. Importantly, ITC and MST documented the absence of binding by glycine to any of the Csp proteins individually.

### Pairwise interactions of the Csp proteins

**Fig. 2:**
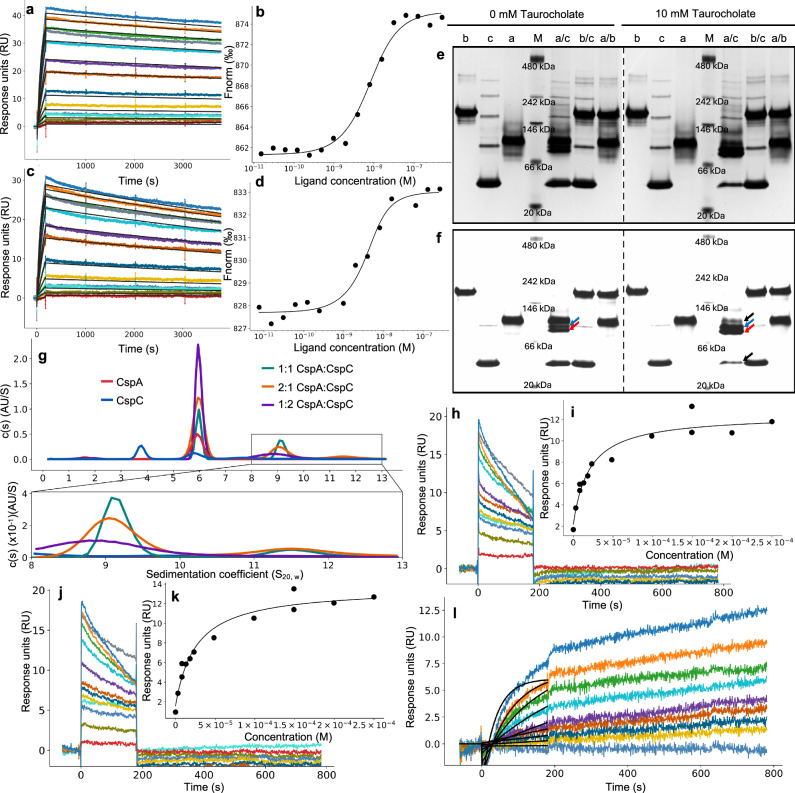
Binding analysis of CspA and CspC using SPR, MST, and Native-PAGE. **a**, **b** Representative SPR sensorgrams showed interaction between ST-CspC and CspA in the absence (**a**) and the presence (**c**) of 1 mM sodium taurocholate. The data fit to a one-to-one binding model (highlighted by overlayed black traces), gave *K*_d_ = 97 ± 8 pM (**a**) and 190 ± 2 pM (**c**)(χ^2^ < 3% of R_max_). **b**, **d** Representative MST dose-response curves are given for Cy5-labeled CspA and CspC interaction without (**b**) or with (**d**) 0.25 mM sodium taurocholate. The data fit gave *K*_d_ = 4.8 ± 0.4 nM (**b**) and 1.4 ± 0.2 nM (**d**). **e**, **f** Representative native-PAGE of individual proteins and mixtures of CspA, CspC, and pro/CspB with or without 10 mM taurocholate: b, pro/CspB; c, CspC; a, CspA; various mixture permutations of the two proteins. M is the molecular-weight markers. Blue arrows indicate CspC homodimer; red arrows indicate a new band from CspA:CspC heterocomplex (increased in the presence of taurocholate); black arrows indicate a decrease of CspA dimer and CspC monomer. Experiment performed in triplicate. **f** High-contrast image of (**e**). The dashed line indicates the grouped images were from two different gels. **g** SV-AUC at 230 nm of CspA (635 nM, red), CspC (635 nM, blue) and mixtures at 1:1 (625 nM each), 2:1 (1250 nM CspA, 625 nM CspC), and 1:2 (625 nM CspA, 1250 nM CspC). The mixtures show a predominant species corresponding to the dimer (6 S_20,w_) with two minor species corresponding to the tetramer (9.1 S_20,w_) and hexamer (11.4 S_20,w_) (expanded view **g**). **h**, **j** Representative SPR sensorgrams for (**h**) ST-CspC:CspA and taurocholate and (**j**) CspA-ST alone with taurocholate are given. **i**, **k** Steady-state affinity fits (solid line) for **i** ST-CspC:CspA and taurocholate (apparent *K*_d_ = 18.6 ± 0.4 µM) and **k** CspA-ST and taurocholate (apparent *K*_d_ = 26 ± 2 µM). **l** A representative SPR sensorgram of the interaction of ST-CspC:CspA:taurocholate complex with glycine is given. The data fit to a two-state reaction model gave an overall *K*_d_ of 11 ± 6 nM (χ^2^ < 7% of R_max_).

The pairwise interactions among the Csp proteins were investigated using surface-plasmon resonance (SPR) and MST. Due to the low isoelectric points (pI) of the Csp proteins (close to 4.5), direct immobilization of these proteins onto the SPR chips was not possible. To circumvent this problem, we used a Strep-Tactin XT capturing method. The constructs of full-length CspB and CspC with *N*-terminal Twin-Strep-tag (hereafter referred to as ST-proCspB and ST-CspC) were produced. For the case of CspA, the Twin-Strep-tag was introduced to the *C*-terminus (CspA-ST), as the *N*-terminal Twin-Strep-tagged CspA (ST-CspA) was not captured on the Strep-Tactin XT chip due to proteolysis of ST during production (Supplementary Fig. 1a). ST-CspC was captured on the Strep-Tactin XT chip, and its interaction with CspA or pro/CspB was assessed. The values for *k*_on_, *k*_off_, and *K*_d_ evaluated for ST-CspC and CspA were 4.1 ± 0.2 × 10^5 ^M^−1^s^−1^, 3.9 ± 0.2 × 10^−5 ^s^−1^ and 97 ± 8 pM, respectively (Fig. 2a). Furthermore, MST analyses in solution (immobilization-free, but requiring fluorescent tagging) using Cy5-labeled CspA confirmed the interaction with CspC. However, the dissociation constant was somewhat higher by this method (*K*_d_ of 4.8 ± 0.4 nM) (Fig. 2b), likely due to the incorporation of the fluorescent label near the binding site. Notably, when CspA-ST was captured on the Strep-Tactin XT chip, no interaction with CspC was observed, implying that immobilization likely occluded the binding site on CspA. This suspicion was borne out by the CspA:CspC structure (discussed later). Similarly, when CspC was labeled with Cy5 dye, it failed to interact with CspA, again, likely due to the occlusion of the binding site by the dye.

We next explored the interactions of pro/CspB with CspA and CspC. pro/CspB showed poor interaction with CspA or CspC by MST and SPR (several permutations were tested): CspA-ST and pro/CspB, ST-proCspB and CspA, ST-proCspB and CspC, ST-CspC and pro/CspB, Cy5-CspA and pro/CspB, Cy5-pro/CspB and CspA, Cy5-pro/CspB and CspC, Cy5-CspC and pro/CspB). The analyses had to be carried out at high concentrations (> 50 µM) and did not show saturation. These observations are likely due to non-specific protein-protein interactions. We infer that pro/CspB does not interact with CspA and CspC.

To evaluate the effect of taurocholate on the CspA:CspC interaction, CspA was incubated with taurocholate (0.25 mM for MST and 1 mM for SPR). The MST and SPR running buffers were also supplemented with 0.25 mM and 1 mM taurocholate, respectively, to maintain consistent ligand saturation during measurement. In both cases, the concentration of taurocholate was more than 25-fold above the *K*_d_ for the complex. In the presence of taurocholate, the values for *k*_on_, *k*_off_, and *K*_d_ for the interaction of CspA and ST-CspC were 4.6 ± 0.4 × 10^5 ^M^−1^s^−1^, 8.5 ± 0.4 ×  10^−5 ^s^−1^, and 190 ± 2 pM, respectively, by SPR (Fig. 2c); a twofold difference in *K*_d_. The *K*_d_ for the interaction of Cy5-CspA with CspC was a mere 3.4-fold lower (1.4 ± 0.2 nM) by MST (Fig. 2d). The presence of taurocholate did not influence the interactions between CspA and ST-CspC or between Cy5-CspA and CspC appreciably at the low concentrations used for SPR and MST.

In addition, the interaction between CspA and CspC was confirmed by native-PAGE (Fig. 2e, f). This experiment also showed the formation of the heterocomplex of CspA:CspC (the red arrows in Fig. 2f) in the absence of taurocholate. The formation of CspA:CspC heterocomplex increases in a concentration-dependent manner, for which we used 0, 0.1, 0.5, 2, and 10 mM taurocholate. The maximal effect was seen at 10 mM, but the effect is seen at lower concentrations too, of which the critical micelle concentration is between 3 mM and 13 mM^24–26^. Moreover, taurocholate induced a decrease in dimeric CspA. The resultant increase in monomeric CspA leads to binding to monomeric CspC, to form the CspA:CspC complex. This may suggest that taurocholate enhances the formation of the CspA:CspC complex by inducing the dissociation of the dimeric CspA, which agrees with the results of the structure of CspA:taurocholate (discussed below). We were not able to evaluate the effect of taurocholate on the CspA:CspC complex by MST, as the Cy5 dye interacts with taurocholate above 1 mM concentrations^27,28^. The negative results were the same by SPR, as a higher concentration of taurocholate induced non-specific binding on the SPR chips. Finally, SV-AUC was used to characterize complex formation between CspA and CspC. As CspA and CspC interact strongly (97 ± 8 pM by SPR and 4.8 ± 0.4 nM by MST), complex formation was monitored at 230 nm, enabling the usage of lower protein concentrations. Individual runs of CspA and CspC agreed with previous SV-AUC experiments monitored at 280 nm (Fig. 1b–d). CspA alone sediments as a dimer of 5.9 S_20,w_ (Fig. 2g). CspC alone sediments as four species: 41% of CspC existed as a monomer of 3.7 S_20,w_, 33% as dimer of 5.9 S_20,w_, 16% as trimer of 7.8 S_20,w_ and 9% as tetramer of 10.1 S_20,w_ (Fig. 2g). CspA and CspC were mixed at different ratios (1:1, 1:2 and 2:1) and the resulting sedimentation distribution revealed three peaks of 6, 9.1 and 11.4 S_20,w_ (Fig. 2g). Neither the 9.1 S_20,w_, nor 11.4 S_20,w_ peak were observed in CspA or CspC alone, indicating they represent CspA:CspC complexes. Interestingly, no monomeric CspC (3.7 S_20,w_) was present in the mixture, suggesting all monomeric CspC formed a complex with CspA. The 6 S_20,w_ peak had an estimated molecular mass of ~110 kDa, corresponding to the dimer, though it is not possible to distinguish between a homodimer or a heterodimer, as they are not hydrodynamically distinguishable. The peak at 9.1 S_20,w_ corresponds to a tetramer with a mass of ~200 kDa, and the peak at 11.4 S_20,w_ corresponds to a hexamer with an apparent mass of ~300 kDa. The peak at 6 S_20,w_ indicates that the complex predominantly exists as a dimer, but the CspA:CspC complex is also able to form minor tetrameric and hexameric species (expanded view in Fig. 2g).

### Investigations of the interaction of the heterocomplex CspA:CspC with sodium taurocholate and glycine

We next quantified the interactions of the heterocomplex with sodium taurocholate and glycine using SPR. As CspC does not bind taurocholate, and unbound CspA (to CspC) is washed away during the capturing step, this setup ensures that the measured interaction reflects only taurocholate binding to the heterocomplex. Furthermore, as a control, we determined that sodium taurocholate does not bind to the Strep-Tactin XT chip. Steady-state affinity fitting of the sensorgrams yielded an apparent dissociation constant of 18.6 ± 0.4 µM between the heterodimer ST-CspC:CspA and taurocholate (Fig. 2h, i). The kinetic parameters (*k*_on_ and *k*_off_) were outside the detection range of the instrument. We also measured the binding between CspA-ST and sodium taurocholate with SPR using a steady-state affinity fitting, which gave an apparent *K*_d_ of 26 ± 2 µM (Fig. 2j, k). The SPR data is consistent with ITC and MST data, yielding a dissociation constant in the micromolar range. According to the measurements, we do not see much of a difference in the dissociation constants between the homodimer of CspA-ST and the heterodimer ST-CspC:CspA with sodium taurocholate. Importantly, SPR also documented the absence of binding between the heterodimer and glycine, consistent with the lack of binding observed for the individual proteins and the co-germinant, as assessed earlier by ITC and MST. In the light of the suggestion that taurocholate binding may influence glycine recognition^29^, we used the same SPR experimental design to test for cooperative glycine binding to the CspA-ST:taurocholate and to the ST-CspC:CspA:taurocholate complexes. The proteins were incubated with 1 mM sodium taurocholate, and the SPR running buffer was supplemented with the same concentration (over 38-fold higher than the *K*_d_ of the complexes). SPR showed no detectable binding of glycine to the CspA-ST:taurocholate complex. In contrast, binding was detected with the ST-CspC:CspA:taurocholate complex (Fig. 2l). The data were fit to a two-state binding model, in which initial binding of the analyte (glycine in our case) to the capture ligand is followed by a second process that stabilizes the complex on a slower timescale than the first interaction. The first binding event was characterized by a *k*_on1_ of 2.46 ± 0.09 × 10^2 ^M^1^s^−1^ a *k*_off1_ of 2.4 ± 0.3 × 10^−^^5 ^s^−1^, producing a *K*_d_ of 101 ± 9 nM for glycine for the first state. The first event has a slow association phase; however, once the state forms, the onset of the second state ensues. The *k*_on2_ and *k*_off2_ for the second state were evaluated at 2.4 ± 0.8 × 10^−2^ s^−1^ and 4 ± 2 × 10^−^^3^ s^−1^, respectively. The transition to the second state takes place because *k*_on2_ is 1000-fold more rapid than *k*_off1_. The full two-state binding event results in a *K*_d-overall_ of 11 ± 6 nM for glycine. An inherent slight drift was observed in the raw data for the second state, as explained below.

The challenge in this experiment is that the sensorgrams reflect the interactions of a five-component assembly (CspA, CspC, and three molecules of taurocholate) with a ligand that is roughly 1600-fold smaller in molecular weight than its receptor. The detection requires high instrumental sensitivity. Furthermore, this non-covalent assembly needs to remain intact during measurements to favor binding by the sixth component, glycine. This challenge contributes to the aforementioned drift in the raw kinetics data. What is seen in the sensorgrams is that both the association and dissociation phases for glycine are slow processes. This is likely a slow-binding effect, involving a conformational change, which is rate-limiting for the formation of the final six-component system. This contributes to a baseline drift on the SPR chip. Importantly, this drift is small relative to the total response and does not affect the quality of the fit. The sensorgrams show concentration-dependent increases in response units. Global fitting to a two-state model yielded χ^2^ values below 7% of R_max_, indicating that the model accurately captures the observed kinetics. Together, the concentration-dependent binding, physically reasonable R_max_ value, and low χ^2^ value support the robustness of the data and validate the two-state binding model for glycine interaction with the CspC:CspA:3taurocholate complex.

### Three-dimensional structure of CspB

**Fig. 3:**
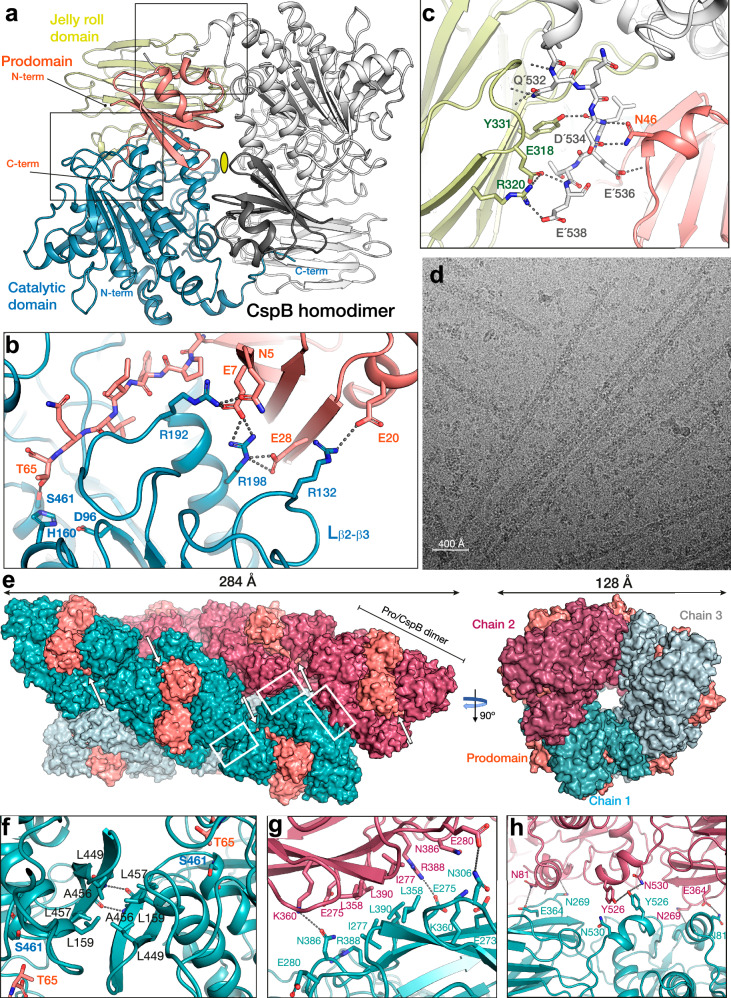
Three-dimensional structure of pro/CspB complex. **a** Crystal structure of the pro/CspB homodimer. In one monomer, the catalytic domain is colored blue, the jelly-roll domain green, and the prodomain coral, whereas in the other monomer, CspB is shown in light gray and the prodomain in dark gray. The pseudo-binary axis defining the dimer is represented as a yellow ellipsoid. **b** Zoomed-in view of the active site of CspB is shown with the catalytic triad represented as capped sticks. The *C*-terminal region of the prodomain inserted into the active site is represented as capped sticks. Main interacting residues between the prodomain and the catalytic domain are represented as capped sticks. Interactions are represented as dotted lines. **c** Close-up view of the interface between the jelly-roll domain (green) and prodomain (coral) of one monomer and the *C*-terminal end of the catalytic domain of its dimeric partner (white) is shown. Polar interactions are represented as dotted lines, and main residues are represented as capped sticks. **d** Cryo-EM image of the pro/CspB fibrils is shown. Experiment done in duplicate. **e** Cryo-EM reconstruction of a 284 Å-long segment of the pro/CspB fibril is depicted. The fibril is composed of three chains of pro/CspB dimers, with the prodomains highlighted in coral. The protease active sites distributed along the fibril are indicated by white arrows. Boxed regions highlight the interfaces contributing to the fibril assembly; from left to right: the intra-chain interface, inter-chain interface 1 and inter-chain interface 2. **f** Close-up view is provided of the intra-chain interface with key residues represented as capped sticks. The catalytic S461 and the *C*-terminal end of the prodomain (T65) are also depicted as capped sticks. **g**, **h** Zoomed-in views are shown of inter-chain interface 1 (**g**) and inter-chain interface 2 (**h**), respectively, with the principal interacting residues represented as capped sticks.

We solved the crystal structure of pro/CspB at 1.94 Å resolution (see methods and Supplementary Table 2) (Fig. 3a). The protein is arranged as a homodimer with a pseudo-binary axis and a modular architecture that resembles that of *C. perfringens* CspB (PDB ID: 4I0W)^18^ showing a Cα RMSD of ~2.5 Å (over 440 aligned residues). However, as detailed below, relevant differences are observed between the two orthologs (Supplementary Fig. 2). Both proteins adopt a subtilisin-like fold, consistent with their classification within the subtilisin family of serine proteases. Also, as observed in the *C. perfringens* case, the catalytic domain of pro/CspB (residues 68–256 and 395–537) is interrupted by a 137-amino-acid insertion that folds into a β-barrel jelly-roll domain, structurally integrated into the protease core. Importantly, the crystal structure shows that CspB remains bound to its prodomain (residues 5–65), following co-expression of the genes for both the prodomain and the mature CspB. The prodomain is known to function as an intramolecular chaperone^18^. Accordingly, when only the gene for the mature enzyme was expressed, the protein precipitated as inclusion bodies, while co-expression of the prodomain restores proper folding of the catalytic domain (Supplementary Methods). The catalytic triad—residues D96, H160, and S461 (Fig. 3b)—is embedded within a canonical subtilisin architecture, where an eight-stranded parallel β-sheet is flanked by four α-helices that form a conserved scaffold stabilizing the active-site geometry. The active sites present differences between *C. difficile* and *C. perfringens* in loop L_378-382_ (residues 378–382) of the jelly-roll domain, and in the loop L_183-190_ (residues 183–190) (box 1 in Supplementary Fig. 2a). These changes affect the active-site cavity, where the *C*-terminal segment of the prodomain is positioned. Additional differences are observed in the catalytic domain of *C. difficile* protein (boxes 2–4 in Supplementary Fig. 2a) involving structural variations in specific loops, such as L_β2-β3_ (residues 120–135), as well as an altered conformation of the *C*-terminal region. These structural variations have implications for domain organization and oligomeric assembly, respectively.

The prodomain of CspB adopts a four-stranded antiparallel β-sheet, followed by two α-helices (Fig. 3a). Its *C*-terminal coil segment extends into the catalytic cleft, sterically occluding access to the active site, which renders the enzyme inactive (Fig. 3b). Comparison with the *C. perfringens* prodomain reveals notable structural differences between the two prodomains (box 5 Supplementary Fig. 2a): (i) the *C. perfringens* prodomain exhibits a longer *N*-terminal extension, including an additional α-helix absent in *C. difficile*; and (ii) the *C*-terminal region of the *C. perfringens* prodomain harbors an extra residue (S96) that penetrates more deeply into the catalytic cleft, where the two terminal residues (S96 and T95) are accommodated within the S1 and S2 binding subsites, respectively^18^. In contrast, in *C. difficile*, only the S2 site is occupied by T65 (Fig. 3b).

The catalytic domain and prodomain of *C. difficile* CspB are tightly associated through extensive van der Waals contacts, hydrogen bonds, and electrostatic interactions. Notably, three arginine residues in the catalytic domain (R192, R198, and R132) form a salt-bridge network with the prodomain (Fig. 3b), contributing to the stability of the complex. Interestingly, only one of these residues (R198) is conserved in *C. perfringens*; R192 is substituted by a leucine (L225), and R132 is located within the L_β2-β3_ loop, which is absent in *C. perfringens* (Supplementary Fig. 2a). Previous studies have demonstrated that these electrostatic interactions are important for maintaining the structural integrity and ensuring the proper folding of the enzyme during maturation^18^.

The β-barrel jelly-roll domain comprises nine antiparallel β-strands that assemble into a compact hydrophobic core. Another notable difference lies in the modular arrangement of *C. difficile* and *C. perfringens* pro/CspB. In *C. perfringens*, the jelly-roll domain establishes few interactions with the prodomain. In contrast, the more compact architecture of the prodomain in *C. difficile* (box 5 in Supplementary Fig. 2), combined with the extended *C*-terminal region of the catalytic domain (box 4 in Supplementary Fig. 2), allows the *C*-terminal segment of the catalytic domain to insert into the interface between the jelly-roll domain and the prodomain of the dimeric partner (Fig. 3c). This creates a strong dimeric structure that is likely to be biologically relevant, as it may mitigate the detrimental effects of environmental stressors, such as heat and desiccation, which are typically encountered by dormant spores.

Given that the prodomain was non-covalently bound to the CspB active site, we investigated whether deletion of the residues accommodated in the S2 binding subsite would allow pro/CspB to process proSleC into SleC (see Supplementary Methods). Several *C*-terminal deletions encompassing 3, 6, or 9 amino acids were generated, resulting in the following constructs proΔ3/CspB, proΔ6/CspB, and proΔ9/CspB. The constructs proΔ6/CspB and proΔ9/CspB exhibited poor solubility. Moreover, the prodomain was found to be essential for CspB stability, as the proΔ6/CspB and proΔ9/CspB underwent complete degradation by YabG (the protease responsible for processing proCspBA into CspA and pro/CspB). In contrast, the co-expressed full-length pro/CspB and the proΔ3/CspB constructs remained stable against the YabG activity, underscoring the critical role of the prodomain in maintaining both solubility and stability (Supplementary Fig. 3). Finally, we tested whether these constructs could restore CspB protease activity toward proSleC. Only weak activity was observed, and this was detectable in the presence of 1 mM ZnCl₂ (Supplementary Fig. 4).

### Cryo-EM structure of pro/CspB

The crystal structure of pro/CspB revealed that the prodomain stably associated with the catalytic domain. This structural observation prompts important questions about the physiological state of CspB in dormant spores. Specifically, does the prodomain remain bound to the autoprocessed protease under native conditions, as it does in vitro? If so, what molecular events trigger its release during spore germination? Given that access of the substrate proSleC to the active site would require the prodomain displacement, these findings suggest that a pronounced increase in conformational plasticity of CspB may be a prerequisite for its activation. To assess whether pro/CspB adopts multiple structural states under more native-like conditions, we solved its structure by cryo-EM (Supplementary Table 3 and Supplementary Fig. 5). The cryo-EM reconstruction (3.2 Å resolution, Supplementary Fig. 6) was virtually indistinguishable from the crystallographic structure, with no significant deviations in the global fold or domain organization (RMSD of 0.395 Å over 1029 Cα aligned residues) (Supplementary Fig. 2b). This high degree of structural congruence suggests that pro/CspB adopts a rigid and well-defined conformation.

During micrograph acquisition for pro/CspB single-particle cryo-EM, we observed that the protein was capable of forming a supramolecular assembly comprised of dimers of pro/CspB organized in a helical arrangement of six dimers per helical turn (Fig. 3d, e). These oligomers exhibited a defined and extended architecture, characterized by a notably linear arrangement without apparent curvature, suggesting a relatively rigid conformation. The oligomeric species was consistently observed at a protein concentration of 18 mg/mL (291 µM), which was used for grid preparation.

Three-dimensional reconstruction of the oligomers at 2.75 Å resolution (Supplementary Fig. 6 and Supplementary Fig. 7) revealed that pro/CspB dimers assemble into fibrils with helical symmetry (Fig. 3e). The fiber structure can be described as a three-stranded right-handed superhelix with a diameter of ~128 Å. The core of the oligomer is hollow, giving rise to an internal channel of ~30 Å diameter that runs along the longitudinal axis of the assembly. The entire reconstructed density can be unambiguously accounted for by the arrangement of pro/CspB dimers (Supplementary Fig. 8a). Throughout the fibril, each prodomain and active site (white arrows in Fig. 3e) is exposed to the milieu, whereby dissociation of the prodomain will make the active site accessible to its substrate. Focusing on a single pro/CspB dimer, oligomer stabilization is mediated by three distinct types of inter-subunit interactions: (i) intra-chain interface, linking the catalytic domains of the pro/CspB dimers within a growing strand (Fig. 3f), (ii) inter-chain interface 1, connecting two chains via interactions between the jelly-roll domains from different strands (Fig. 3g), and (iii) inter-chain interface 2, connecting two strands through interactions between the jelly-roll domain of one chain and the catalytic domain of another (Fig. 3h). The intra-chain interface is predominantly hydrophobic, centered on a β-hairpin (residues 449–457). Residues such as L159, L457, L449, or A456 form a flat hydrophobic surface that engages the corresponding β-hairpin of the adjacent catalytic domain (Fig. 3f). Inter-chain interface 1 involves the same set of residues from the jelly-roll domains of adjacent strands (Fig. 3g). This interface is characterized by hydrophobic cores formed by I277, P304, P356, L390, L358, L390 and I391, which face each other, complemented by polar interactions involving N306, E280, E273, K360, N386, and E280. A potential salt-bridge between R275 and R388 may further stabilize the interface. The inter-chain interface 2 links the jelly roll of one chain with the catalytic domain of an adjacent chain (Fig. 3e, h). This interface specifically involves residues E364, N269, N530, Y526, or N81, among others, which are connected through polar contacts, with additional van der Waals interactions contributing to interface stability (Fig. 3h).

Comparisons among the dimeric components of the fibrils and the cryo-EM and crystallographic structures of dimeric pro/CspB indicate that the overall protein conformation is largely preserved, independent of the structural context. The active site in each subunit of pro/CspB fibrils is situated in a surface-exposed cleft, remaining solvent-accessible, which suggests that the fibrils could retain catalytic competence upon prodomain dissociation.

### Three-dimensional structure of CspA:taurocholate complex

**Fig. 4:**
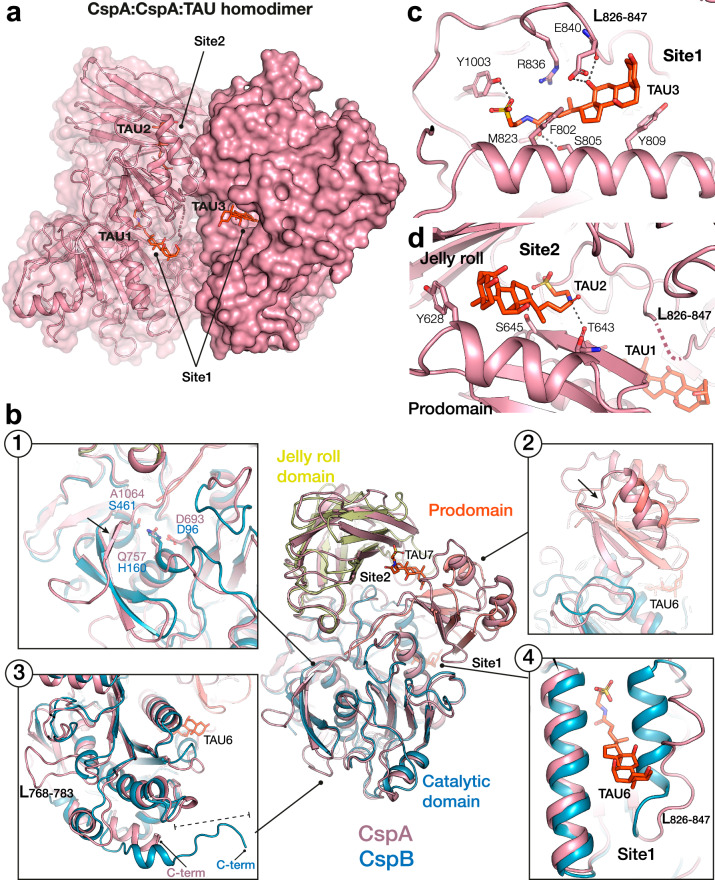
Three-dimensional structure of the CspA:taurocholate complex. **a** Crystal structure of the CspA:CspA:taurocholate (CspA:CspA:TAU) homodimer is shown. Ribbon representation and transparent surface for one chain are shown, with the opaque surface for the other chain in the dimer. The taurocholate ligands are depicted as capped sticks. **b** Structural comparison of pro/CspB and CspA monomers is given. pro/CspB is shown as a ribbon representation with domains colored: catalytic domain (in blue), jelly-roll domain (in green), and prodomain (in coral); CspA is colored in pink. Boxed areas highlight (1) active site, (2) prodomain, (3) catalytic domain (differences in the *C*-terminal ends are indicated by a dashed line), and (4) the taurocholate-binding site (Site 1) in CspA is compared to the equivalent region in pro/CspB. **c** A detailed view of taurocholate recognition by CspA in Site1 is given. Key residues forming the ligand-binding site are shown as capped sticks and labeled. **d** A detailed view of taurocholate recognition by CspA in Site 2 is given. Key residues forming the ligand-binding site are shown as capped sticks and labeled.

The crystal structure of CspA in complex with taurocholate (TAU) was determined at 2.9 Å resolution (Fig. 4a and Supplementary Fig. 9) after incubation of CspA with taurocholate. Six independent CspA chains were present in the asymmetric unit of the crystal. Four of them were arranged as homodimers and two as monomers. This distribution in dimers and monomers agrees with our observation on the effect of taurocholate in disturbing the dimeric arrangement of CspA. In all cases, CspA showed a domain arrangement similar to pro/CspB (Supplementary Fig. 2c), comprising a prodomain, a jelly-roll domain, and a catalytic-like pseudoprotease domain. Remarkably, a variable binding of taurocholate molecules was observed among the CspA dimers and monomers (Supplementary Fig. 9). One dimer allocates three and the other two taurocholate molecules, and in the monomers, one of them contained two and the other just one taurocholate. In all cases the electron density allowed perfect identification of the bile salt (Supplementary Fig. 9a). Most of taurocholate molecules (6 of 8) occupied a pocket at the interface between the catalytic domain and the prodomain (Fig. 4a) that we designated as Site 1, while the remaining two occupied a pocket, Site 2, at the interface between the prodomain and the jelly-roll domain (Fig. 4a). Site 1 is primarily formed by hydrophobic residues, stabilizing the steroidal scaffold of the taurocholate molecule through van der Waals interactions (Fig. 4c). The quaternary amine and the sulfonate groups of taurocholate are inserted into the pocket of the protein. Loop L_826-847_ in CspA presents charged residues (E840, R836) that establish polar interactions with the taurocholate molecule. This loop, important in taurocholate recognition, presents a flexible conformation in CspA (Supplementary Fig. 9b) and, in two of the six independent chains, their backbone was not visible in the electron-density map. Two taurocholate molecules were found in Site 2; remarkably, the molecules are sandwiched between the jelly roll and the prodomain, but with no specific interactions, which resulted in two different arrangements of the taurocholate molecules (Fig. 4d and Supplementary Fig. 9c).

Although the jelly-roll domains of both CspA and CspB are largely similar, relevant differences are observed (Fig. 4b). As expected, the pseudoprotease CspA lacks the catalytic triad (Fig. 4b1). Consequently, the linker connecting the pseudo-catalytic domain to the prodomain remains intact, fully occluding the active site. Part of this region adopts a small β strand (arrow in Fig. 4b1) that packs against the β hairpin of the catalytic domain. The prodomain in CspA contains shorter β-strands and larger α-helices compared to the homologous region in pro/CspB (Fig. 4b2), with an additional insertion (arrow in Fig. 4b2) inserted into the fold that is absent in CspB prodomain. Importantly, the strong network of salt-bridge interactions that connects the prodomain and the catalytic domain in pro/CspB (Fig. 3b) is absent in CspA; in this case, the primary interactions between the domains are hydrophobic. Differences are also apparent in the pseudo-catalytic domain of CspA compared to pro/CspB, the most notable being an insertion comprising residues 768-783 (L_768-783_) and a much shorter *C*-terminal region (Fig. 4b3). In contrast, in pro/CspB, the extended *C*-terminal segment of one monomer inserts as a wedge between the jelly-roll domain and prodomain of the partner monomer, establishing an extensive network of interactions that stabilize the dimer (Fig. 3c). This arrangement reinforces the dimer and fixes the position of the prodomain. However, in CspA the *C*-terminus is shorter (Fig. 4b3), resulting in (i) a weaker pattern of inter-monomer interactions within the CspA dimer compared to pro/CspB, and ii) a shift of the CspA prodomain towards the jelly-roll domain (in the absence of the *C*-terminal tail from the dimeric partner seen in pro/CspB). This altered prodomain position, together with additional structural features (see below), forms the pocket in which taurocholate is bound.

The other key difference between the catalytic domain of pro/CspB and its counterpart in CspA is that CspA lacks one of the four α-helices typical of the canonical subtilisin fold. Specifically, the α-helix spanning residues 232-242 in pro/CspB is replaced by loop L_826-847_ in CspA (Fig. 4b4), which adopts a more open conformation capable of accommodating a taurocholate molecule (Site 1, Fig. 4c).

### Three-dimensional Structure of CspA:CspC:taurocholate Complex

**Fig. 5:**
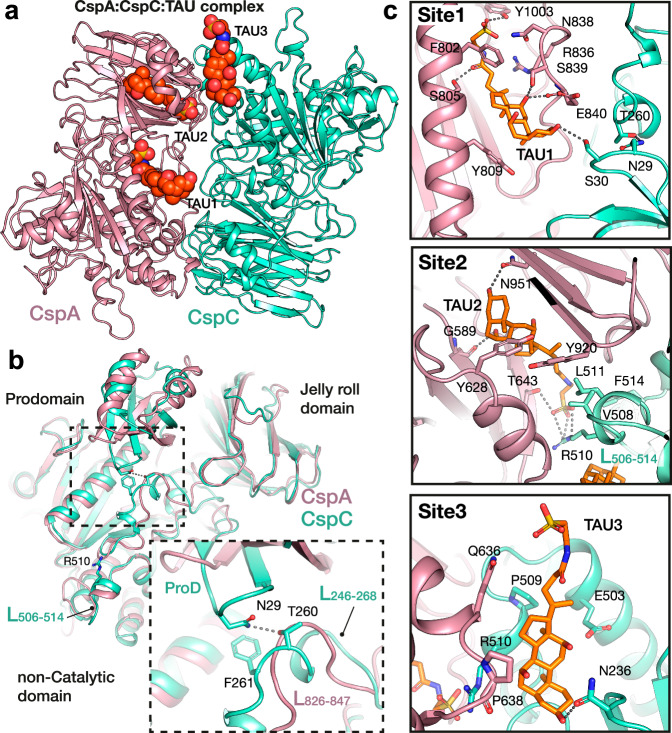
Three-dimensional structure of the CspA:CspC:taurocholate complex. **a** Cryo-EM structure of the CspA:CspC:taurocholate (CspA:CspC:TAU) heterodimer is shown, with ribbon representation of CspA (pink) and CspC (green). Taurocholate (TAU) is represented as spheres. **b** Structural comparison of CspA and CspC monomers is given. The boxed region highlights the expanded connection between the prodomain and the loop L_246-268_ in CspC. **c** Close-up views of taurocholate-binding sites in the CspA:CspC complex are given. Key residues shaping the taurocholate-binding pockets are represented as capped sticks.

The CspA:CspC complex was produced, and its structure was determined by cryo-EM at 2.75 Å resolution (Supplementary Table 3 and Supplementary Fig. 6). The complex was incubated with taurocholate at 10 mM. The three-dimensional structure (Fig. 5a) reveals the heterodimer bound to three taurocholate molecules, which are clearly resolved in the cryo-EM density (Supplementary Fig. 8b). The heterodimer adopts a similar architecture to that observed in the pro/CspB and CspA homodimers, with the two proteins related by a pseudo-two-fold symmetry. The three-dimensional structure of CspA within the heterodimer closely resembles that in the homodimer (RMSD 0.33 Å for 448 Cα atoms superposition), with differences limited to the loop (L_905-922_) of the jelly-roll domain and loop L_826-847_ of the catalytic domain. As described below, both changes correspond with the taurocholate-binding Site 1 and Site 2. The other component, CspC, is also a non-catalytic protease (as is CspA); its catalytic triad is replaced (G485, T170, D105) relative to CspB (S461, H160, and D96). This structure closely matches the crystal structure of monomeric CspC (PDB 6MW4)^19^ (RMSD of 0.37 Å for 452 Cα atoms superposition), with minor differences observed in loops L_360-369_ and L_451-459_ (Supplementary Fig. 2d). Interestingly, this flexible region (L_451-459_) in the monomeric structure becomes stabilized upon heterodimer formation with CspA.

Structural comparison of CspA and CspC indicates that, despite an overall similar fold (RMSD of 1.01 Å for 393 Cα atoms superimposition), notable differences in modular arrangement are apparent (Fig. 5b). Significant variations are observed in the prodomain of CspC, which approaches loop 246-268 (L_246-268_) (equivalent to L_826-847_ in CspA) (Fig. 5b), forming polar (N29-T260) and van der Waals interactions between these regions (boxed in Fig. 5b). Additional differences are found in loop 506-514 (L_506-514_), containing R510 (Fig. 5b). Collectively, these structural distinctions among CspA, pro/CspB and CspC are important for the specific recognition of taurocholate.

Three different taurocholate molecules were identified bound to the CspA:CspC complex (Fig. 5a and Supplementary Fig. 10a), each occupying a distinct binding pocket, two of them previously seen in CspA (Sites 1 and 2), and a new one, Site 3, only found in the heterodimer (Fig. 5c). In Site 1 of the heterodimer, the steroidal backbone of taurocholate (TAU1) is stabilized by van der Waals interactions with hydrophobic residues within the pocket, analogous to taurocholate binding in the CspA homodimer. However, in the CspA:CspC heterodimer taurocholate binding also establishes multiple polar interactions, both with the steroid nucleus and the taurine side chain (Fig. 5c). Most of these polar interactions involve residues from CspA, with an additional contribution from S30 of CspC prodomain (Fig. 5c).

In Site 2, positioned at the interface between the prodomain and the jelly-roll domain of CspA, the taurocholate molecule is sandwiched between these two domains, with the loop L_506-514_ from CspC occluding the site entrance (Fig. 5c and Supplementary Fig. 10b). The taurocholate molecule (TAU2) is stabilized through van der Waals interactions (Y628, Y920) within the CspA pocket, complemented by hydrogen-bond interactions involving N951, G589, and T643. The L_506-514_ region from CspC plays a role in stabilizing taurocholate at Site 2 through van der Waals interactions involving residues V508, L511 and F514, and most notably, through electrostatic interactions between R510 and the negatively charged sulfonate group of the taurine moiety (Fig. 5c). It is worth mentioning that this orientation of the taurocholate molecule at Site 2 is distinct to the heterodimer, and it is different from those observed in the CspA:taurocholate complex.

Site 3 (Fig. 5c) is located at the interface between CspC and CspA, specifically involving the L_506-514_ region of CspC and the loop L_636-640_ of the CspA prodomain. In this site, the taurocholate molecule (TAU3) displays its taurine moiety exposed to the solvent, with no detectable interaction with the protein, as evidenced by the weak electron density accounting for this region (Supplementary Fig. 8b), whereas its steroidal backbone is stabilized by van der Waals and polar interactions (Fig. 5c).

Mutations in CspC, including substitutions at Gly457, Arg456, and Asp429, have been shown to alter taurocholate recognition^30,31^. However, structural analyses suggest that these residues are not directly involved in taurocholate binding; instead, they play a role in stabilizing the CspA:CspC heterodimer (Supplementary Fig. 11a).

In summary, our structural data reveal that CspA recognizes taurocholate at Sites 1 and 2, formed in cooperation with CspC. Site 3, located at the interface of the inactive catalytic domain of CspC and the prodomain of CspA stabilize the steroid moiety of the third taurocholate molecule. The distinct structural features of CspA—including the flexible L_826–847_ region and the distinct structure and rearrangement of its prodomain—form the template for germinant binding. This process additionally relies on the complementarity of specific regions in CspC, with the L_506–514_ segment being particularly important for full taurocholate recognition. We hasten to add that future mutational analysis based on the structural knowledge disclosed herein is needed to address the physiological relevance of these models.

## Discussion

We have described in this report the biophysical and structural characterizations of CspA, CspB, and CspC, three proteins at the core of a cascade of events leading to germination of spores in *C. difficile*. The process is highly regulated, encompassing transcription of the bicistronic *cspBA-cspC* operon, production of the precursor proteins requiring proteolytic activation, and assembly of the functional complex that serves as a receptor for the germinant taurocholate. Interestingly, glycine, the co-germinant, did not bind to any of these proteins individually, nor to the CspA:CspC complex, but it bound to the CspA:CspC:taurocholate assembly.

CspA, CspB, and CspC share a conserved overall fold, characterized by a catalytic domain (only CspB presents a functional catalytic triad), the jelly-roll domain, and a prodomain. However, structural differences in certain regions (such as L_826–847_ in CspA, L_506–514_ in CspC or the C-terminal extension in CspB), influences the formation of the oligomers and recognition of the germinant. Taurocholate induces formation of the CspA:CspC complex, which exhibits a remarkable dissociation constant of 97 ± 8 pM.

Upon expression of the bicistronic gene construct *cspBA-cspC* and synthesis of proCspBA and CspC, the protease YabG generates proCspB and CspA. CspA and CspC subsequently assemble into the heterodimer characterized in this report. Meanwhile, proCspB undergoes autocleavage to yield a complex of the prodomain and mature CspB (pro/CspB). The prodomain functions as a chaperone, stabilizing the protein structure, which exists in solution as a homodimer. Notably, the pro/CspB dimer organizes into supramolecular fibrils forming a three-stranded, right-handed superhelix, with the fibril lengths reaching several hundred Ångstroms. The active site of CspB would be available for its catalytic function upon dissociation of the non-covalently bound prodomain. The functional significance of the fibril remains unclear. We speculate that while the intrinsic ability of the CspB homodimer to form supramolecular assemblies is inherent, the dehydrated environment within the spores may promote fibril formation.

A key finding is the mechanism of taurocholate recognition at three distinct sites within the CspA:CspC complex. Structural comparison among CspA, CspB, and CspC (Supplementary Fig. 10c) indicates that Site 1 is accessible only in CspA, as the corresponding region is occluded in CspB and CspC. Similarly, Site 2 is also exclusive to CspA, since in the CspB homodimer the equivalent site is occluded by the prodomain and the *C*-terminal extension of the dimeric partner (upper left box in Supplementary Fig. 10c), and in CspC the Site 2 is occupied by bulky residues from the prodomain and jelly-roll domains (upper right box in Supplementary Fig. 10c). Finally, Site 3 is formed at the interface of CspA:CspC heterodimer that is not available in any homodimer. The heterodimer is required to reach full recognition of taurocholate: loop L_260-269_ of CspC provokes a change in the conformation of L_826–847_ in CspA (Supplementary Fig. 11b) that orients the side chains to create the interaction pattern described in Fig. 5c. This specific conformation for L_826–847_ is not observed in any of the six independent CspA chains of the CspA:taurocholate complex. Also, Sites 2 and 3 would require the CspA:CspC heterodimer to allow taurocholate binding, as corroborated by the CspA:taurocholate complex. In summary, while CspA can bind taurocholate molecules both as a monomer or as a homodimer, the full potential for binding is reached only for the CspA:CspC heterodimer. We acknowledge that the biological relevance of these interactions should be explored by mutational analysis in *C. difficile* and in association with structural characterization of the mutant variants in support of the model that we have proposed in this report. We hasten to add that while our manuscript was in review a report of the structure of the homodimer of CspA and of the heterodimer of CspA:CspC appeared in the literature, in which some mutational analyses were indeed carried out^32^. No ligands were shown to be associated with these structures, nonetheless, their findings are consistent with ours as reported herein.

**Fig. 6:**
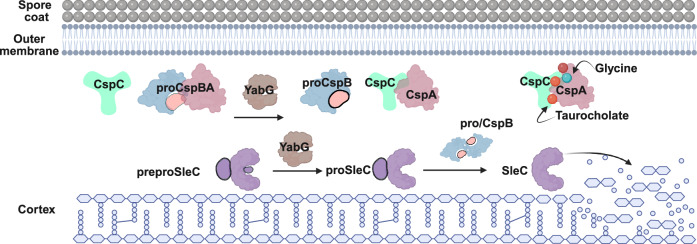
Proposed mechanistic model of *C. difficile* germination. Once proCspBA, a fusion protein, is produced, it serves as a substrate for YabG, which proteolyzes it to produce proCspB and CspA. On formation of CspA, it binds to CspC (the picomolar union), followed by the assembly of the three taurocholates and of glycine, which signals the onset of germination. With proCspB formed, it undergoes autolysis of the prodomain, which remains associated with the protein, leading to dimerization and oligomerization. The release of the prodomain by an unknown process sets in motion the processing of proSleC to initiate degradation of spore-cortex peptidoglycan. Created in BioRender. Mobashery, S. (2026) https://BioRender.com/kaurm53.

Figure 6 summarizes our proposed working model based on the biophysical and structural data presented here. This schematic integrates the formation of the CspA:CspC heterodimer and the three taurocholate-binding sites. The model depicts the early steps of the cascade during sporulation, namely YabG processing of proCspBA and preproSleC to generate CspA, proCspB, and proSleC. Importantly, taurocholate binding to the CspA:CspC complex promotes binding of glycine, preceded by CspB processing of proSleC, thereby initiating degradation of the cortex peptidoglycan.

The complexity of the germinosome has hindered a full understanding of the sequence of events leading to spore germination in *C. difficile*. Here, we reveal biophysical and structural information of key events that are central to the onset of these processes.

## Methods

### Cloning

For improved production of recombinant CspB in *E. coli*, the *cspB* segment in the *cspBA* gene (CD630_22470) encoding the full-length CspB (proCspB) in the strain *C. difficile* 630 was codon-optimized and synthesized at GenScript Biotech (Piscataway, NJ). The synthetic *cspB* gene was first inserted into an expression vector pETHST at the restriction sites NdeI and XhoI^33^ to generate pETHST-proCspB6H. To remove the *N*-terminal HST tags, the *cspB* gene was relocated into pET24a using the same restriction enzymes mentioned above to produce the *C*-terminal 6×His-tagged proCspB. The resulting plasmid was named pET24a-proCspB6H. To create a plasmid that co-expresses the prodomain and the mature CspB, a stop codon, a ribosome binding site and a start codon were introduced in the gene segment between T65 (the *C*-terminus of the prodomain) and Q66 (the *N*-terminus of the mature CspB) by amplifying pET24a-proCspB6H with the primers CK457 and CK458 (listed in Supplementary Table 1), then the KLD cloning kit (New England Biolab, Boston, MA) was used to generate pET24a-pro/CspB6H. Three amino acids, six amino acids, and nine amino acids from the *C*-terminus of the prodomain of pro/CspB6H were deleted by amplifying pET24a-pro/CspB6H with the pairs of primers, CK503/CK504, CK503/CK505, and CK503/CK506, respectively. The resulting plasmids pET24a-proΔ3/CspB6H, pET24a-proΔ6/CspB6H and pET24a-proΔ6/CspB6H were created by the KLD cloning kit. For SPR analysis, the *N*-terminal 6×His-tag was deleted from pETHST-proCspB6H by amplifying the plasmid with the primers CK492/CK493, followed by KLD cloning, resulting in the generation of pST-proCspB6H. This plasmid expressed proCspB fused with a Twin-Strep tag and a TEV cleavage site at the *N*-terminus and a 6×His-tag at the *C*-terminus (ST-proCspB6H).

An expression vector pET28a-CPDsal (Addgene plasmid # 38252) was used to clone *cspA*. The *cspA* segment in the *cspBA* gene was amplified from the genomic DNA of the strain *C. difficile 630* with the primers CK421/CK422. The vector and the PCR product were digested with the restriction enzymes NcoI and SalI, followed by gel extraction of the correct DNA fragments, and ligation of the two fragments by T4 DNA ligase. The resulting plasmid was named pET28a-CspA-CPD6H, producing CspA fused with 6×His-tagged cysteine protease domain (CPD6H) at the *C*-terminus^34^. A HiFi assembly approach was used to generate ST-CspA. Primers CCL_13 and CCL_14 amplified the *cspA-cpd* gene from pET28a-CspA-CPD6H, and the primers CK322 and CK287 were used to amplify the vector backbone pST from pST-proCspB6H. The HiFi assembly generated the vector pST-CspA-CPD lacking a *C-*terminus 6×His-tag. To add a 6×His-tag at the *C*-terminus, primers CCL_15 and CCL_16 were used, resulting in the vector pST-CspA-CPD6H. The KLD kit was used to relocate the Twin-Strep-tag to the *C-*terminus of CspA with primers CCL_22 and CCL_23 and the pET28a-CspA-CPD6H vector as DNA template. The PCR generated the vector CspA-ST-CPD6H.

Prof. Aimee Shen generously gifted us pET22b-CspC-CPD6H, in which the *cspC* gene (CD630_22460) is codon-optimized for *E. coli*. This plasmid produces CspC fused with CPD6H at the *C*-terminus. To generate ST-CspC, a restriction enzyme cloning approach was used. 1 µg each of ST-proCspB6H and pET22b-CspC-CPD6H was digested with enzymes NdeI and XhoI. The ST vector backbone and the *cspc-cpd* insert were gel-purified. Afterward, a ligation reaction was employed to generate pST-CspC-CPD6H.

In order to purify the full-length YabG in *E. coli*, the *yabG* gene (CD630_35690) was amplified with the primers CK423/CK424 from the genomic DNA of the *C. difficile* 630 strain. The PCR product and a vector pET24d were digested with NcoI and XhoI, followed by gel extraction of the appropriate fragments and their ligation by T4 DNA ligase.

### Protein expression

Most of the recombinant proteins were expressed with a standard procedure for IPTG induction. Briefly, the overnight cultures were diluted 1000- or 100-fold in 1 L of fresh Terrific Broth (TB) containing appropriate antibiotics depending on the vectors and the strains (100 µg/mL of ampicillin for pET22b, 100 µg/mL of kanamycin for pETHST, pET24a, pET24d, pET28a-CPDsal and pST, and 30 µg/mL of chloramphenicol for LOBSTR-RIL), followed by incubation at 37 °C with shaking until OD_600_ reaches between 0.5 and 0.7. After the incubation, the cultures were cooled down to room temperature, and isopropyl-β-D-1-thiogalactopyranoside (IPTG) was added to a final concentration of 0.5 mM to induce protein production. The cultures were further incubated overnight with shaking at 180 rpm at several different temperatures depending on the constructs: 16 °C for CspA-CPD6H, ST-CspA-CPD6H and CspA-ST-CPD6H; 18 °C for HST-preproSleC, SleC-CPD6H, proΔ3/CspB6H, proΔ6/CspB6H and proΔ9/CspB6H; 20 °C for proCspB6H, ST-proCspB and pro/CspB6H. The cells were harvested by centrifugation at 7000×*g* for 20 min at 16 °C, and stored at −80 °C until use.

CspC-CPD6H and ST-CspC-CPD6H were produced in *E. coli* BL21(DE3) following the auto-induction method previously reported^19^. Briefly, *E. coli* BL21(DE3) carrying pET22b-CspC-CPD6H was streaked on an LB agar plate containing 100 µg/mL ampicillin. A single colony was inoculated in 20 mL of LB medium. After a 4 h-incubation at 37 °C, 1 mL of the culture was inoculated in 1 L of TB supplemented with 5052 sugar mix (0.5% glycerol, 0.05% glucose, and 0.1% α-lactose), followed by incubation at 20 °C for 60 h. The cells were harvested by centrifugation at 7000×*g* for 20 min at 16 °C, and stored at −80 °C until use.

YabG is toxic to *E. coli* when it is produced because it is a protease. In order to produce YabG6H, the overnight culture was diluted 50-fold in 2 L of TB containing 50 µg/mL of kanamycin and 20 µg/mL of chloramphenicol, followed by incubation at 37 °C until OD_600_ reached 0.5 (~5 h). After the addition of 0.5 mM of IPTG, the culture was kept at 37 °C for 2 hours with shaking at 180 rpm, and harvested at 7000×*g* for 20 min at 16 °C. The cell pellet was immediately used for the purification of YabG6H.

### Protein purification

All proteins fused with a 6×His-tag were purified using a two-step Ni-NTA affinity and diethylaminoethyl (DEAE)-anion-exchange chromatography. The proteins fused with CPD6H underwent two additional steps: autocleavage of CPD6H in the presence of phytic acid and Ni-NTA affinity chromatography to separate uncut CPD6H-fused proteins and CDP6H from the proteins without CDP6H^34^. All purification procedures were performed at 4 °C. Briefly, the cells were resuspended in 30 mL of lysis buffer (50 mM HEPES, pH 7.4, 500 mM NaCl, 5% (w/v) glycerol, 100 µg/mL DNase I, 100 µg/mL lysozyme, 10 mM MgCl_2_, and 5 mM β-mercaptoethanol). Cells were lysed by passing them twice through a French press at 800 psi, followed by centrifugation at 17,000×*g* for 1 hour at 4 °C to separate unbroken cells and insoluble fraction from the soluble fraction containing the proteins. The supernatant was passed through a 0.2-µm filter membrane to remove any residual insoluble materials. The clear lysate was loaded on 5 mL of Ni-NTA resin, which was pre-equilibrated with at least five column volume (CV) of Buffer A (50 mM HEPES, pH 7.4, 500 mM NaCl, 5% (w/v) glycerol, 20 mM imidazole, and 5 mM β-mercaptoethanol), at a flow rate of 1 mL/min. The column was washed with 20 column volume of Buffer A, followed by 30 column volumes of washing buffer (50 mM PIPES, pH 6.5, 500 mM NaCl, 20% (v/v) glycerol, 50 mM imidazole, and 5 mM β-mercaptoethanol), followed by 10-column volumes of Buffer A. The proteins were eluted using an imidazole gradient from 20 to 300 mM. The fractions containing the targeted proteins were concentrated to 15 mL and buffer-exchanged to Buffer B (50 mM HEPES, pH 7.4, 100 mM NaCl, 5% (w/v) glycerol, and 5 mM β-mercaptoethanol). The sample was applied to 50 mL of the DEAE resin equilibrated with Buffer B at a flow rate of 1 mL/min. The column was washed with 6-column volumes of Buffer B. The proteins were eluted with a salt gradient from 100 to 700 mM NaCl. Fractions containing proteins that were not fused to the CPD6H domain were pooled, concentrated and buffer-exchanged to the storage buffer (50 mM HEPES pH 7.4, 150 mM NaCl, 5% (w/v) glycerol, 5 mM β-mercaptoethanol). Protein purity was assessed by SDS-PAGE, and purified proteins were flash-frozen under liquid nitrogen and stored at −80 °C until used. In contrast, fractions containing CPD6H-tagged proteins were pooled, concentrated, and buffer-exchanged to Buffer A. To proceed with the autocleavage of CPD6H, phytic acid was added to a final concentration of 0.5 mM and incubated at room temperature for 4 hours, followed by a 1-h incubation at 4 °C with Ni-NTA resin equilibrated with Buffer A. Cleaved proteins (lacking CPD6H tag) were separated from uncut proteins and CPD6H by collecting the flow-through and washing the resin batch-wise three times. The desired proteins were found in the flow-through and washes, and they were concentrated from 40 to 100 µM and buffer-exchanged to storage buffer. Protein purity was assessed by SDS-PAGE, and purified proteins were flash-frozen under liquid nitrogen and stored at −80 °C until used.

### Analytical ultracentrifugation (AUC)

Samples were centrifuged at 21,130×*g* for 10 min at 4 °C. Sedimentation velocity experiments were performed using a ProteomeLab XL-I AUC (Beckman Coulter) at 128,419×*g* and 20 °C using an AN-50 Ti rotor. The samples were loaded into cells equipped with 1.2-cm charcoal-epon double-sector centerpieces and quartz windows. The loaded rotor was equilibrated at rest for 75 minutes before starting the experiment. Absorbance at 230 or 280 nm was measured with 0.003 cm radial step size and no delay between scans. The protein’s partial specific volume and buffer (50 mM Na_2_HPO_4_, pH 7, 150 mM NaCl) density and viscosity at 20 °C were calculated using SEDNTERP v3.0.4^35^. Data were analyzed using the continuous c(s) distribution and c(M) distribution models in Sedfit v16.1c^36^.

### Isothermal-titration calorimetry (ITC)

All calorimetric measurements were performed using a MicroCal PEAQ-ITC (Malvern Panalytical) instrument at 25 °C. Samples containing 50 µM Csp protein in 50 mM HEPES, pH 7.4, 150 mM NaCl were titrated with 1 mM sodium taurocholate or 100 mM glycine in 50 mM HEPES, pH 7.4, 150 mM NaCl. Before the first injection, the system was allowed to equilibrate until a baseline of 5 µcal/s was reached. The titration consisted of 19 ligand injections, with 0.4 µL for the first injection and 2 µL for the remaining injections, at 150-second intervals with stirring at 750 rpm. Control experiments to measure ligand heat of dilution were conducted by titrating the ligand into buffer alone. Binding parameters, including dissociation constant (*K*_d_), enthalpy change (ΔH), and binding stoichiometry (*n*), were determined by fitting the data using a one set of sites binding model in the PEAQ-ITC Analysis Software v1.41.1329 (Malvern Panalytical). Experiments were performed in triplicate.

### Surface plasmon resonance (SPR)

Binding kinetics between the Csp proteins were assessed by surface plasmon resonance (SPR) on a Biocore X100 (Cytiva Life Science) with SPR running buffer (10 mM HEPES, pH 7.4, 150 mM NaCl, 3 mM EDTA, and 0.005% Tween 20). When examining the influence of taurocholate on Csp protein interactions, the SPR running buffer was supplemented with 1 mM sodium taurocholate. Amine coupling was used to immobilize GFP-Twin-Strep-tag (ST-GFP) on flow cell 1 and Strep-Tactin XT on flow cell 2 following the instructions provided with the Twin-Strep-tag capture kit (IBA Lifesciences) on sensor chip CM5. ST-GFP was meant to suppress non-specific binding to the reference cell. Twin-Strep-tagged CspA, CspB, and CspC constructs were captured on flow cell 2 at 100–150 targeted response units (RU), after which analytes from the lowest concentration to the highest concentration were flowed over the captured Csp proteins at 30 µL/min. Prior to each experiment, the system was primed with three buffer cycles. To maintain consistent binding levels between replicates, Twin-Strep-tagged Csp proteins were freshly captured before each analyte injection. The pairwise interaction association phase was set at 180 s, and the dissociation at 600 s. For the captured ST-CspC and the CspA analyte, they were allowed to associate for 180 s and dissociate for 3600 s. CspA, at concentrations ranging from 0.24 to 30 nM, was injected at a flow rate of 30 µL/min. The surface was regenerated with a 60 s injection of 3 M guanidium hydrochloride. Kinetic parameters (*k*_on_, *k*_off_, and *K*_d_) were obtained through global kinetic fitting to a one-to-one binding model in Biacore X100 Evaluation Software v2.0.2. Experiments were done in triplicate. For the measurements of the interaction between the heterocomplex ST-CspC:CspA and sodium taurocholate and glycine, the complex was formed by diluting ST-CspC and CspA to 10 nM in 1×PBS and then concentrating back to 1 µM. In total, 1 µM of either CspA-ST or the ST-CspC:CspA heterocomplex was captured on the Strep-Tactin XT chip to ensure adequate signal. Sodium taurocholate, at concentrations ranging from 2 to 250 µM and glycine up to 100 mM, was injected over the captured proteins at a flow rate of 30 µL/min, using 1xPBS as the running buffer for sodium taurocholate and PBS supplemented with 0.005% Tween 20 as the running buffer for glycine. The association phase was set at 180 s and the dissociation at 600 s. The surface was regenerated with a 60 s injection of 3 M guanidium hydrochloride. The dissociation constant was determined by plotting the RU measured 90 s before the end of each injection against the analyte concentration, followed by steady-state affinity fitting using the Biacore X100 Evaluation Software v2.0.2. Assessment of the interactions of the ST-CspC:CspA:taurocholate complex with glycine was performed using the same setup described above, with the exception that the SPR running buffer (1×PBS) was supplemented with 1 mM taurocholate. Glycine (0.1–100 µM) was injected over the captured proteins at a flow rate of 30 µL/min. The data were fit to a two-state binding model using the Biacore X100 Evaluation Software v2.0.2. All experiments were performed in triplicate.

### Microscale thermophoresis (MST)

All MST analyses were performed as described previously with slight modification^37^. Cy5-NHS ester purchased from BroadPharm was used to label the target protein. Sodium taurocholate was used at 0.25 mM as the highest concentration to evaluate the interaction with the Csp proteins, because it interacts with cyanine dyes at 1 mM^27,28^. When examining the influence of taurocholate on Csp protein interactions, the MST buffer was supplemented with 0.25 mM sodium taurocholate. Dissociation constants (*K*_d_ values) were determined using MO. Affinity Analysis Software v2.3. Experiments were done in triplicate.

### Native-polyacrylamide gel electrophoresis

Native-polyacrylamide gel electrophoresis (Native-PAGE) was performed to investigate the oligomeric states of individual Csp proteins and the formation of the complexes among the Csp proteins. For the heterocomplexes of the Csp proteins, 1:1 mixtures of CspA-CspC, CspA-pro/CspB, and CspC-pro/CspB, in which 15 µg of each protein is contained in a volume of 20 µL, were incubated for one hour at 4 °C before mixing with 2× Tris-Glycine sample buffer (ThermoFisher Scientific). Individual proteins (15 µg in 20 µL) were also mixed with 2× Tris-Glycine sample buffer without pre-incubation. All samples were loaded on Novex Tris-Glycin gel (8-16%), and the proteins were separated by running Native-PAGE in Tris (25 mM)-Glycine (192 mM) running buffer, pH 8.3, at 80–100 V for 3 h at 4 °C. The NativeMARK unstained protein standard (ThermoFisher Scientific; 1236, 1048, 720, 480, 242, 146, 66, and 20 kDa from the top) was used as a molecular-weight marker for Native-PAG. When examining the influence of taurocholate on Csp protein interactions, the proteins were incubated with 0.1, 0.5, 2, and 10 mM sodium taurocholate. Uncropped gel scans are provided in the source data file.

### Crystallization of pro/CspB and CspA:taurocholate

Crystallization screenings were performed using high-throughput techniques in a NanoDrop robot and Innovadyne SD-2 microplates (Innovadyne Technologies, Inc.) with screening PACT Suite and JCSG Suite (Qiagen), JBScreen Classic 1 to 4 (Jena Bioscience), Morpheus and MIDASplus (Molecular Dimensions), and Crystal Screen I&II, SaltRX, and Index HT (Hampton Research). The conditions that produced crystals were optimized with a sitting-drop vapor-diffusion method at 291 K by mixing 1 µL of protein solution and 1 µL of precipitant solution, equilibrated against 150 µL of precipitant solution in the reservoir chamber. For pro/CspB, the best crystals were obtained in 0.2 M NaCl, 0.1 M sodium acetate (pH 4.6), and 30% MPD (2-Methyl-2,4-pentanediol). Protein was assayed at 9–10 mg/ml. For CspA:taurocholate, the best crystals were obtained in 0.2 M sodium sulfate and 20% PEG3350. Protein was assayed at 20 mg/ml. Taurocholate was used at 10 mM.

### X-ray data collection and structural determination of pro/CspB and CspA:taurocholate complex

Diffraction data were collected in the XALOC beamline at the ALBA synchrotron (Barcelona, Spain) using a Pilatus 6 M detector. Pro/CspB crystals diffracted up to 1.94 Å resolution and belonged to the C 2 2 2_1_ space group with the unit-cell parameters *a* = 101.91 Å, *b* = 115.73 Å, *c* = 201.69 Å and α=β=γ=90°. CspA:taurocholate crystals diffracted up to 2.90 Å and belonged to the P 2 2_1_ 2_1_ space group with the unit-cell parameters *a* = 141.24 Å, *b* = 158.63 Å, *c* = 188.36 Å and α=β=γ=90°. The collected data sets were processed with XDS v2024^36^ and Aimless v0.8.2^37^. In the pro/CspB crystals, two monomers were found in the asymmetric unit, yielding a Matthews coefficient^38^ of 2.41 Å^3^/Da and a solvent content of 48.91%. In the CspA:taurocholate crystals, 6 CspA chains were found in the asymmetric unit, yielding a Matthews coefficient^38^ of 2.75 Å^3^/Da and a solvent content of 55.26%. In both cases, the structure determination was performed using the molecular-replacement method implemented in PHASER v2.8.3^39^. For this, we used the predicted AlphaFold2 structure for monomeric CspA and dimeric CspB complexed with two prodomain copies. After locating each protein copy in the electron density, the model was manually completed using Coot v0.9.8.96^40^ and subjected to several iterative refinement cycles using PHENIX v1.21.2-5419^41^. Statistics for the crystallographic data and structure solution are summarized in Supplementary Table 2.

### Cryo-EM data collection and processing

In all cases, Cryo-EM grids were prepared by applying 3.6 μL of the sample solution onto glow-discharged Quantifoil Au 1.2/1.3 300 mesh electron microscope grids. The grids were subsequently plunge-frozen in liquid ethane using a Vitrobot Mark IV (Thermo Fisher Scientific). Data acquisition was performed at the CM01 beamline of the European Synchrotron Radiation Facility (ESRF) using a Titan Krios transmission electron microscope operated at 300 kV and equipped with a K3 direct electron detector, a Quantum LS energy filter (20 eV slit width), and a Volta phase plate (VPP). Super-resolution movies were recorded at a nominal physical pixel size of 0.839–0.840 Å/pixel, with an electron dose of approximately 1.0 e⁻/Å² per frame. Each movie comprised 40 frames, corresponding to a total cumulative dose of approximately 40 e⁻/Å² per image. Automated data collection was controlled using SerialEM v3.8.6 software.

Cryo-EM data processing was carried out using RELION v4.0.1^38^. The standard workflow applied to all samples is summarized in Supplementary Fig. 5, using the CspA:CspC:taurocholate complex as a representative example. Beam-induced motion correction and contrast transfer function (CTF) estimation were performed with MotionCor2^39^ and CTFFIND v4.1^40^, respectively. For initial particle selection, approximately 300 randomly selected micrographs were manually inspected and picked. After extensive two-dimensional (2D) classification, the resulting curated particle set was used to train the Topaz v0.2.5 neural network-based particle picker^41^. The trained Topaz model was subsequently employed for automated particle picking across the complete micrograph dataset. For the pro/CspB, and CspA:CspC:taurocholate complexes, 3D refinements were performed using particles from the selected 2D classes with a box size of 200 pixels for automatic high-resolution gold-standard refinement. CTF refinement and Bayesian polishing were subsequently applied, followed by one or two additional rounds of gold-standard refinement to generate the final reconstructions. Map resolution was determined using the 0.5 Fourier shell correlation (FSC) criterion, and data collection and processing statistics are summarized in Supplementary Table 3.

### Determination of helical symmetry parameters for CspB fibrils

A subset of 684 electron micrographs was used to manually pick 3,364 fibrils in RELION v4.0.1^42^. Fibril segments were initially extracted with 1.68 Å/pixel binning, using a placeholder helical rise value of 1 Å. After 2D classification, several selected class averages were analyzed with the Python-based Helix Indexer (PyHI v007) software^43^, which facilitates Fourier–Bessel indexing of power spectra for helical symmetry determination. Several alternative helical lattices were evaluated through preliminary 3D refinements in RELION v4.0.1, and visual inspection of the resulting maps identified the most consistent lattice, with a rise of 24.6 Å and a twist of –135°. These parameters were then applied for a new round of helical segment extraction at 0.839 Å/pixel, using a rise of 24 Å and one asymmetric unit per segment. This yielded 84,764 helical segments, which were subjected to two consecutive rounds of 2D classification, selecting the best averages and associated segments. Calculated parameters for several alternative lattices were tested in preliminary 3D refinements in RELION v4.0.1, and visual inspection of the calculated maps resulted in the selection of one of the lattices (rise = 24.6 Å and twist = -135°). These values were taken into account for a new extraction of helical segments at 0.839 Å/px using a rise value of 24 Å and one asymmetric unit per segment. The resulting 84,764 helical segments were subjected to two consecutive 2D classification rounds and selection of the best featured averages and related segments. Subsequently, 3D classification of the final 58,779 helical segments was performed in RELION v4.0.1, allowing for local searches to refine the helical parameters (rise in the range: 23–26 Å; twist in the range: 130–140°). The refined parameters converged to a rise of 24.7 Å and a twist of 135.9°, consistent with a right-handed helix (despite the initial negative twist values suggesting a right-handed symmetry), as the right-handed reconstruction clearly resolved densities corresponding to CspB dimers. These parameters were used to generate a preliminary 3D reconstruction of the CspB fibrils at 3.4 Å resolution. For the final reconstruction, 228,881 helical segments were extracted from the complete dataset, and the process described above was repeated using unbinned particles. Helical reconstruction with the refined rise and twist parameters yielded a 2.75 Å-resolution map of the pro/CspB oligomer.

### Atomic model building and refinement

For both the pro/CspB dimer and pro/CspB oligomer, the crystallographic structure determined in this study (PDB ID: 9SJ6) was used as the starting model. For the CspA:CspC:taurocholate complex, initial models were generated using AlphaFold3^44^. In all cases, the models were docked into their corresponding cryo-EM density maps as rigid bodies, followed by manual model adjustment in Coot v0.9.4.1^45^. Subsequently, the atomic models were refined against their respective maps using Phenix.real_space_refine v1.19.2-4158^46^.

### Reporting summary

Further information on research design is available in the Nature Portfolio Reporting Summary linked to this article.

## Supplementary information

Supplementary Information

Reporting Summary

Transparent Peer Review file

## Source data

Source data

## Data Availability

All data necessary to support the conclusions of this study are provided in the main text, including the main figures and Supplementary Information. Cryo-EM maps and atomic models presented in this study have been deposited into the Worldwide Protein Data Bank (wwPDB) and the Electron Microscopy Data Bank (EMDB) with accession codes PDB 9SJ6 for the crystallographic structure of pro/CspB, PDB 9SJ7 and EMD-54939 for pro/CspB, PDB 9SJB and EMD-54942 for pro/CspB oligomer, and PDB 9SJ9 and EMD-54941 for CspA:CspC:taurocholate complex, and PDB 9T7M for CspA:taurocholate complex. Real space refinement statistics are summarized in Supplementary Table 3. Particle angular distributions and representative cryo-EM and crystallographic electron density maps supporting the structural models presented in this study are provided in Supplementary Figs. 12–15. Links to the previously published structures used in the manuscript can be found at PDB 4I0W, PDB 6MW4. Source data are provided with this paper.
