# Supplementary material for: Structure of core assembly of the Clostridioides difficile germinosome
Source: Nat Commun. 2026 Jun 17;17:7669. doi: 10.1038/s41467-026-74264-w (PMC13434707; doi:10.1038/s41467-026-74264-w)
Supplement: Supplementary file 1 [file 41467_2026_74264_MOESM1_ESM.pdf]

Supplementary Information

Structure of Core Assembly of the *Clostridioides difficile* Germinosome

Carlos Cal y Mayor Luna[1,‡], Martín Alcorlo Pages[2,‡], Choon Kim[1], Giselle N. Jacobson[3], Melisa Lázaro[4], Mikel Valle[4], Mayland Chang[1*], Juan A. Hermoso[2*], and Shahriar Mobashery[1*]

[1]Department of Chemistry and Biochemistry, University of Notre Dame, Notre Dame, Indiana 46556, United States.

[2]Department of Crystallography and Structural Biology, Instituto de Química-Física "Blas Cabrera", Consejo Superior de Investigaciones Científicas, 28006-Madrid, Spain.

[3]Biophysics Instrumentation Core Facility, University of Notre Dame, Notre Dame, IN 46556, United States.

[4]Center for Cooperative Research in Biosciences (CIC bioGUNE), Basque Research and Technology Alliance (BRTA), Bizkaia Technology Park, Derio, Spain.

*Corresponding authors. Emails: mchang@nd.edu; xjuan@iqf.csic.es; mobashery@nd.edu

‡Both authors contributed equally to this work.

Supplemental Methods

Cloning Strategies and Csp Protein Production. To investigate interactions among CspA, CspB and CspC, the gene for each was cloned into *E. coli* expression vectors to produce recombinant proteins. The *cspA* gene amplified from the genomic DNA of the *C. difficile* 630 strain. The *cspA* gene and the synthetic *cspB* gene were cloned into pET28a-CPDsal and pET24a, respectively. The resulting plasmids were used for the generation of the plasmids producing the Twin-Strep-tagged variants of each protein. The plasmid producing the Twin-Strep-tagged CspA was created from pET22b-CspC-CPDsac[1].

The activated form of CspB (excluding the pro domain) precipitated as inclusion bodies when the gene was expressed in *E. coli*, as also observed by Adams *et al.*[2]. Therefore, we cloned the *cspB* gene, encoding full-length CspB, hereafter referred to as proCspB. This protein underwent partial auto-cleavage, which resulted in an inseparable mixture of the two forms (the full-length and the processed version). As we needed to work with homogeneous samples, we resorted to co-expressing the pro-domain and the mature form of CspB. To this end, a stop codon was inserted after the sequence encoding the pro-domain, and a new ribosome-binding site and an additional start codon were added before the sequence encoding the mature CspB. The construct allows for co-expression of the pro-domain and the mature form of CspB, which resulted in isolation of a clean product for the complex of the pro-domain bound to the mature CspB. This sample exhibited good solubility.

We also experienced solubility issues for full-length CspA and CspC. In order to enhance CspA solubility, we fused the cysteine protease domain (CPD) of *Vibrio cholerae* to the *C*-terminus of CspA and purified the resulting protein to homogeneity. After purification, CPD was cleaved in the presence of phytic acid[3,4]. Phytic acid activates the CPD for its autocleavage in this process. CspA was purified to homogeneity at this point. We procured the construct for CspC from Prof. Aimee Shen, which was also prepared as a fused protein with the CPD domain[1]. Furthermore, we produced all three Csp proteins fused with a Twin-Strep-tag (abbreviated as ST) either at the *N*-terminus or at the *C*-terminus for the use in surface-plasmon resonance (SPR) experiments. We successfully purify to homogeneity these Csp proteins: CspA, CspA-ST, CspC, ST-CspC, proCspB, ST-proCspB, and the complex of the pro-domain and the mature CspB (hereafter referred to as pro/CspB; Supplementary Fig. 1a).

YabG, a germination-specific protease, cleaves a single polypeptide CspBA in *C. difficile*, resulting in two proteins, proCspB and CspA. It also converts the zymogenic preproSleC to a smaller zymogenic proSleC during sporulation, and proSleC serves as a substrate for CspB during germination[5]. To produce pro-SleC from the purified preproSleC for the investigation of the *in vitro* proteolytic activity of pro/CspB, the full-length *yabG* gene was amplified from the genomic DNA of the *C. difficile* 630 strain and then was cloned into the expression vector pET24d. The production and the purification of recombinant proYabG proved challenging due to its unchecked autoproteolytic activity, as well as toxicity in *E. coli*, likely due to issues with heterologous expression. The proYabG production in *E. coli* led to premature autoprocessing of zymogenic proYabG (32 kDa) to the active form YabG (22 kDa). The self-degradation of YabG (10 kDa–15 kDa fragments) compelled us to work with freshly prepared samples. Addition of benzamidine to the buffers in the course of production of YabG helped in slowing down the auto-degradation process, as noted for the related *Bacillus subtillis* protein (Supplementary Fig. 1b)[6]. We identified the cleavage site of proYabG by *N*-terminal amino-acid sequencing at the amide bond flanked by R88 and A89.

Supplementary Table 1. Primers used in this study.

Name	Sequence (5' to 3')	Comment
CK287	CATATGGCCCTGAAAATAAAGATTC	Forward primer to amplify pST vector backbone form pST-proCspB6H
CK322	TAACTCGAGCTGAGATCCGGCTGCTAAC	Reverse primer to amplify pST vector backbone form pST-proCspB6H
CK421[a]	TATTACCATGGGATTTAAAGATGAGGAAAATAGTTATAAGTTTATAG	Forward primer of *cspA*
CK422[a]	AATAAGTCGACTCTTAAAACATCAAACATTCCTCTAAC	Reverse primer of *cspA*
CK423[a]	AATAACCATGGGTAAGGTAGGAGACATTGTAGC	Forward primer of *yabG*
CK424[a]	AATAACTCGAGATGTAATATTGTTTTTGGCATTGC	Reverse primer of *yabG*
CK457[b]	GAGATATACC**ATG**CAAGATGTGCAGAGC	Forward primer for pET24a-pro/CspB6H; KLD cloning
CK458[b]	CTTCTTAAAG**TTA**GGTCTGCAGAATAAACG	Reverse primer for pET24a-pro/CspB6H; KLD cloning
CK492	GGCAGCGCATGGAGTC	Forward primer for deletion of the *N*-terminal 6×His-tag in pETHST-proCspB6H
CK493	GCTGCCCATGGTATATCTCC	Reverse primer for deletion of the *N*-terminal 6×His-tag in pETHST-proCspB6H
CK503[b]	**TAA**CTTTAAGAAGGAGATATACCATGC	Forward primer for deleting the C-terminus of the Pro-domain of CspB; KLD cloning
CK504	AATAAACGGTTTTTCGATGAACTC	Reverse primer for pET24a-pro3D/CspB6H; KLD cloning

CK505	TTTTTCGATGAACTCAATTTCCG	Reverse primer for pET24a-pro6D/CspB6H; KLD cloning
CK506	GAACTCAATTTCCGGATAGG	Reverse primer for pET24a-pro9D/CspB6H; KLD cloning
CCL_13	CTTTATTTTCAGGGCCATATGGGATTTAAAGATGAGGAAAATAG	Forward primer to amplify *cspA-cpd* from pET28a-CspA-CPD6H
CCL_14	GGATCTCAGCTCGAGTTAACCTTGCGCGTCCCAG	Reverse primer to amplify *cspA-cpd* from pET28a-CspA-CPD6H
CCL_15	TGGTGCTCGAGACCTTGCGCGTCCCAG	Forward primer to add 6xHis-tag to ST-CspA-CPD
CCL_16	CCACCACCACCACTGAGATCCGGCTGCTAAC	Reverse primer to add 6xHis-tag to ST-CspA-CPD
CCL_22	GGTGGATCGGGAGGTTCAGCGTGGAGCCACCCGCAGTTCGAAAAATCTTTAGCGGATGGAAAAATACTCC	Forward primer to relocate ST tag to the *C*-terminus of CspA using vector ST-CspA-CPD6H
CCL_23	GCCAGAACCTCCACCTTTCTCGAATTGAGGATGACTCCATGCGCTGCCTCTTAAAACATCAAACATTCCTCTAAC	Reverse primer to relocate ST tag to the *C*-terminus of CspA using vector ST-CspA-CPD6H

[a]Restriction sites are underlined: NcoI (CCATGG), SalI (GTCGAC) and XhoI (CTCGAG).

[b]Start and stop codons are in bold.

Supplementary Table 2. Crystallographic data collection and refinement statistics*

	Pro:CspB	CspA:TAU
**Data collection**		
Wavelength (Å)	0.97926	0.97918
Space group	C 2 2 2_1	P 2 2_1 2_1
Unit cell *a, b, c* (Å)	101.91 115.73 201.69	141.24 158.63 188.36
Unit cell α,β,γ (º)	90, 90, 90	90, 90, 90
T (K)	100	100
X-ray source	Synchrotron	Synchrotron
Resolution range (Å)	49.42–1.94	49.52–2.90
	(1.97–1.94)	(2.95–2.90)
Unique reflections	88232 (4488)	94280 (4617)
Completeness (%)	100. 0 (100.0)	100. 0 (100.0)
Multiplicity	13.4 (13.7)	13.4 (13.9)
$R_{merge}{}^{a}$	0.086 (1.118)	0.196 (1.125)
$R_{pim}{}^{b}$	0.024 (0.312)	0.055 (0.312)
$\langle I/\sigma(I) \rangle$	18.8 (2.7)	14.2 (2.6)
CC1/2	0.999 (0.888)	0.996 (0.812)
**Refinement**		
Resolution range (Å)	49.40–1.94	49.50–2.90
$R_{work}/R_{free}{}^{c}$	0.2187/ 0.2578	0.2049/ 0.2406
No. Atoms		
Protein	8270	25211
Water	265	-
Ligand	-	280
**R.m.s. deviations**		
Bond length (Å)	0.008	0.003

Bond angles (°)	1.01	0.58
**Ramachandran**		
Favored/outliers (%)	97.34/0.00	97.81/0.03
Monomers per AU	2	6
**Average B-factor**	61.14	56.30
Macromolecules	61.56	56.18
Ligands	-	67.26
Solvent	47.81	-
**PDB code**	9SJ6	9T7M

*Values between parentheses correspond to the highest resolution shells

[a] $R_{merge} = \Sigma_{hkl} \Sigma_i | I_i(hkl) - [I(hkl)] | / \Sigma_{hkl}\Sigma_i I_i(hkl)$, where $\Sigma_i I_i(hkl)$ is the *i*-th measurement of reflection hkl, [I(hkl)] is the weighted mean of all measurements.
[b] $R_{pim} = \Sigma_{hkl}[1/(N-1)]^{1/2} \Sigma_i | I_i(hkl) - [I(hkl)] | / \Sigma_{hkl}\Sigma_i I_i(hkl)$, where $\Sigma_i I_i(hkl)$ is the *i*-th measurement of reflection hkl, [I(hkl)] is the weighted mean of all measurements and N is the redundancy for the hkl reflection.
[c] $R_{work}/R_{free} = \Sigma_{hkl}| F_o - F_c | / \Sigma_{hkl} | F_o |$, where F_c is the calculated and F_o is the observed structure factor amplitude of reflection hkl for the working / free (5%) set, respectively.

Supplementary Table 3. Cryo-EM data collection, refinement and validation statistics

Sample	Pro/CspB dimer	Pro/CspB fibril	CspA:CspC:TAU
**Data collection**			
Magnification	x105.000	x105.000	x105.000
Voltage (kV)	300	300	300
Electron exposure ($e^-/Å^2$)	41.2	41.2	42
Defocus range (μm)	1.0-2.0	1.0-2.0	0.8-2.0
Pixel size (Å)	0.839	0.839	0.840
Symmetry imposed	C_1	Helical symmetry (twist 135º; rise 24 Å)	C_1
Initial particles (nº)	5.515.141	234.881	9.346.409
Final particles (nº)	913.189	228.133	1.05.013
**Composition**			
Chains	4	34	2
Atoms	8270 (Hydrogens: 0)	67127 (Hydrogens: 0)	8635 (Hydrogens: 0)
Residues	Protein: 1062	Protein: 8615	Protein: 1102
Water	0	0	0
Ligands	0	0	TCH: 3
**Bonds (RMSD)**			
Length (Å) (# > 4σ)	0.005 (0)	0.003 (0)	0.003 (0)
Angles (º) (# > 4σ)	0.521 (1)	0.624 (24)	0.577 (7)
**MolProbity score**	1.62	1.68	1.14
**Clash score**	5.90	6.41	3.50
**Ramachandran plot (%)**			
Outliers	0.00	0.00	0.00
Allowed	1.52	2.17	1.37
Favored	98.48	97.83	98.63
**Rama-Z (Ramachandran plot Z-score, RMSD)**			
whole (N = 1054)	0.53 (0.26)	-0.19 (0.09)	0.28 (0.25
helix (N = 242)	0.87 (0.34)	-0.33 (0.12)	1.26 (0.35)
sheet (N = 254)	0.34 (0.33)	0.55 (0.11)	0.40 (0.30)
loop (N = 558)	0.27 (0.26)	-0.28 (0.09)	-0.31 (0.25)
**Rotamer outliers (%)**	2.47	2.40	0.43
Cβ outliers (%)	NA	NA	NA
**Peptide plane (%)**			
Cis proline/general	14.3/0.0	14.1/0.0	23.8/0.0
Twisted proline/general	0.0/0.0	0.0/0.0	0.0/0.0
**CaBLAM outliers (%)**	1.91	1.95	1.65
**ADP (B-factors)**			
Iso/Aniso (#)	8270/0	67127/0	8635/0
min/max/mean			
Protein	54.43/156.64/94.83	35.54/217.51/85.36	32.58/151.82/71.98
Ligand	---	---	54.35/220.03/93.06

**Occupancy**						
Mean	1.00		1.00		1.00	
occ = 1 (%)	100.00		100.00		100.00	
0 < occ < 1 (%)	0.00		0.00		0.00	
occ > 1 (%)	0.00		0.00		0.00	
**Data**						
**Box**						
Lengths (Å)	88.10, 97.32, 85.58		144.31, 144.31, 252.54		77.28, 93.24, 94.92	
Angles (°)	90.00, 90.00, 90.00		90.00, 90.00, 90.00		90.00, 90.00, 90.00	
**Map Resolution (Å)**	3.2		2.8		2.8	
**Resolution Estimates (Å)**	Masked	Unmasked	Masked	Unmasked	Masked	Unmasked
d 99 (full)	3.5	3.4	3.1	2.9	3.1	3.0
d model	3.4	3.4	3.0	3.1	2.9	2.9
d FSC model (0/0.143/0.5)	2.6/2.8/3.2	2.7/2.9/3.3	2.3/2.4/3.0	2.3/2.6/3.1	2.2/2.4/2.8	2.2/2.4/2.9
Map min/max/mean	-0.02/0.03/0.00		-0.03/0.05/0.00		-0.02/0.05/0.00	
**Model *vs* Data**						
**CC (mask)**	0.90		0.88		0.93	
**CC (box)**	0.81		0.69		0.83	
**CC (peaks)**	0.78		0.63		0.82	
**CC (volume)**	0.89		0.86		0.92	
**Mean CC for ligands**	---		---		0.85	
**PDB**	9ST7		9SJB		9SJ9	
**EMD**	54939		54940		54941	

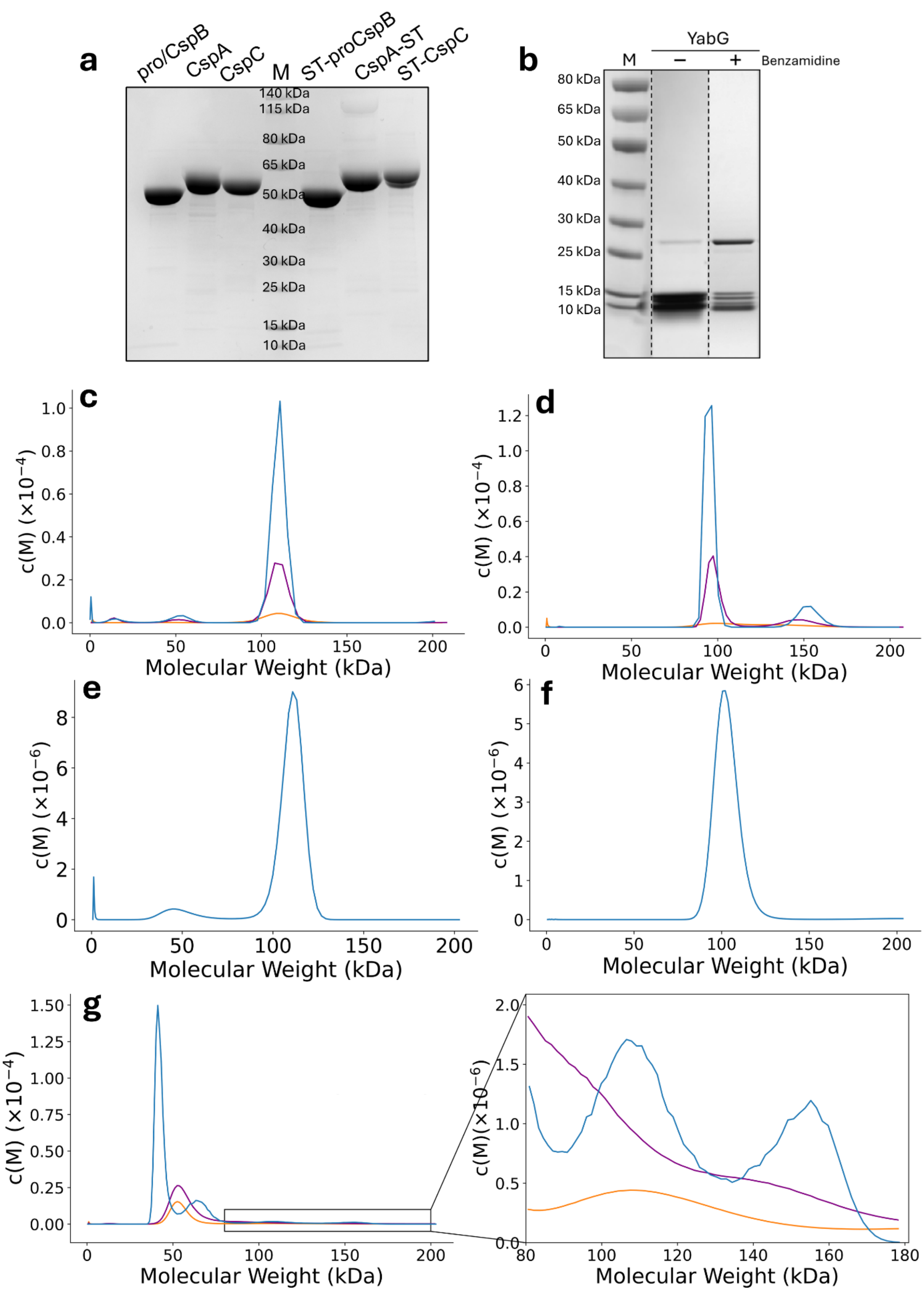
a
pro/CspB
CspA
CspC
M
ST-proCspB
CspA-ST
ST-CspC
140 kDa
115 kDa
80 kDa
65 kDa
50 kDa
40 kDa
30 kDa
25 kDa
15 kDa
10 kDa
b
YabG
M
−
+
Benzamidine
80 kDa
65 kDa
50 kDa
40 kDa
30 kDa
25 kDa
15 kDa
10 kDa
c
d
e
f
g
c(M) (×10⁻⁴)
c(M) (×10⁻⁶)
c(M)(×10⁻⁶)
Molecular Weight (kDa)

Supplementary Fig. 1 | Purified proteins used in this work and their oligomeric state in solution. a SDS-PAGE of purified Csp proteins. M is the molecular-weight marker. **b** Purified YabG in absence and in presence of 2 mM benzamidine. M is the molecular-weight marker. Dashed lines indicate that each lane in the image was cropped and grouped from the same gel (M and –) and a different gel (+). **c-g** Oligomeric states of Csp proteins analyzed by sedimentation velocity analytical ultracentrifugation (SV-AUC), monitored at 280 nm and 230 nm**.** Plots show the molar mass distribution (c(M)). **c** CspA monitored at 280 nm across a concentration range of 1.3–12.6 µM. CspA displayed a single peak at 110 kDa, corresponding to a dimer. **d** pro/CspB monitored at 280 nm in the range of 1.5–17.8 µM displayed a predominant peak at 97 kDa corresponds to a dimer, and a minor peak at 154 kDa corresponding to a trimer. **e** CspA monitored at 230 nm at 200 nM. A single peak at 110 kDa was observed, consistent with a dimer. **f** pro/CspB monitored at 230 nm at 180 nM. A single peak at 100 kDa was observed, consistent with a dimer. **g** CspC monitored at 280 nm in the concentration range of 1.9–13 µM. Peaks corresponding to monomeric, dimeric, trimeric, and tetrameric species were observed. Trimer and tetramer peaks are highlighted in the expanded view to the right. Due to the polydispersity of CspC, the mathematical treatment led to an underestimation of the apparent molecular weights.

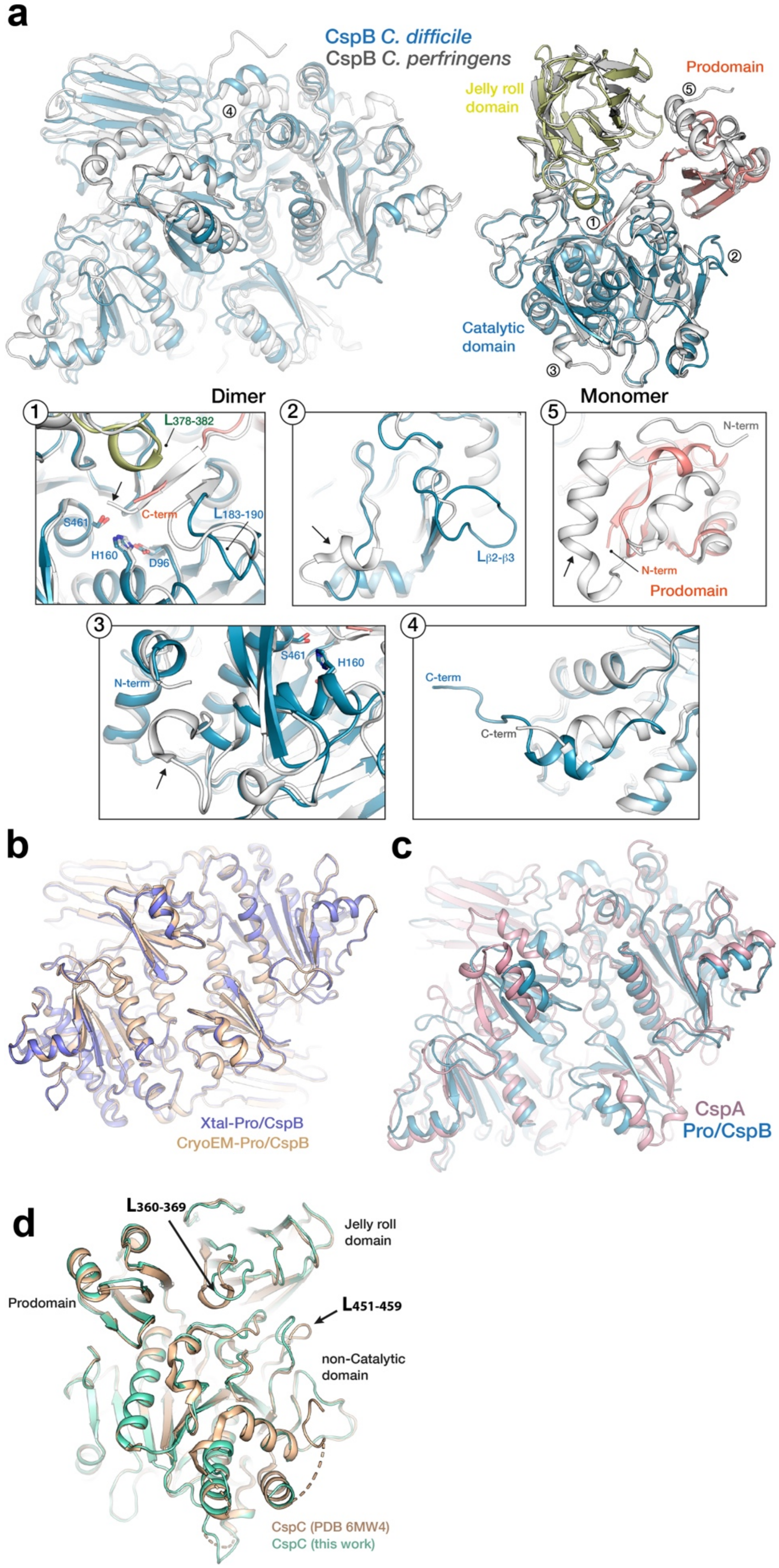
a
CspB C. difficile
CspB C. perfringens
Jelly roll domain
Prodomain
Catalytic domain
Dimer
Monomer
L378-382
S461
C-term
L183-190
H160
D96
Lβ2-β3
N-term
N-term
Prodomain
N-term
S461
H160
C-term
C-term
b
Xtal-Pro/CspB
CryoEM-Pro/CspB
c
CspA
Pro/CspB
d
L360-369
Jelly roll domain
Prodomain
L451-459
non-Catalytic domain
CspC (PDB 6MW4)
CspC (this work)

Supplementary Fig. 2 | a Structural comparison of CspB of *C. difficile* and *C. perfringens*. Left, structural superimposition of the crystal structure of pro/CspB dimer of *C. difficile* (blue cartoon, this work) onto the pro/CspB dimer of *C. perfringens* (gray cartoon, PDB code 4I0W) is given. Right, monomer superimposition of CspB of *C. difficile*, with each domain differently colored (catalytic in blue, jelly roll in green and prodomain in coral), and of CspB of *C. perfringens* (gray cartoon) is shown. Main differences (numbered onto the monomer and dimer images) are highlighted in the boxed areas: (1) active-site comparison shows the conservation of the catalytic triad, but the *N*-terminus of the *C. perfringens* CspB is longer (indicated by an arrow) than in the *C. difficile* structure and fully blocks the active-site entry; the jelly-roll domain in *C. difficile* CspB exhibits a small α-helix, shaping the active-site cavity. (2) The catalytic domain of *C. difficile* presents a very long loop between β2 and β3 (labelled). Also, *C. perfringens* has a small α-helix (indicated by an arrow). (3) An insertion is observed in the *C. perfringens* enzyme (arrow). (4) The C-terminus in the *C. difficile* enzyme is much longer than in *C. perfringens*; in *C. difficile* this region connects one monomer to the other in the dimer. (5) Differences in the prodomains are shown, with the *C. perfringens* enzyme being longer with an extra α-helix (indicated by an arrow). **b** X-ray crystal (blue cartoon) and cryo-EM (wheat cartoon) structures of pro/CspB of *C. difficile*. **c** Structural superimposition of pro/CspB and CspA of *C. difficile* homodimers is shown. **d** Superimposition of the crystal structure of CspC (PDB 6MW4, wheat cartoon) and CspC (this work, green cartoon) as observed in the CspA:CspC heterodimer, with main differences indicated by arrows.

The prodomain of CspB is Essential for the Stability of the Mature CspB. The prodomain of CspB enhances the solubility of the mature CspB during the production of CspB. It is noncovalently attached to the mature CspB after auto-processing during sporulation[2]. Based on the X-ray structures of pro/CspB from *C. difficile* (Fig. 3a) and that from *Clostridium perfringens*[2], we assume that the prodomain regulates the protease activity of CspB. The *C*-terminal region of the prodomain interacts directly with the active site in the catalytic domain of CspB for this function. In order to examine the effect of the *C*-terminus of the prodomain, we deleted amino acids from the *C*-terminus by three amino acids (Δ3), by six amino acids (Δ6) and by nine amino acids (Δ9). This resulted in constructs proΔ3/CspB, proΔ6/CspB and proΔ9/CspB, respectively. YabG converts CspBA to CspB and CspA, and preproSleC to proSleC. We incubated each variant of pro/CspB with the partially purified YabG (Supplementary Fig. 1b) to determine the effect of prodomain truncation on the stability of CspB. Surprisingly, we observed that proΔ9/CspB was completely and proΔ6/CspB was substantially degraded by YabG (Supplementary Fig. 3). Full-length pro/CspB and proΔ3/CspB were resistant to the activity of YabG.

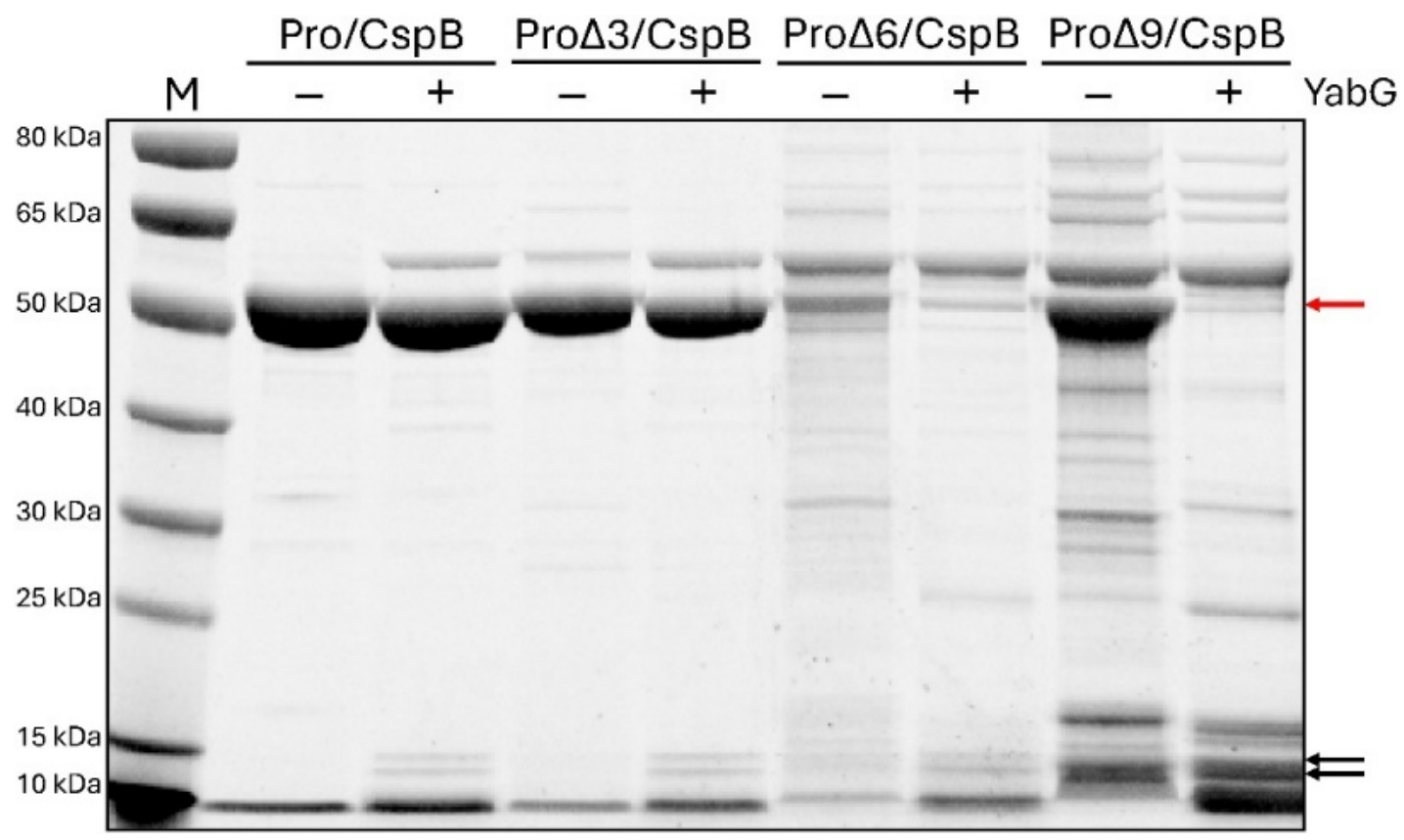

Supplementary Fig. 3 | Effect of the prodomain *C*-terminus on the stability of CspB. The variants of pro/CspB were incubated with partially purified YabG at 32 °C for 3 hours. The red arrow and the black arrows indicate the variant of pro/CspB and the degraded-but-active YabG, respectively. M is a molecular-weight marker.

Next, we examined the protease activity of the pro/CspB variants. Wild-type pro/CspB did not show protease activity as expected. Interestingly, proΔ3/CspB marginally converted proSleC—produced from preproSleC by pre-incubation with YabG—to SleC only in the presence of 1 mM $ZnCl_2$ (Supplementary Fig. 4) In contrast, a Zn^{2+} ion was not observed either in the X-ray structure or in cryo-EM structure of pro/CspB, although the structure of proΔ3/CspB has not been resolved. The presence of 1 mM $CaCl_2$, 10 mM sodium taurocholate, CspA or CspC did not increase the activation level of proΔ3/CspB by $ZnCl_2$. Calcium ion has been proposed to be a co-germinant previously[7].

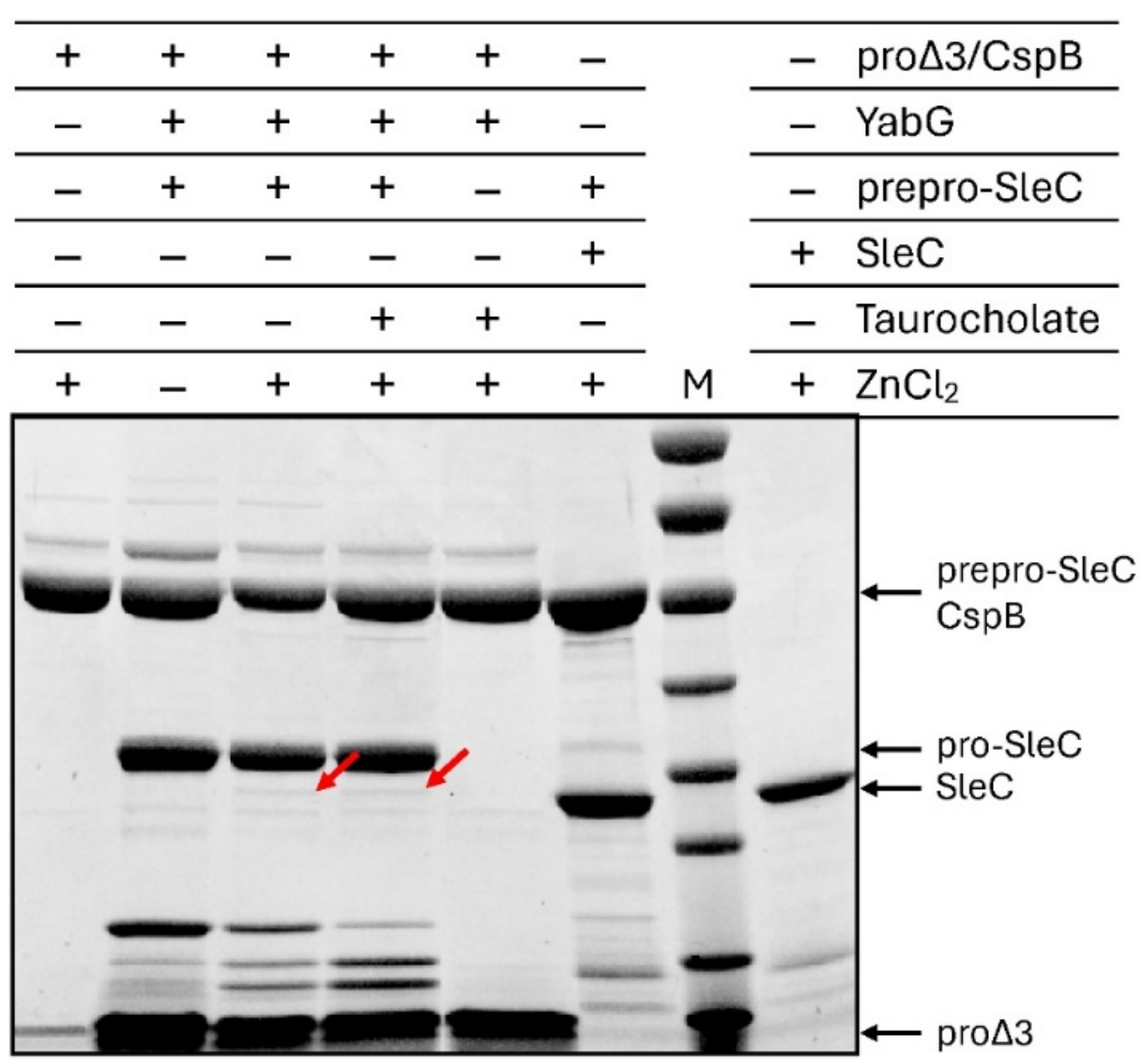

Supplementary Fig. 4 | Requirement of $ZnCl_2$ for the activation of proΔ3/CspB. proΔ3/CspB exhibited turnover of proSleC as its substrate only in the presence of 1 mM $ZnCl_2$. The activation of proΔ3/CspB is independent of sodium taurocholate (10 mM), CspA and CspC. The red arrows indicate SleC produced by proΔ3/CspB.

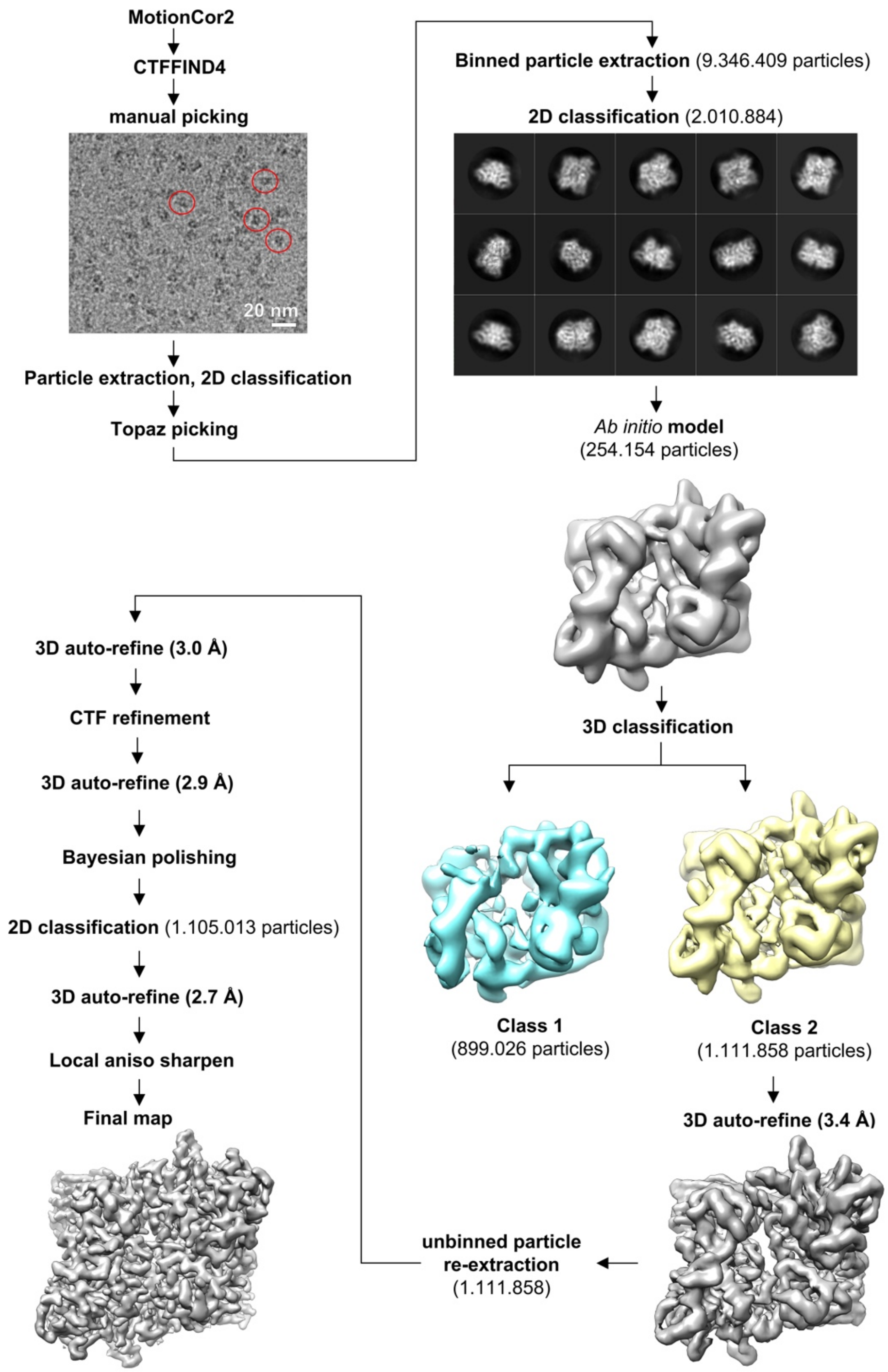

Supplementary Fig. 5 | Processing scheme for CspA:CspC:taurocholate complex. The different steps involved in the cryo-EM data processing workflow are indicated. A representative micrograph as well as some examples of 2D classes and major classes form 3D-reconstruction are shown. The presented workflow was also applied to pro/CspB. See methods for details.

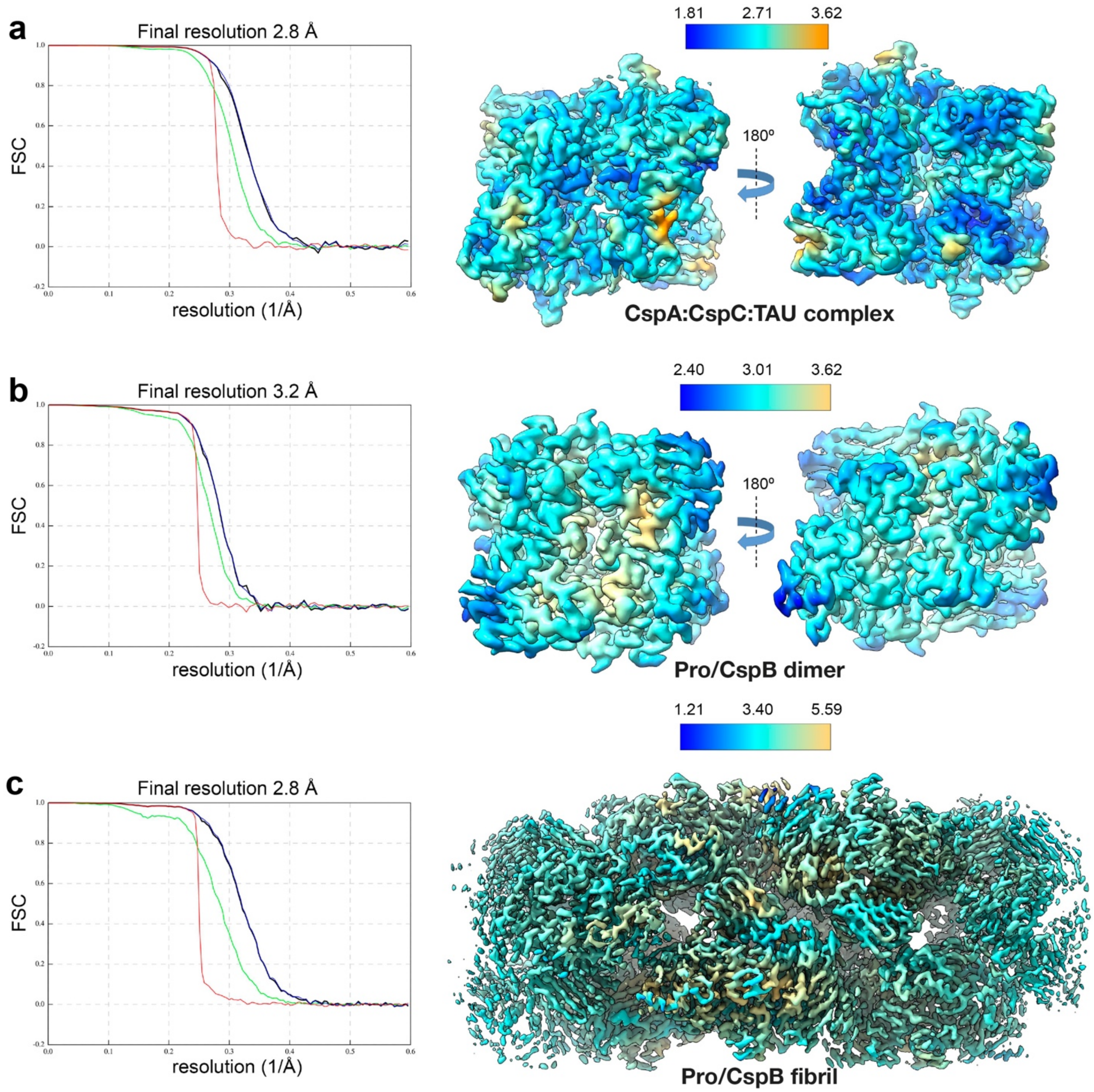

Supplementary Fig. 6 | Global and local resolution of the cryo-EM structures presented in this work. a CspA:CspC:TAU complex. **b** Pro/CspB dimer. **c** Pro/CspB fibril. In the four cases, the left panel depicts the Fourier Shell Correlation (FSC) curves, while the middle and right panels display the reconstruction colored according to local resolution. The two views are oriented 180° apart. In all FCS graphs, the black line shows the FSC for the masked map after correction for masking effects. The red curve represents the outcome obtained when the map phases are randomized. The FSC between the two unmasked half-maps is shown in green. The FSC between the two half-maps to which the solvent mask has been applied is shown in blue.

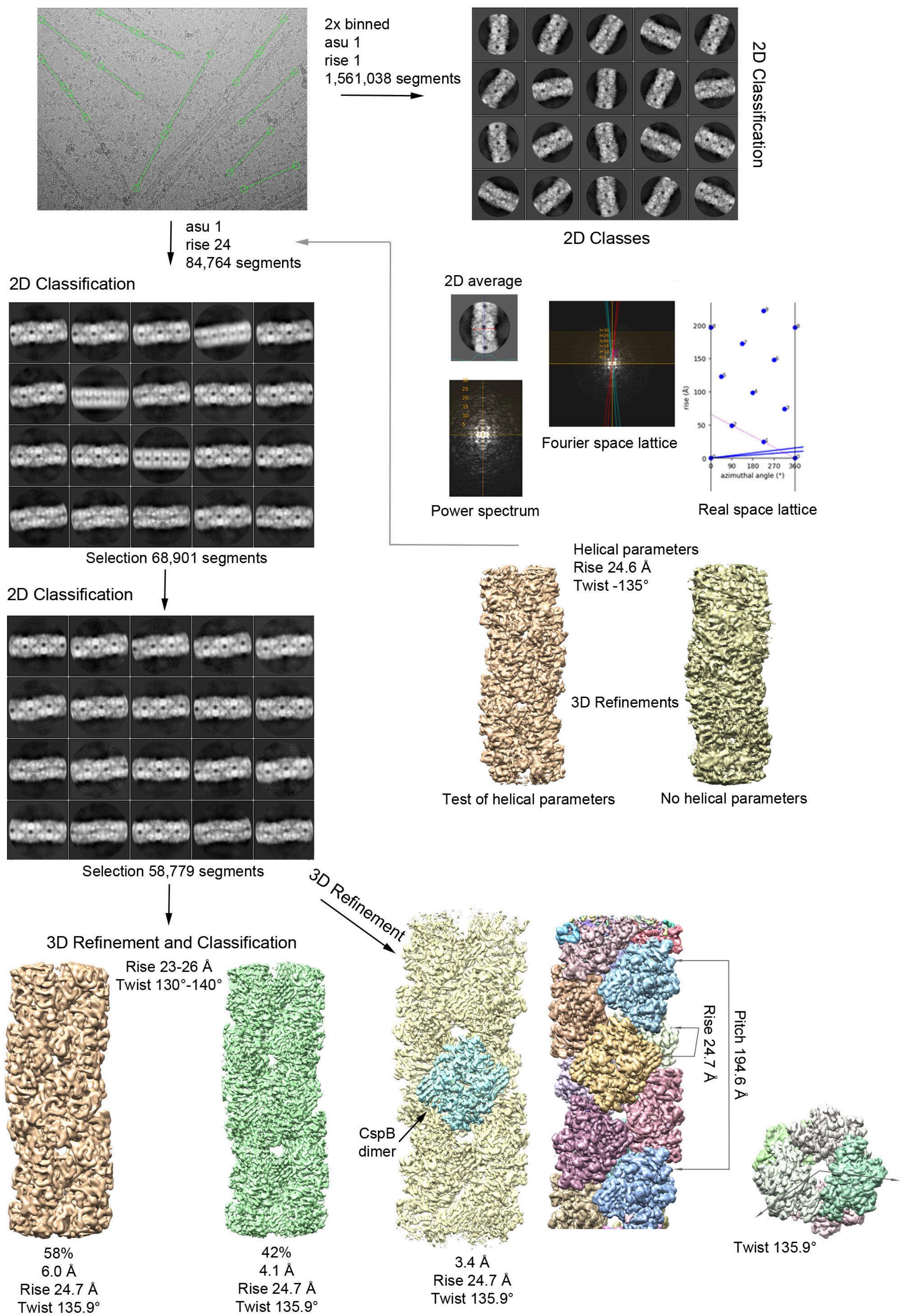
2x binned
asu 1
rise 1
1,561,038 segments
2D Classification
2D Classes
asu 1
rise 24
84,764 segments
2D Classification
2D average
Power spectrum
Fourier space lattice
Real space lattice
azimuthal angle (°)
rise (Å)
Selection 68,901 segments
Helical parameters
Rise 24.6 Å
Twist -135°
3D Refinements
Test of helical parameters
No helical parameters
2D Classification
Selection 58,779 segments
3D Refinement
3D Refinement and Classification
Rise 23-26 Å
Twist 130°-140°
CspB
dimer
Rise 24.7 Å
Pitch 194.6 Å
58%
6.0 Å
Rise 24.7 Å
Twist 135.9°
42%
4.1 Å
Rise 24.7 Å
Twist 135.9°
3.4 Å
Rise 24.7 Å
Twist 135.9°
Twist 135.9°

Supplementary Fig. 7 | Single particle cryo-EM analysis of the pro/CspB fibrils. Schematic representation of the strategy to define the helical parameters for thr pro/CspB fibrils (see Methods). Briefly, an initial extraction of segments was subjected to 2D classification in RELION, and the power spectra of the resulting averages were analyzed by PyHI software to provide parameters of potential lattices. These parameters were used in 3D reconstructions for visual inspection of the 3D maps and selection of the volumes with best-defined densities (rise=24.6 Å and twist=-135°). A new extraction of helical segments was guided by these parameters, and two sequential 2D classifications lead to a final subset of 58,779 segments. Here, both, 3D classification into two classes, and 3D refinement (both in RELION) led to identical symmetry parameters (rise=24.7 Å and twist=135.9°) and only the use of the positive twist value (right-handed helix) delivered maps with evident signal for the presence of pro/CspB dimers.

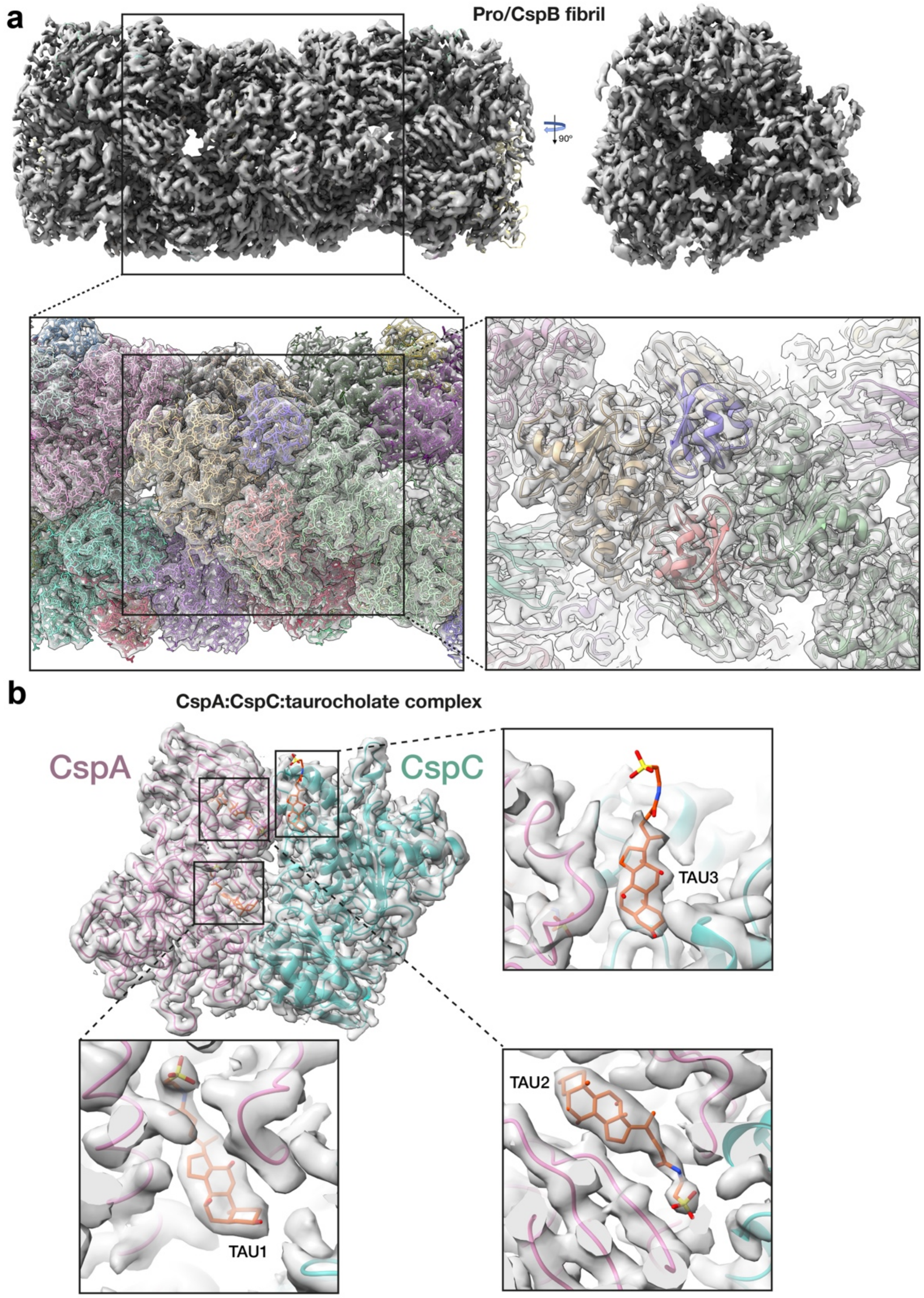

Supplementary Fig. 8 | Details of the single-particle cryo-EM density for the pro/CspB fibrils, and CspA:CspC:taurocholate complexes. **a** Two perspectives of the fibril are show, one lengthwise and the other down the axis of the fibril superhelix. Two zoomed-in views of the fibril are given as well (colored according to the domains). **b** Cryo-EM density for the CspA:CspC:taurocholate complex and for the TAU molecules are given.

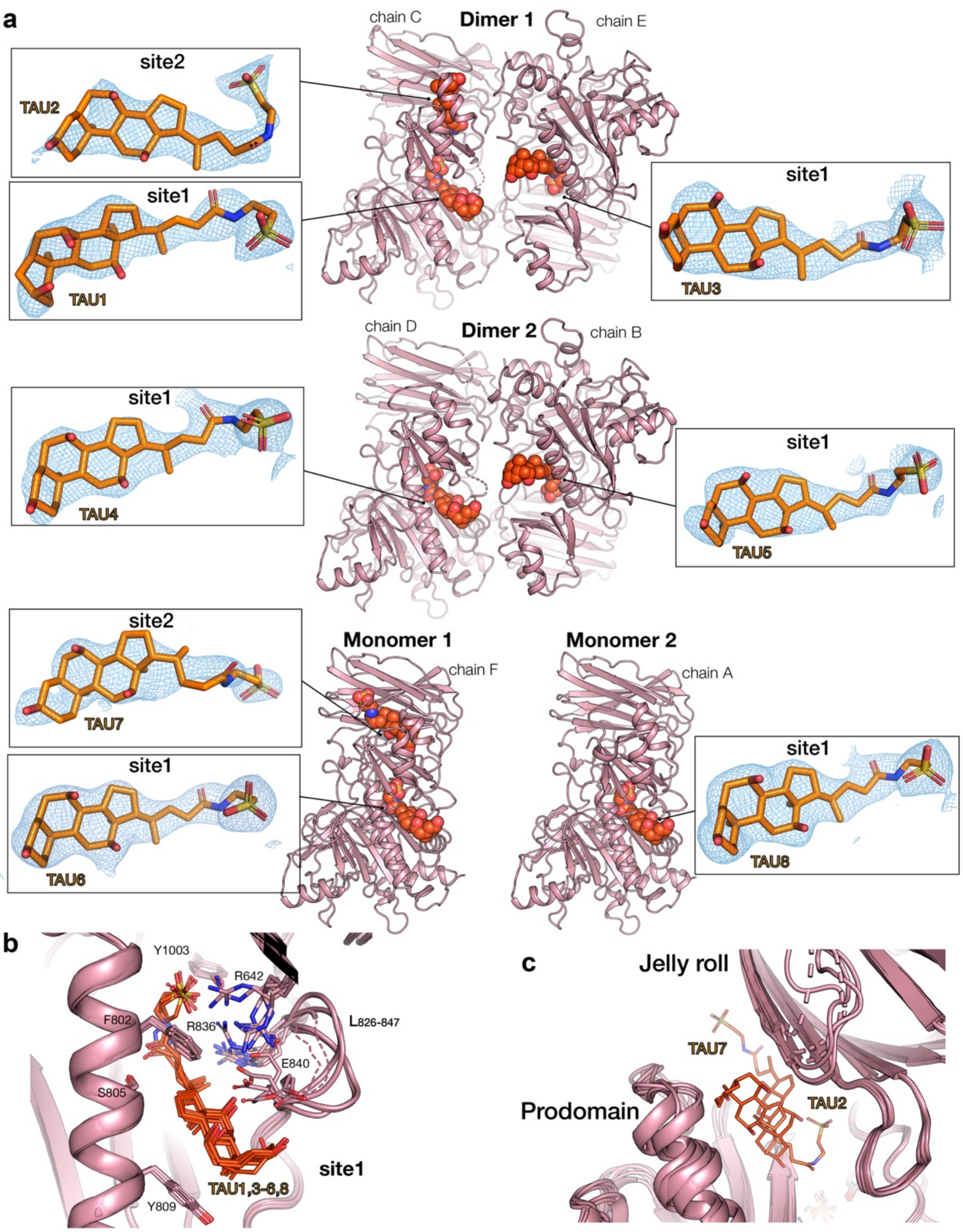

Supplementary Fig. 9 | Taurocholate-binding sites in the crystal structure of CspA:taurocholate complex. **a** The six independent CspA chains are represented in cartoon with the associated TAU molecules in spheres. Boxes present the 2Fo-Fc electron density maps contoured at 1 σ for the 8 TAU molecules. **b** Detailed view of the Site 1 as observed in the six CspA independent chains (superimposed). TAU molecules are represented as capped sticks and labelled. Relevant residues in TAU stabilization are represented as sticks. **c** Detailed view of the Site 2 as observed in the six CspA independent chains (superimposed). TAU molecules are represented as capped sticks and labelled.

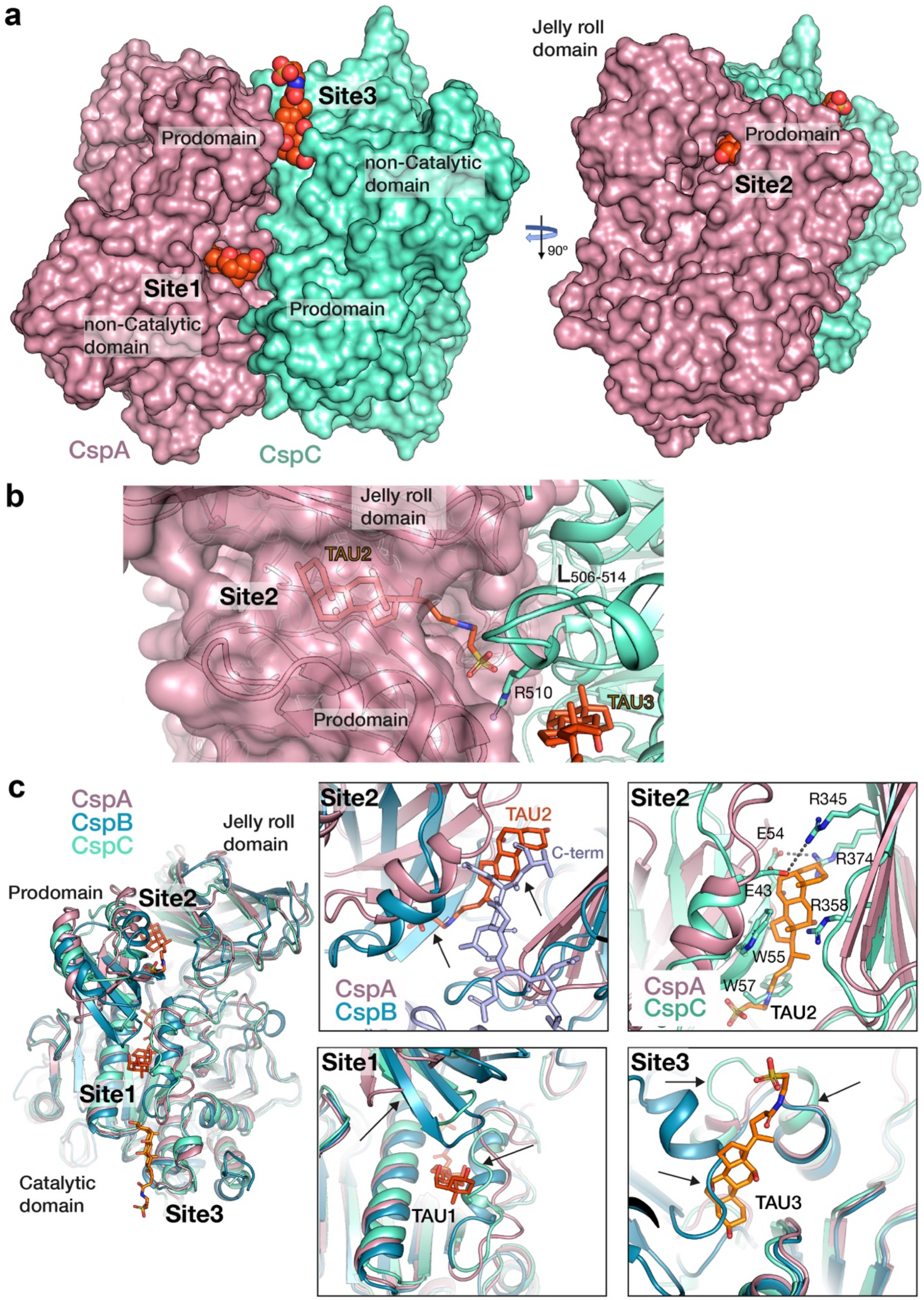

a
Jelly roll domain
Site3
Prodomain
non-Catalytic domain
Site2
90°
Site1
Prodomain
non-Catalytic domain
CspA
CspC
b
Jelly roll domain
TAU2
Site2
L506-514
R510
TAU3
Prodomain
c
CspA
CspB
CspC
Jelly roll domain
Prodomain
Site2
Site1
Catalytic domain
Site3
TAU2
C-term
R345
E54
R374
E43
R358
W55
W57
TAU2
TAU1
TAU3

Supplementary Fig. 10 | Taurocholate-binding sites in the CspA:CspC heterodimer and comparison among the three Csp proteins. **a** Two views showing the three sites with taurocholate molecules represented as spheres. **b** Detailed view of the Site 2 of CspA:CspC complex is given. CspA is represented as a transparent molecular surface and colored in pink, and CspC is represented as a green cartoon. Taurocholate molecules TAU2 and TAU3 are represented as orange sticks. R510, important in the recognition of TAU2, is represented as capped sticks. **c** Structural superimposition of CspA (pink cartoon), CspB (blue cartoon) and CspC (green cartoon) is given with the three taurocholate molecules (TAU1, TAU2 and TAU3) represented as orange sticks. Boxes represent zoomed-in views of the three sites comparing CspA, CspB and CspC. Relevant differences are highlighted by arrows. Comparison of Site 2 in CspA and in CspB (upper left box) and in CspA and in CspC (upper right box) are detailed. In CspC there are strong interactions linking the jelly-roll domain with the prodomain, that, together with the presence of bulky amino acids, block the accommodation of taurocholate at Site 2. Relevant residues are represented as capped sticks and labelled.

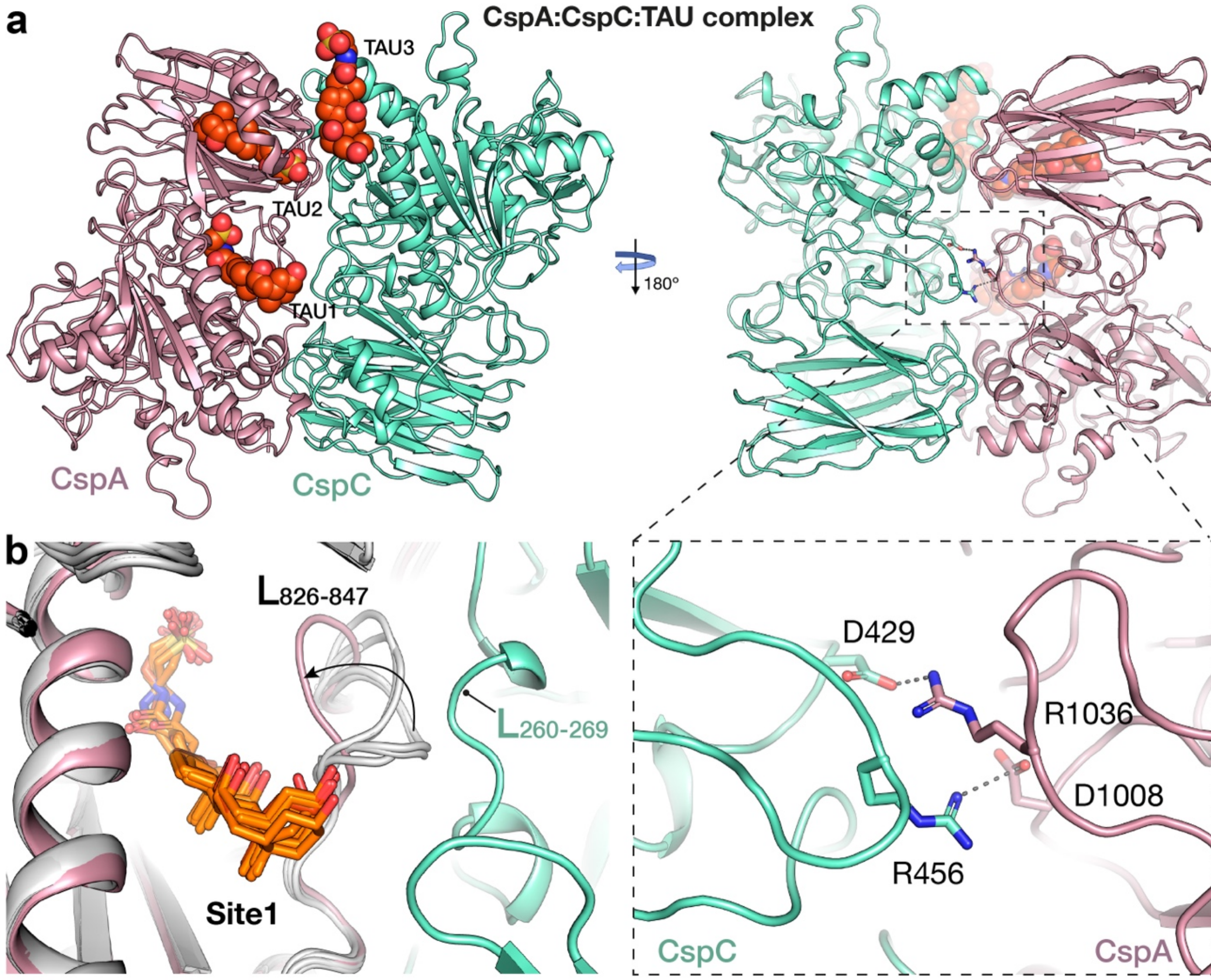

Supplementary Fig. 11 | a Electrostatic interactions linking the CspA:CspC dimer. Interactions depicted as dashed lines with the main residues represented as capped sticks. **b** Superposition of Site 1 in all the CspA chains of the CspA:TAU complex (white cartoon) onto the CspA:CspC:TAU complex (CspA coloured in magenta, and CspC colored in green). Relevant regions labelled. The change in the conformation of the CspA loop $L_{826-847}$ from the monomer, homodimer to the heterodimer is represented by an arrow. TAU molecules are represented as orange capped sticks.

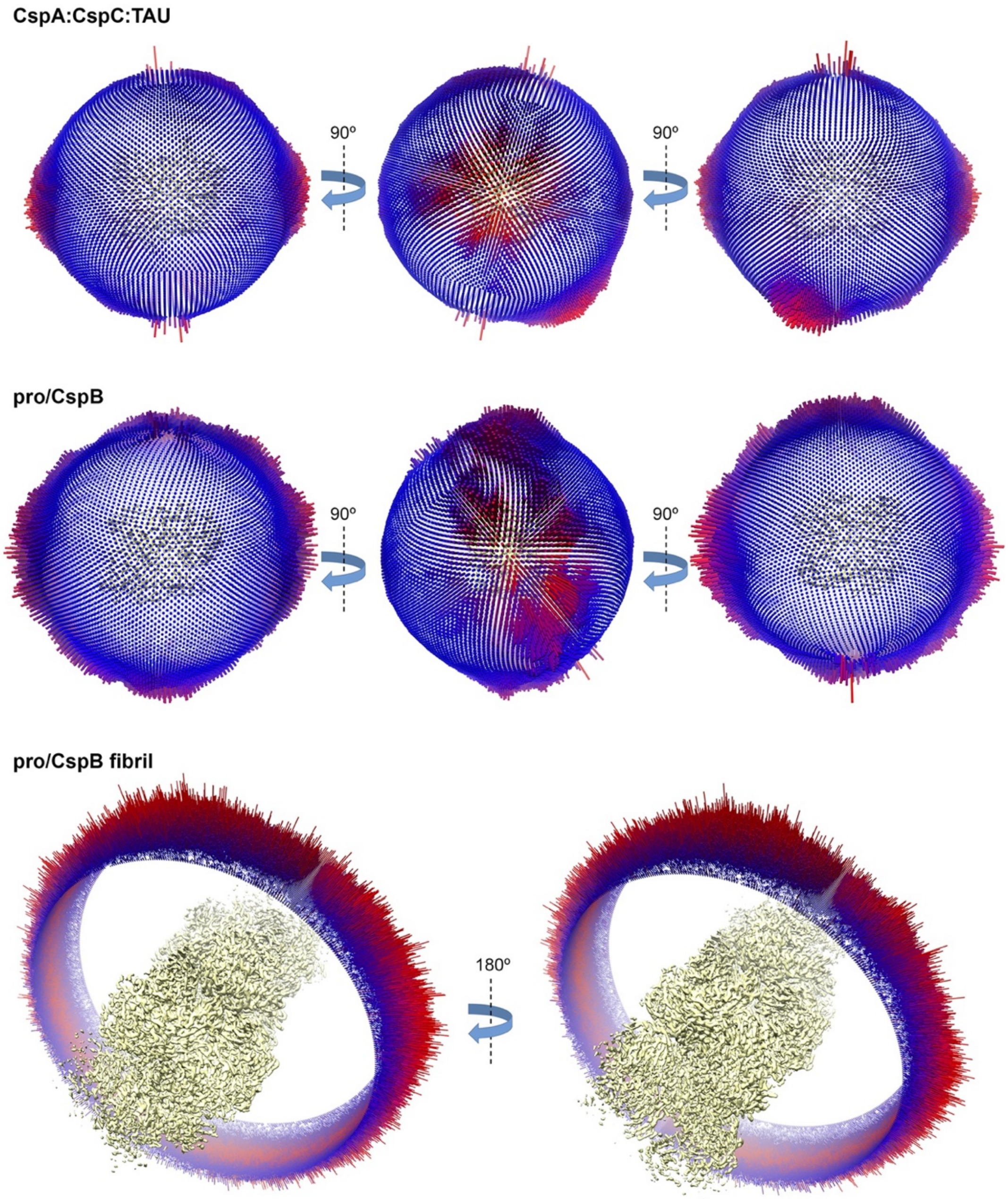

Supplementary Fig. 12 | Angular distribution plots of the particle images contributing to the final cryo-EM. The angular distributions are represented as cylindrical projections surrounding each reconstructed map, where the cylinder length is proportional to the number of particles assigned to a given orientation. The color gradient ranges from blue to red, indicating increasing particle density for the corresponding viewing direction. In the center of the plot, the indicated cryo-EM map is displayed as yellow colored surface. Rotated views are shown to highlight the orientation coverage of the datasets. Reconstructions generated in RELION and visualized in UCSF Chimera.

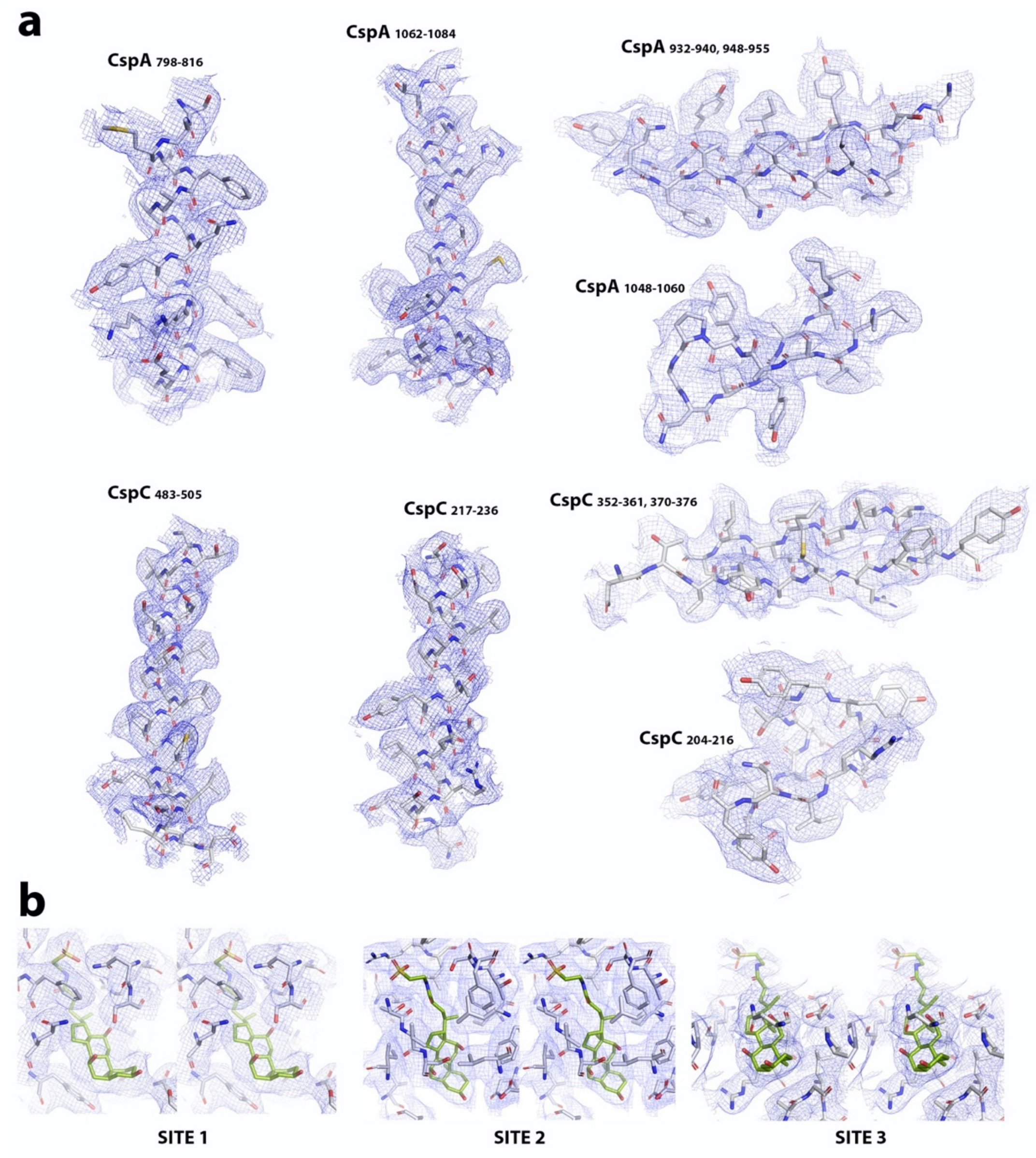

Supplementary Fig. 13| Structural features and ligand density of the CspA:CspC:taurocholate complex resolved by cryo-EM. a Representative cryo-EM electron-density maps for selected regions of CspA and CspC, highlighting the quality of the reconstruction and the agreement between the density and the refined atomic model. Residue ranges corresponding to each displayed region are indicated above the maps. The cryo-EM density is shown as a blue mesh surrounding the refined model (at a threshold level of 4.6σ). **b** Stereo wall-eye views of the cryo-EM density corresponding to taurocholate bound at sites 1, 2, and 3 within the CspA:CspC complex. The ligand is represented as green sticks, while the surrounding protein residues are shown as gray sticks. The cryo-EM density map is displayed as a blue mesh contoured around the ligand, supporting the assignment and orientation of taurocholate at each binding site. Volume and structural representations were prepared using PyMol 3.

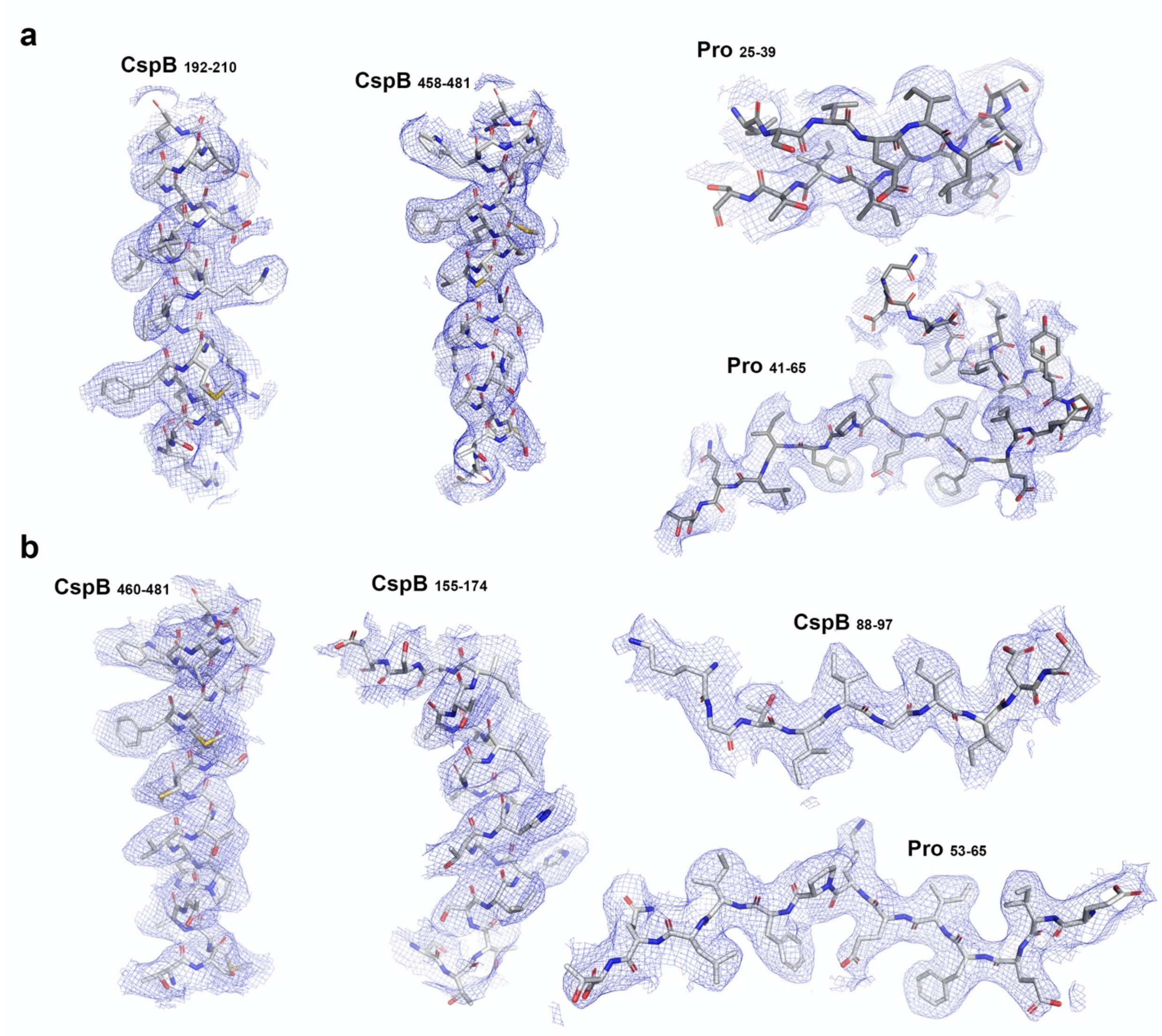

Supplementary Fig. 14| Structural features of the pro/CspB structures resolved by cryo-EM. Representative cryo-EM electron-density maps for selected regions of pro/CspB dimer (**a**) and (**b**) pro/CspB fibril, highlighting the quality of the reconstruction and the agreement between the density and the refined atomic model. Residue ranges corresponding to each displayed region are indicated above the maps. The cryo-EM density is shown as a blue mesh surrounding the refined model (at a threshold level of 3.5σ). Volume and structural representations were prepared using PyMol 3.

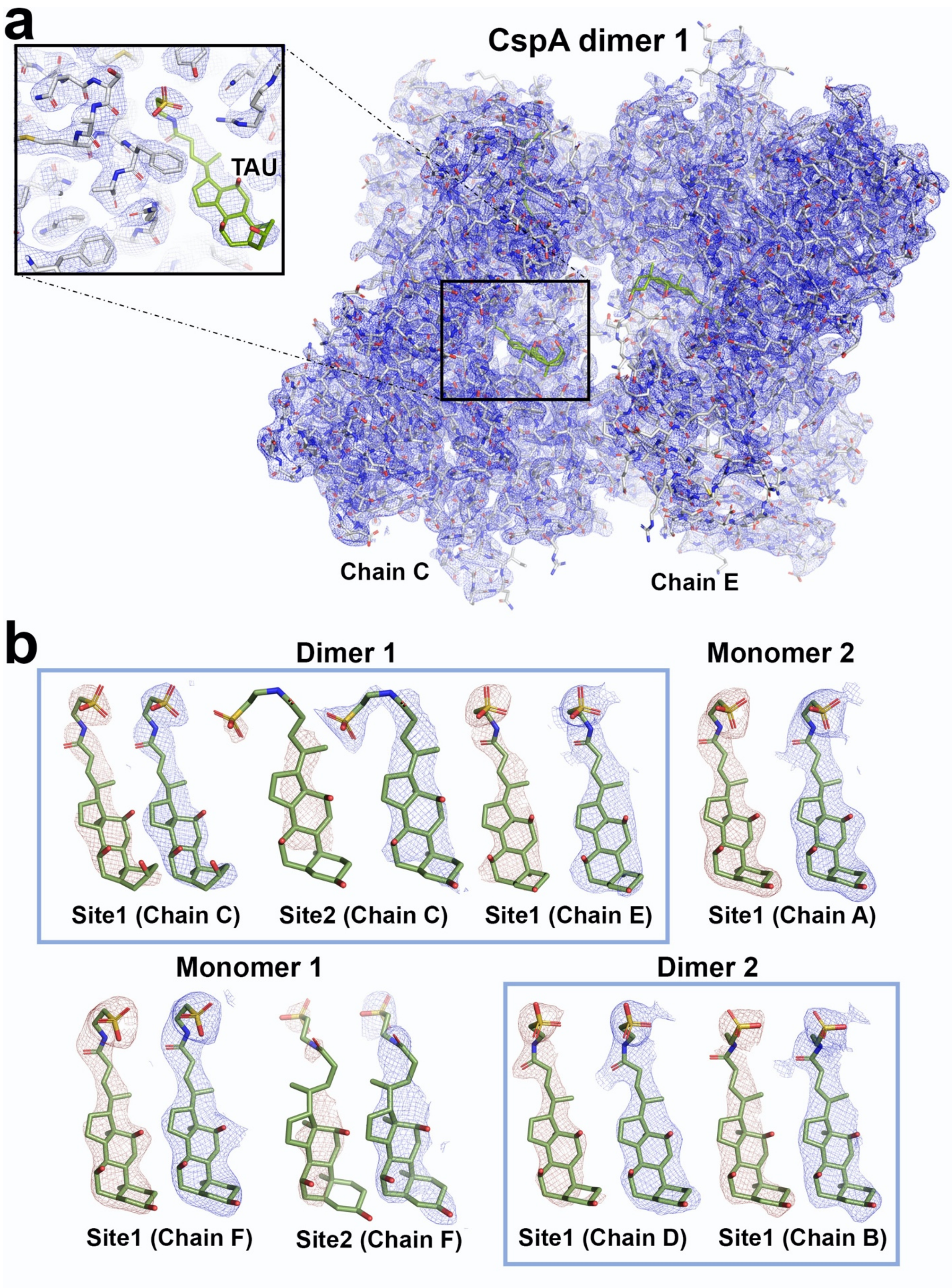
a
CspA dimer 1
TAU
Chain C
Chain E
b
Dimer 1
Monomer 2
Site1 (Chain C)
Site2 (Chain C)
Site1 (Chain E)
Site1 (Chain A)
Monomer 1
Dimer 2
Site1 (Chain F)
Site2 (Chain F)
Site1 (Chain D)
Site1 (Chain B)

Supplementary Fig. 15 | Overall view of the electron-density map for the CspA:taurocholate complex determined by X-ray crystallography at 2.9 Å resolution. a Representative electron-density map (2Fo-Fc map contoured at 1σ) for one of the two homodimers present in the asymmetric unit (dimer 1). Left, zoomed view of the recognition of TAU ligand (green sticks) at site 1. **b** Initial Fo-Fc difference maps contoured at 2.5σ (shown in brown) for all the taurocholate molecules in the asymmetric unit (represented in sticks and superimposed for comparison purposes). The associated final refined electron-density map (2Fo-Fc map contoured at 1σ) is also displayed as a blue mesh with the modeled ligand shown in sticks.

References

1. Rohlfing, A. E. *et al.* The CspC pseudoprotease regulates germination of Clostridioides difficile spores in response to multiple environmental signals. *PLOS Genet.* **15**, e1008224 (2019).

2. Adams, C. M., Eckenroth, B. E., Putnam, E. E., Doublié, S. & Shen, A. Structural and Functional Analysis of the CspB Protease Required for Clostridium Spore Germination. *PLoS Pathog.* **9**, e1003165 (2013).

3. Shen, A. *et al.* Mechanistic and structural insights into the proteolytic activation of Vibrio cholerae MARTX toxin. *Nat. Chem. Biol.* **5**, 469–478 (2009).

4. Shen, A. *et al.* Simplified, Enhanced Protein Purification Using an Inducible, Autoprocessing Enzyme Tag. *PLOS ONE* **4**, e8119 (2009).

5. Shrestha, R., Cochran, A. M. & Sorg, J. A. The requirement for co-germinants during Clostridium difficile spore germination is influenced by mutations in yabG and cspA. *PLOS Pathog.* **15**, e1007681 (2019).

6. Yamazawa, R. *et al.* Identification of the active site and characterization of a novel sporulation-specific cysteine protease YabG from Bacillus subtilis. *J. Biochem. (Tokyo)* **171**, 315–324 (2022).

7. Kochan, T. J. *et al.* Intestinal calcium and bile salts facilitate germination of Clostridium difficile spores. *PLOS Pathog.* **13**, e1006443 (2017).
