# Supplementary material for: Structure of core assembly of the Clostridioides difficile germinosome
Source: Nat Commun. 2026 Jun 17;17:7669. doi: 10.1038/s41467-026-74264-w (PMC13434707; doi:10.1038/s41467-026-74264-w)
Supplement: Supplementary file 3 [file 41467_2026_74264_MOESM3_ESM.pdf]

nature portfolio

Peer Review File

Structure of Core Assembly of the Clostridioides difficile Germinosome

Corresponding Author: Professor Shahriar Mobashery

This file contains all reviewer reports in order by version, followed by all author rebuttals in order by version.

Version 0:

Reviewer comments:

Reviewer #1

(Remarks to the Author)
Key findings: The paper maps the “core” of the C. difficile germinosome: CspA and CspB form homodimers; CspA forms an ultra-tight heterodimer with CspC (Surface Plasmon Resonance-SPR KD ≈ 97 ± 8 pM; Microscale thermophoresis-MST low-nM). CspA binds the germinant taurocholate (µM affinity), while neither CspB nor CspC binds taurocholate; glycine does not bind any Csp protein. High-resolution X-ray/cryo-EM structures are presented for pro/CspB (including a fibrillar supramolecular assembly), CspA bound to CHAPS, and the CspA:CspC:taurocholate complex, which together define three taurocholate sites (two on CspA; one at the CspA:CspC interface). Data are deposited (PDB/EMDB).

Major strengths of the paper: (1) Clear biophysical narrative with orthogonal methods. ITC/MST/SPR/AUC jointly argue that CspA (not CspC) recognizes taurocholate; the CspA:CspC interaction is exceptionally tight and seen by both immobilized (SPR) and solution (MST) formats. (2) High-quality structures and thoughtful model building. Crystal and cryo-EM maps are consistent (RMSD ~0.4 Å for CspB), and the supramolecular fibril is reconstructed to 2.75 Å with helical parameters justified from PyHI/RELION refinements. (3) Mechanistic specificity. The three taurocholate sites rationalize why homodimers cannot bind equivalently; the interface site emerges only in the heterodimer. (4) Data transparency. Structures/maps are deposited with accession codes (9ST7/9SJB/9SJ8/9SJ9; EMDB entries listed).

Concerns/limitations of the paper: (1) Physiological validation is limited. The central notion—CspA as the germinant receptor—relies entirely on purified-protein biophysics/structures. There are no spore-based germination assays (e.g., cspA point mutants at the identified sites, complementation, or dose–response germination with taurocholate/glycine) to connect binding to function in situ. Notably, only weak CspB protease activity was observed, and only in the presence of 1 mM $ZnCl_2$ showing that the physiological conditions are likely not fully captured in vitro.(2) Native-PAGE mixtures use 10 mM taurocholate, a concentration within/above the critical micelle concentration (CMC 3–13 mM). The enhanced CspA:CspC band in this regime could reflect micelle-driven dimer dissociation and rebinding rather than monomer-ligand thermodynamics relevant at sub-CMC levels; the authors themselves note SPR/MST are compromised by micelles. Physiological interpretation of the 10 mM effect is therefore uncertain. (3) Detergent and micelle confounders. The CspA structure includes CHAPS bound—chemically similar to bile salts—raising the possibility that detergent occupancy influences pocket geometry/allostery; the authors do discuss why CHAPS cannot occupy all taurocholate sites but show no structural cross-valdation. (4) Labeling/immobilization artifacts are acknowledged but impactful. KD varies from picomolar (SPR) to low-nanomolar (MST), and binding can be abolished by tag positioning or Cy5 placement (suggesting proximity to the interface). While the authors handle this carefully, the true in-solution KD absent labels/tags remains bracketed rather than pinned! (5) Stoichiometry and thermodynamics. ITC at 50 µM dimeric CspA yields KD ~9 µM but cannot robustly determine stoichiometry due to sensitivity limits and non-sigmoidal isotherms for weaker binding. A global thermodynamic model integrating ITC/MST/SPR to extract stoichiometry across sites is missing. (6) “No glycine receptor among Csps” remains a negative result. The conclusion that glycine binds “elsewhere” is reasonable but would be bolstered by targeted tests (e.g., screening other germinosome candidates, or demonstrating glycine-induced conformational changes in the heterodimer even without direct binding). As written, it exposes a gap without narrowing candidates. (7) Fibril relevance is speculative. The striking CspB fibrils appear at high protein concentration during grid prep (~18 mg/mL) with no direct evidence they form in spores or are required for germination. The authors are appropriately cautious, but the Discussion leans on environmental plausibility rather than data.

Specific methodological suggestions: (1) Genetics & germination assays. Introduce alanine or conservative substitutions at residues forming Sites 1–3 (on CspA and the CspA:CspC interface), then quantify spore germination with taurocholate ± glycine (OD600 drop, DPA release, SleC processing). Rescue with WT alleles to confirm specificity. (2) Orthogonal binding under native-like conditions. Re-measure taurocholate binding and CspA:CspC formation using nanodiscs/liposomes and sub-CMC bile salt ranges, avoiding CHAPS. Compare to current ITC/SPR/MST to assess detergent/micelle effects. (3) If going for more detail on glycine binding, map glycine's entry point. Systematically test other germinosome/receptor candidates by SPR/MST (e.g., Ger family homologs or spore coat proteins) and apply chemical crosslinking-MS with glycine present; in parallel, unbiased genetic screens for glycine non-responders would directly identify the co-germinant receptor. (4) In-spore structural evidence. Use cryo-ET or subtomogram averaging on dormant spores to ask whether CspB fibrils exist and where CspA:CspC localizes upon taurocholate exposure. (6) Unified thermodynamic model. Perform multi-site ITC with competitive ligands (e.g., deoxycholate/taurodeoxycholate) and global fitting to extract stoichiometries/ΔH/ΔS per site; complement with native MS or equilibrium titrations to define ligand occupancy across sites 1–3. (7) Assay robustness. Repeat native-PAGE complex-formation experiments at ≤1 mM taurocholate (below CMC), and confirm assembly by Size Exclusion Chromatography -Multi Angle Light Scattering or AUC without dyes/tags.

Reviewer #2

(Remarks to the Author)
The article by Luna et. al presents structural and biophysical data on the C. difficile germinosome components. Specifically, the authors report the crystal structure of CspB (prior structure of CspB was C. perfringens CspB and there are minor differences between the 2). The authors also report the structure of a CspA homodimer and a cryoEM structure of the CspA/CspC heterodimer. Using a combination of ITC and MST, the authors find that CspA binds to CHAPS (a fortuitous finding during purification) and to taurocholate. The authors report that they could not detect binding of any of the proteins to glycine. The manuscript focuses on an unresolved aspect of C. difficile spore germination and the authors' data sheds light on some of the missing data.

Major comments:
1) The manuscript is overly combative in many areas. The authors must soften their language. Moreover, the authors make definitive statements in many areas, but other interpretations are available. For example, the authors state, flatly, that the Csp proteins do not bind glycine. However, there are many reasons why the authors could not DETECT binding (below) and their overly combative stance is not necessary. Or the statement (Lines 177 – 179) that pro/CspB does not interact with CspA and CspC… under the conditions tested needs to be added.

2) Figure 1g. The ITC experiments could be measuring the displacement of CHAPS by taurocholate. ITC or MST may not be able to detect the binding of glycine. Other amino acids are able to act as co-germinants for C. difficile spores (Shrestha & Sorg 2018) and could be better used in the experiments herein.

Alternatively, and very importantly, the authors seem to have ignored the data found in Ramirez & Abel-Santos 2010 that suggests cooperative binding between TA and glycine. The authors did not test this but still make the statement that these are not the receptors for glycine. It's very possible that binding of TA influences the binding of glycine. Finally, the data by Kochan & Hanna indicates are role for calcium during germination. It is unknown how calcium functions, but if it involves germinant binding, the author would miss this.

3) Much of the discussion / manuscript ignores prior data in the field. Moreover, none of the data in this paper is backed up by studies in the organism. Given the ease with which mutations can now be made in C. difficile, this should be done. Otherwise, it is unknown if the authors' data translates to biological relevance.

4) Figure 1. The authors have positioned the Csp proteins at the outer spore membrane. There is no data to support this. Baloh & Sorg showed that the Csp proteins are present within the cortex layer (by immunoEM) and not restricted to the outer spore membrane.

Minor comments:
1) Lines 196 – 201. Taurocholate micelles are unlikely to be involved in germination of C. difficile spores. Total bile acid concentration in the upper ileum is ~10 mM (not just taurocholate). Moreover, in the colon during recurring bouts of C. difficile infection, spores germinate to cause disease. Taurocholate concentrations in the lower GI are below the CMC. Moreover, EC50 values for taurocholate are below the CMC (Abel-Santo and Sorg labs).

2) Hard to parse out what proteins are what in Figure 2e. An immunoblot would be far better representation of the data.

3) Lines 269 – 272. This data was also shown in the Adams and Shen manuscript.

4) Line 340. Biologically relevant is not tested here and this is supposition only.

5) There is similar data posted to BioRxiv by McNellis & Shen where they describe CspC / CspA heterodimer and CspA homodimer. This should be cited as the data is already available for public viewing and review. Their article tests the implications of their findings on C. difficile spore germination, as is standard for the field.

Reviewer #3

(Remarks to the Author)
In their manuscript, Cal y Mayor Luna and Alcorlo Pages et al. look into the molecular biology of spore germination in the important human pathogen C. difficile. The use biophysical, biochemical and structural studies to investigate a cascade of complex formation and proteolytic processing steps that result in the activation of the lytic transglycosylase SleC, which degrades peptidoglycan in the spore cortex.

The germination mechanism of C. difficile spores is a timely and medically important topic. The authors provide new insights into mutually exclusive binary complexes from the protein CspA, CspB and CspC, and how this is thought to chronologically result in SleC activation. The experimental work is of very high quality and well presented, as are the experimental procedures.

In some areas, the manuscript and the presented conclusions could still benefit from additional clarification, as detailed below.

- The model presented in Fig. 1B needs further clarification. In the germinosome cascade, what is the function of the CspC-CspA complex? From the main text it would seem the authors argue that formation of the CspC-CspA complex titrates CspA out from proCspB-CspA complexes, thus liberating proCspB and activation its proteolytic activity. The current data and presentation raises a number of questions.

(a) Fig1B shows a CspC-CspA complex under Sporulation conditions (left), and a taurocholate-bound CspC-CspA complex during germination (right). How does the function of these complexes differ?

(b) According to the schematic, it would appear proCspB is already liberated during Sporulation. What stops it from dimerising and degrading proSleC at this stage?

(c) The schematic drawing in Fig. 1B now serves both as introduction and as a summary of their findings and proposed mode of action. It would be advisable to split these two aspects, and thus include a new figure with the mechanistic model of the germination cascade.

- Using different techniques, the authors show that CspA forms high affinity dimers. Are these thought to occur in vivo, or would these be an artefact from the in vitro studies? If occurring in vivo, how do the CspA dimers fit into the mechanistic model of the germination cascade? It would appear that this goes against the hypothesis that taurocholate would titrate out CspA from proCspB-CspA complexes, liberating the proCspB protease.

- Main text lines 144-162. The authors show a lack of binding response in the titration of the CspC-CspA complex with taurocholate, and conclude that glycine must be binding another receptor of unknown identity. Did the authors check for glycine binding to CspC-CspA in presence of taurocholate, so as to rule out a coincidence binding mechanism?

- Ln 561-563. The authors suggest that taurocholate induces the formation of the CspA-CspC complex. This seems a plausible hypothesis, but what is the experimental evidence it does so? In absence of such evidence, the claim should be written less affirmatively.

- Can the authors speculate on the biological relevance and role of the proCspB oligomers? Ideally, the biochemical and biophysical findings would be evaluated in a biological context, to see if these result in germination artefacts.

Version 2:

Reviewer comments:

Reviewer #1

(Remarks to the Author)
The revised manuscript by Luna et al. presents a detailed biophysical and structural analysis of the germinosome components involved in Clostridioides difficile spore germination. The authors have made a number of changes in response to the reviewers' comments, and several aspects of the revision improve the clarity and robustness of the study.

First, the authors have removed the potentially confusing CHAPS-bound structure and replaced it with a crystal structure of the CspA–taurocholate complex, which strengthens the structural basis of the proposed germinant-recognition mechanism. This change substantially clarifies the interpretation of the ligand-binding data and avoids the possibility that detergent

binding might have influenced pocket geometry or allosteric behavior. The new structure therefore directly supports the central claim that CspA participates in taurocholate recognition.

Second, the authors have addressed an important mechanistic gap regarding glycine recognition. In the revised manuscript they report additional SPR experiments demonstrating that glycine binds the CspA:CspC:taurocholate complex, but not the individual proteins or the heterodimer alone. This result strengthens the proposed model of co-germinant sensing and resolves one of the key conceptual concerns raised in the original review.

Third, the authors have clarified the mechanistic model of germination by replacing the previous schematic with a new model figure (now Fig. 6). The revised model more clearly separates established structural findings from the inferred sequence of events involving YabG processing, formation of the CspA:CspC complex, ligand binding, and downstream activation of CspB leading to SleC processing. This revision improves the logical flow of the manuscript and responds appropriately to earlier concerns about the clarity of the model.

Despite these improvements, several of the central limitations noted in the initial review remain only partially addressed(minor comments for revision);

-The principal concern continues to be the absence of physiological validation in spores. The central conclusion—that CspA functions as the germinant receptor—still relies almost entirely on purified protein biophysics and structural data. While these experiments are technically strong and internally consistent, the manuscript would be considerably strengthened by demonstrating that mutations at the identified taurocholate-binding sites affect germination in vivo. The authors argue that such genetic analyses are beyond the scope of the current work, but this remains an important gap when linking ligand binding to biological function and wording in the manuscropt should reflect such needed caution.

-Similarly, the functional significance of the CspB fibrillar assemblies remains speculative. Although the cryo-EM structure of the fibril is intriguing, the manuscript still does not provide evidence that such assemblies occur in spores or contribute to germination. The revised discussion appropriately frames this observation more cautiously, but the biological relevance of this structure remains unclear.

-Finally, some methodological issues discussed in the original review—such as the interpretation of binding affinities obtained with different biophysical techniques and the limitations in determining stoichiometry for taurocholate binding—are acknowledged but not fundamentally resolved. While the use of multiple orthogonal methods increases confidence in the interaction network, a unified thermodynamic description of ligand binding across the different sites would still be desirable.

Overall, the revisions improve the clarity of the structural and biochemical narrative and address several specific concerns raised during review, particularly regarding ligand binding and the mechanistic model. As indicted the manuscript still lacks direct physiological validation connecting the proposed molecular mechanism to germination in spores. The study nevertheless represents a substantial contribution to understanding the structural basis of germinant sensing in C. difficile, and the remaining limitations primarily relate to the scope of the work rather than to the quality of the data presented. I can see that based on the proposed data others can work on in vivo validation.

Reviewer #2

(Remarks to the Author)
The authors have responded to my prior comments and now provide new data regarding the binding of the CspA:C:TA complex with glycine. Moreover, the newly added crystal structure is a strength. However, I do disagree with their response that genetic study is beyond the scope of the work. This would bring the data to biological relevance.

Reviewer #3

(Remarks to the Author)
The authors responded to all comments and suggestions, and made changes to the manuscript accordingly. These further increase the clarity and impact of the manuscript. Particularly the new finding on the cooperative binding of taurocholate and glycine to the CspA:CspC complex bring substantial additional insights.

The germination of bacterial spores, which reside in long-term metabolic dormancy, is a remarkable and fascinating molecular process. This study brings important new insights in the cascade leading up to germination, and will be of great interest to the field.

Open Access This Peer Review File is licensed under a Creative Commons Attribution 4.0 International License, which permits use, sharing, adaptation, distribution and reproduction in any medium or format, as long as you give appropriate credit to the original author(s) and the source, provide a link to the Creative Commons license, and indicate if changes were made.
In cases where reviewers are anonymous, credit should be given to 'Anonymous Referee' and the source.
The images or other third party material in this Peer Review File are included in the article's Creative Commons license, unless indicated otherwise in a credit line to the material. If material is not included in the article's Creative Commons license and your intended use is not permitted by statutory regulation or exceeds the permitted use, you will need to obtain permission directly from the copyright holder.
To view a copy of this license, visit https://creativecommons.org/licenses/by/4.0/

I reproduce the comments of the three reviewers below verbatim in blue text and in quotation marks. My replies follow them immediately in black text.

Binding of CHAPS (a component of the buffer) to CspA was adventitious. It has no bearing on the findings of the manuscript nor to the biology of the system. *All experiments with taurocholate were/are done in the absence of CHAPS after we discovered that it (a buffer constituent) binds to CspA*. To avoid any confusion, we have removed the structure of CspA:CHAPS from the manuscript and replaced it with that of CspA:taurocholate complex at 2.9 Å resolution (Fig. 4, Supplementary Figs. 9 and 11). This structure was determined while our manuscript was under review. There is now no mention of CHAPS in the manuscript on revision in the interest of clarity.

Reviewer #1:

"Key findings: The paper maps the "core" of the *C. difficile* germinosome: CspA and CspB form homodimers; CspA forms an ultra-tight heterodimer with CspC (Surface Plasmon Resonance-SPR KD ≈ 97 ± 8 pM; Microscale thermophoresis-MST low-nM). CspA binds the germinant taurocholate (μM affinity), while neither CspB nor CspC binds taurocholate; glycine does not bind any Csp protein. High-resolution X-ray/cryo-EM structures are presented for pro/CspB (including a fibrillar supramolecular assembly), CspA bound to CHAPS, and the CspA:CspC:taurocholate complex, which together define three taurocholate sites (two on CspA; one at the CspA:CspC interface). Data are deposited (PDB/EMDB).": Thanks. This is a good summary.

"Major strengths of the paper: (1) Clear biophysical narrative with orthogonal methods. ITC/MST/SPR/AUC jointly argue that CspA (not CspC) recognizes taurocholate; the CspA:CspC interaction is exceptionally tight and seen by both immobilized (SPR) and solution (MST) formats. (2) High-quality structures and thoughtful model building. Crystal and cryo-EM maps are consistent (RMSD ~0.4 Å for CspB), and the supramolecular fibril is reconstructed to 2.75 Å with helical parameters justified from PyHI/RELION refinements. (3) Mechanistic specificity. The three taurocholate sites rationalize why homodimers cannot bind equivalently; the interface site emerges only in the heterodimer. (4) Data transparency. Structures/maps are deposited with accession codes (9ST7/9SJB/9SJ8/9SJ9; EMDB entries listed).": Thanks again.

The paragraph below contains considerable information. I will answer them individually within the paragraph:

"Concerns/limitations of the paper: (1) Physiological validation is limited. The central notion—CspA as the germinant receptor—relies entirely on purified-protein biophysics/structures. There are no spore-based germination assays (e.g., *cspA* point mutants at the identified sites, complementation, or dose–response germination with taurocholate/glycine) to connect binding to function *in situ*. Notably, only weak CspB protease activity was observed, and only in the presence of 1 mM $ZnCl_2$ showing that the physiological conditions are likely not fully captured *in vitro*.": I acknowledge this observation; I will comment more fully below. "(2) Native-PAGE mixtures use 10 mM taurocholate, a concentration within/above the critical micelle concentration (CMC 3–13 mM). The enhanced CspA:CspC band in this regime could reflect micelle-driven dimer dissociation and rebinding rather than monomer-ligand thermodynamics relevant at sub-CMC levels; the authors themselves note SPR/MST are compromised by micelles. Physiological interpretation of the 10 mM effect is therefore uncertain.": The formation of CspA:CspC heterocomplex increases in a concentration-dependent manner for taurocholate, for which we used 0, 0.1, 0.5, 2, and 10 mM taurocholate. The effect reaches the maximum at 10 mM, but it is seen at lower concentrations too. We are cognizant that the concentration of taurocholate increases up to 5 mM in the gut after antibiotic treatment (communication with Prof. Casey Theriot) and that concentrations of the bile salts in the human gut range from 2 to 30 mM (Darkoh *et al*., 2013). "(3) Detergent and micelle confounders. The CspA structure includes CHAPS bound—chemically similar to bile salts—raising the possibility that detergent occupancy influences pocket geometry/allostery; the authors do discuss why CHAPS cannot occupy all taurocholate sites but show no structural cross-valdation.": The CHAPS finding was adventitious. It has no bearing on the findings of the manuscript. *All experiments with taurocholate were/are done in the absence of CHAPS*. To avoid any confusion, we have removed the structure of CspA:CHAPS from the manuscript and replaced it with that of CspA:taurocholate complex at 2.9 Å resolution (Fig. 4, Supplementary Figs. 9 and 11). This structure was determined while our manuscript was under review. There

is now no mention of CHAPS in the manuscript in the interest of clarity. "(4) Labeling/immobilization artifacts are acknowledged but impactful. KD varies from picomolar (SPR) to low-nanomolar (MST), and binding can be abolished by tag positioning or Cy5 placement (suggesting proximity to the interface). While the authors handle this carefully, the true in-solution KD absent labels/tags remains bracketed rather than pinned!": Every tool has a limitation. I am grateful to Reviewer's own comment above that we have used "orthogonal methods…ITC/MST/SPR/AUC", which were all used because of the aforementioned limitations. However, even for the case of "picomolar (SPR) to low-nanomolar (MST)" that Reviewer kindly mentions here, the difference is a mere 7.3-fold. In kinetics, a difference of 10-fold corresponds to 1.4 kcal/mol of binding energy, which is not considered large (that for 7.3-fold difference is merely ~1.0 kcal/mol). So, the differences that disparate methods produce for the same binding event, while real within the standard deviations that we report, are not significant from a kinetics perspective. "(5) Stoichiometry and thermodynamics. ITC at 50 μM dimeric CspA yields KD ~9 μM but cannot robustly determine stoichiometry due to sensitivity limits and non-sigmoidal isotherms for weaker binding. A global thermodynamic model integrating ITC/MST/SPR to extract stoichiometry across sites is missing.": I appreciate the reviewer's comment, which brings me back to the limitations of each method. In the case of SPR with the given example, the sensorgrams did not permit determination of the microscopic rate constants, hence only steady-state analyses were possible. We had/have discussed this matter in the manuscript. The K_d obtained by MST is also determined under equilibrium conditions and, therefore, cannot provide microscopic kinetic information. Consequently, assessment of a global thermodynamic model is not feasible. If it were, we would have done it. Nonetheless, all three orthogonal methods, based on distinct detection principles, yielded consistent and robust *experimental* (not simulated) values for the kinetics. These are the key biologically relevant parameters for understanding the germination process. (6) "No glycine receptor among Csps" remains a negative result. The conclusion that glycine binds "elsewhere" is reasonable but would be bolstered by targeted tests (e.g., screening other germinosome candidates, or demonstrating glycine-induced conformational changes in the heterodimer even without direct binding). As written, it exposes a gap without narrowing candidates.": We documented that glycine does not bind to any of the Csp proteins individually or to the heterodimer of CspA:CspC (all reported in the original submission). But as assessed during revision, the receptor of glycine is the complex of CspA:CspC:taurocholate (additional text in the revised draft: page 13, the last two lines to the top eight lines on page 15. And, page 41, lines 9-14. I added one sentence to the Abstract too). "(7) Fibril relevance is speculative. The striking CspB fibrils appear at high protein concentration during grid prep (~18 mg/mL) with no direct evidence they form in spores or are required for germination. The authors are appropriately cautious, but the Discussion leans on environmental plausibility rather than data.": Yes, I agree that the supramolecular structure of CspB is striking. And, yes, it is a small speculation. I hope that any top journal such as *Nature Communications* would allow reporting of an unexpected/unusual discovery (a long helical supramolecular structure comprised of six homodimers of CspB per turn of a right-handed helix) as a minor part of the larger work.

"Specific methodological suggestions: (1) Genetics & germination assays. Introduce alanine or conservative substitutions at residues forming Sites 1–3 (on CspA and the CspA:CspC interface), then quantify spore germination with taurocholate ± glycine (OD600 drop, DPA release, SleC processing). Rescue with WT alleles to confirm specificity.": The mutagenesis part is relatively uncomplicated, but to draw meaningful conclusions at the protein/organismal level is not. The Shen group (Rohlfing *et al.*, 2019) documented that genetic phenotypes alone can be difficult to interpret, noting that CspC mutants displayed complex responses that could reflect direct or indirect binding/interaction of bile salts, amino acids or ion recognition. We agree with assessment of the Shen group. Furthermore, the present detailed work is a biophysical/biochemical/structural biology undertaking that has taken three groups with six lab workers four years to accomplish (the main text now stands at 54 pages). We respectfully feel that the genetic study that is requested is beyond the scope of the present work. "(2) Orthogonal binding under native-like conditions. Re-measure taurocholate binding and CspA:CspC formation using nanodiscs/liposomes and sub-CMC bile salt ranges, avoiding CHAPS. Compare to current ITC/SPR/MST to assess detergent/micelle effects.": Per comment earlier in this reply to the critiques, CHAPS was not used in any subsequent experiments. The experiments were performed at the concentration of taurocholate much lower than CMC (up to 1 mM for ITC and SPR and 250 μM for MST; mentioned in the Methods section). Neither CspA nor CspC is a membrane protein to suit the use of nanodiscs/liposomes. It is important to underscore that the structure of the CspA:CspC complex was solved in the absence of detergent.

"(3) If going for more detail on glycine binding, map glycine's entry point. Systematically test other germinosome/receptor candidates by SPR/MST (e.g., Ger family homologs or spore coat proteins) and apply chemical crosslinking-MS with glycine present; in parallel, unbiased genetic screens for glycine non-responders would directly identify the co-germinant receptor.": I addressed this issue in several previous lines. As discussed in the revised draft, the receptor of glycine is the complex of CspA:CspC:taurocholate (additional text in the revised draft: page 13, the last two lines to the top eight lines on page 15. And, page 41, lines 9-14. I added one sentence to the Abstract too). "(4) In-spore structural evidence. Use cryo-ET or subtomogram averaging on dormant spores to ask whether CspB fibrils exist and where CspA:CspC localizes upon taurocholate exposure.": My structural biology colleagues estimate that this study will take them two years to complete. I regret that it is outside the scope of the present manuscript. "(6) Unified thermodynamic model. Perform multi-site ITC with competitive ligands (e.g., deoxycholate/taurodeoxycholate) and global fitting to extract stoichiometries/ΔH/ΔS per site; complement with native MS or equilibrium titrations to define ligand occupancy across sites 1–3. These experiments require producing the CspA:CspC heterocomplex at high concentrations, which would introduce multiple equilibria between the CspA homodimer and the CspA:CspC complex at the concentrations that are needed (~30–50 µM) for multi-site ITC. In addition, the ITC experiments with CspA and taurocholate that we have already reported in the manuscript produced non-sigmoidal isotherms, precluding reliable determination of stoichiometry by kinetics, which incidentally our X-ray structures already revealed. Together, these limitations would likely result in inconclusive ITC data rather than a meaningful multi-site thermodynamic model. "(7) Assay robustness. Repeat native-PAGE complex-formation experiments at ≤1 mM taurocholate (below CMC), and confirm assembly by Size Exclusion Chromatography - Multi Angle Light Scattering or AUC without dyes/tags.": As indicated above, the formation of CspA:CspC heterocomplex increases progressively in the presence of 0.1, 0.5, 2 and 10 mM taurocholate. We know that the concentration of taurocholate increases up to 5 mM in the gut after antibiotic treatment (communication with Prof. Casey Theriot) with concentrations of bile salts in the human gut ranging from 2 to 30 mM. The effect reaches the maximum at 10 mM, but it is seen at lower concentrations too. In our microscopy experiments, our collaborators have never observed micelle formation at the taurocholate concentration used, notwithstanding the CMC.

<u>Reviewer #2</u>:

"The article by Luna et. al presents structural and biophysical data on the *C. difficile* germinosome components. Specifically, the authors report the crystal structure of CspB (prior structure of CspB was C. perfringens CspB and there are minor differences between the 2). The authors also report the structure of a CspA homodimer and a cryoEM structure of the CspA/CspC heterodimer. Using a combination of ITC and MST, the authors find that CspA binds to CHAPS (a fortuitous finding during purification) and to taurocholate. The authors report that they could not detect binding of any of the proteins to glycine. The manuscript focuses on an unresolved aspect of C. difficile spore germination and the authors' data sheds light on some of the missing data.": Thanks for the good summary.

"Major comments:
1) The manuscript is overly combative in many areas. The authors must soften their language. Moreover, the authors make definitive statements in many areas, but other interpretations are available. For example, the authors state, flatly, that the Csp proteins do not bind glycine. However, there are many reasons why the authors could not DETECT binding (below) and their overly combative stance is not necessary. Or the statement (Lines 177 – 179) that pro/CspB does not interact with CspA and CspC… under the conditions tested needs to be added.": It did not occur to me that I was being combative. We just interpreted our results. If a binding is not seen, I would be compelled to say so. But, sincerely, I was happy to add comments like "under the conditions tested" in the revised draft.

"2) Figure 1g. The ITC experiments could be measuring the displacement of CHAPS by taurocholate. ITC or MST may not be able to detect the binding of glycine. Other amino acids are able to act as co-germinants for C. difficile spores (Shrestha & Sorg 2018) and could be better used in the experiments herein.": *All* experiments had/have been done in the absence of CHAPS, once we noted its binding to CspA. We have taken out any reference to CHAPS on revision, so as to avoid confusion. Shrestha & Sorg (2018), cited by the

Reviewer, showed that glycine is by far the most potent co-germinant. Notwithstanding the absence of binding of glycine to any of the Csp proteins individually or to the heterodimer of CspA:CspC (reported first in the original submission), we report on revision that the complex of CspA:CspC:taurocholate is the actual glycine receptor (discussed in more detail below).

"Alternatively, and very importantly, the authors seem to have ignored the data found in Ramirez & Abel-Santos 2010 that suggests cooperative binding between TA and glycine. The authors did not test this but still make the statement that these are not the receptors for glycine. It's very possible that binding of TA influences the binding of glycine. Finally, the data by Kochan & Hanna indicates are role for calcium during germination. It is unknown how calcium functions, but if it involves germinant binding, the author would miss this.": I appreciate the reviewer's comment regarding the potential cooperativity in binding. This was an insightful comment. We addressed this possibility by performing an additional SPR experiment. The heterocomplex CspC:CspA was captured on a CM5 SPR chip. The chip-bound heterocomplex was saturated with taurocholate to give the CspC:CspA:taurocholate complex. Glycine was then injected as an analyte at various concentrations. We observed binding of glycine to the CspC:CspA:taurocholate complex (K_d = 11 ± 6 nM). We have added appropriate text to the revised draft (page 13, the last two lines to the top eight lines on page 15. And, page 41, lines 9-14. I added one sentence to the Abstract too). On the other hand, the role of calcium in spore germination remains unclear. The Sorg lab (Francis *et al*., 2015) demonstrated that spore cortex hydrolysis precedes calcium dipicolinate release from the spore core, indicating that endogenous calcium is not involved in germinant binding. Moreover, the Kochan & Hanna study used concentrations of exogenous calcium far above physiological levels, making it difficult to derive conclusions about calcium's role in germination.

"3) Much of the discussion / manuscript ignores prior data in the field. Moreover, none of the data in this paper is backed up by studies in the organism. Given the ease with which mutations can now be made in C. difficile, this should be done. Otherwise, it is unknown if the authors' data translates to biological relevance.": The mutagenesis part is relatively uncomplicated, but to draw meaningful conclusions at the protein/organismal level is not. The Shen group (Rohlfing *et al*., 2019) documented that genetic phenotypes alone can be difficult to interpret, noting that CspC mutants displayed complex responses that could reflect direct or indirect binding/interaction of bile salts, amino acids or ion recognition. We agree with assessment of the Shen group. The present detailed work is a biophysical/biochemical/structural biology undertaking that has taken three groups with six lab workers four years to accomplish (the main text now stands at 54 pages). We respectfully feel that the genetic study that is requested is beyond the scope of the present work.

In general, the question is one of interpretation of the phenotypic observations, which can mislead. I sincerely hope that the Reviewer agrees with me that all the carefully executed biophysical measurements with homogenously purified proteins, X-ray and cryoEM structures reported in our manuscript should not be trumped by the absence of mutational studies.

"4) Figure 1. The authors have positioned the Csp proteins at the outer spore membrane. There is no data to support this. Baloh & Sorg showed that the Csp proteins are present within the cortex layer (by immunoEM) and not restricted to the outer spore membrane.": The Csp proteins were not being depicted in the outer spore membrane. We have modified the figure by adding a bit of space between the outer membrane and the proteins to avoid this misinterpretation. I thank the Reviewer for this comment. In response to the request of Reviewer 3, we moved the figure to the end of the manuscript (now as Figure 6).

"Minor comments:
1) Lines 196 – 201. Taurocholate micelles are unlikely to be involved in germination of C. difficile spores. Total bile acid concentration in the upper ileum is ~10 mM (not just taurocholate). Moreover, in the colon during recurring bouts of C. difficile infection, spores germinate to cause disease. Taurocholate concentrations in the lower GI are below the CMC. Moreover, EC50 values for taurocholate are below the CMC (Abel-Santo and Sorg labs).": The formation of CspA:CspC heterocomplex increases in the presence of 0.1, 0.5, 2 and 10 mM taurocholate. The maximal effect was seen at 10 mM. We know that the concentration of taurocholate increases up to 5 mM in the gut after antibiotic treatment (communication with Prof. Casey Theriot) with

concentrations of bile salts in the human gut ranging from 2 to 30 mM. The effect is seen at lower concentrations. Notwithstanding, the experiments were performed at the concentration of taurocholate much lower than CMC (up to 1 mM for ITC and SPR and 250 µM for MST; mentioned in the Methods section). In our microscopy experiments, our collaborators have never observed micelle formation at the taurocholate concentrations that were used.

“2) Hard to parse out what proteins are what in Figure 2e. An immunoblot would be far better representation of the data.”: This is a native gel involving merely three homogeneously purified proteins. I ask for the indulgence of the reviewer on this matter.

“3) Lines 269 – 272. This data was also shown in the Adams and Shen manuscript.”: Yes, indeed this sentence was included just after mentioning the structural similarity between Pro/CspB of *C. perfringens* (the Shen work) and of *C. difficile* (ours) To avoid, any confusion, we have added “Also, as observed in the *C. perfringens* case the catalytic domain of pro/CspB (residues 68–256 and 395–537) is interrupted by a ~137-amino-acid insertion that folds into a β-barrel jelly-roll domain, structurally integrated into the protease core” (page 15 of the revised draft, line 16).

“4) Line 340. Biologically relevant is not tested here and this is supposition only.”: We merely had/have written of the dimeric structure that it “is likely to be biologically relevant” (page 19, line 6 from the bottom). As described in the manuscript, the observed dimeric structure of pro/CspB (by both X-ray crystallography and cryo-EM experiments) is robust due to the many interactions between the monomers. This finding is in agreement with our biochemical and biophysical characterizations.

“5) There is similar data posted to BioRxiv by McNellis & Shen where they describe CspC / CspA heterodimer and CspA homodimer. This should be cited as the data is already available for public viewing and review. Their article tests the implications of their findings on C. difficile spore germination, as is standard for the field.”: The stated article is a BioRxiv posting, which was entirely independent of our work. This posting appeared while our manuscript was being reviewed. We have cited the actual paper on revision, which appeared in the literature in January (citation 32 on page 32, line 7 from the bottom).

<u>Reviewer #3</u>:

“In their manuscript, Cal y Mayor Luna and Alcorlo Pages et al. look into the molecular biology of spore germination in the important human pathogen C. difficile. The use biophysical, biochemical and structural studies to investigate a cascade of complex formation and proteolytic processing steps that result in the activation of the lytic transglycosylase SleC, which degrades peptidoglycan in the spore cortex.

The germination mechanism of C. difficile spores is a timely and medically important topic. The authors provide new insights into mutually exclusive binary complexes from the protein CspA, CspB and CspC, and how this is thought to chronologically result in SleC activation. The experimental work is of very high quality and well presented, as are the experimental procedures.”: I am grateful for the summary and the laudatory remarks.

“In some areas, the manuscript and the presented conclusions could still benefit from additional clarification, as detailed below.”: I have made an effort to address all these comments on revision.

“- The model presented in Fig. 1B needs further clarification. In the germinosome cascade, what is the function of the CspC-CspA complex? From the main text it would seem the authors argue that formation of the CspC-CspA complex titrates CspA out from proCspB-CspA complexes, thus liberating proCspB and activation its proteolytic activity. The current data and presentation raises a number of questions.

“(a) Fig1B shows a CspC-CspA complex under Sporulation conditions (left), and a taurocholate-bound CspC-CspA complex during germination (right). How does the function of these complexes differ?

(b) According to the schematic, it would appear proCspB is already liberated during Sporulation. What stops it from dimerising and degrading proSleC at this stage?

(c) The schematic drawing in Fig. 1B now serves both as introduction and as a summary of their findings and proposed mode of action. It would be advisable to split these two aspects, and thus include a new figure with the mechanistic model of the germination cascade.": Questions a, b, and c highlight mechanistic gaps in the spore-germination pathway, which the current knowledge cannot fully address. We agree that the previous Fig. 1b combined our findings and the earlier status of knowledge into one figure. We removed the old Fig. 1b (original submission) and replaced it with the new Fig. 6. I would like to clarify that proCspBA is produced as a fusion protein. As shown in the new Fig. 6, YabG proteolyzes it to produce proCspB and CspA. On formation of CspA, it binds to CspC (the picomolar union), followed by the assembly of the three taurocholates and glycine, which signals the onset of germination. As proCspB is formed during the YabG reaction, it undergoes an autolysis of its prodomain, which remains associated with the protein, leading to dimerization and oligomerization of the protein. The process that releases the prodomain from CspB is not known presently, but might be a local conformational change. This description is given in the Introduction of the manuscript, but also in the legend for the new Fig. 6 (page 33).

"- Using different techniques, the authors show that CspA forms high affinity dimers. Are these thought to occur in vivo, or would these be an artefact from the in vitro studies? If occurring in vivo, how do the CspA dimers fit into the mechanistic model of the germination cascade? It would appear that this goes against the hypothesis that taurocholate would titrate out CspA from proCspB-CspA complexes, liberating the proCspB protease.": The crystal structure of the CspA:taurocholate complex, now included in the revised manuscript, nicely provides insights (page 22, last three lines to the end of page 23; Figure 4; Supplementary Figs. 9 and 11). Among the six independent CspA chains present in the asymmetric unit of the crystal, four are arranged as homodimers and two as monomers, thus providing structural information on how both the dimeric and monomeric CspA exist. A variable number of taurocholate molecules were observed among the CspA dimers and monomers. One dimer binds three and the other two taurocholates, and for the monomers, one has two and the other just one taurocholate molecule. Most of taurocholates (six of eight) occupied Site 1, while the remaining two occupy Site 2; in all cases, taurocholate shows fewer interactions with the protein than in the CspA:CspC heterodimer. It is important to note that none exists in Site 3, which requires the presence of CspC in the heterodimer. In summary, while CspA can bind taurocholates both as monomer and homodimer, only the CspA:CspC heterodimer is capable of full taurocholate binding at three molecules. Based on our experimental data, and on the published work from the Shen lab (Kevorkian *et al*., 2016) showing that the presence of CspA is required for CspC incorporation into *C. difficile* spores, we conclude that the biologically relevant species is the CspA:CspC heterodimer rather than the CspA monomer/homodimer. The formation of CspA (from hydrolysis of proCspBA) enabled complexation with CspC to form the heterodimer, which in turn serves as the receptor for binding of taurocholate and glycine, the germinant and co-germinant.

"- Main text lines 144-162. The authors show a lack of binding response in the titration of the CspC-CspA complex with taurocholate, and conclude that glycine must be binding another receptor of unknown identity. Did the authors check for glycine binding to CspC-CspA in presence of taurocholate, so as to rule out a coincidence binding mechanism?": This is of course an insightful comment. This experiment was done on revision of the manuscript. We observed binding of glycine to the CspC:CspA:taurocholate complex (K_d = 11 ± 6 nM). The CspC:CspA:taurocholate complex is the receptor for glycine. We have added appropriate text to the revised draft (page 13, the last two lines to the top eight lines on page 15. And, page 41, lines 9-14. I added one sentence to the Abstract too). I am grateful for the suggestion by Reviewer 3.

"- Ln 561-563. The authors suggest that taurocholate induces the formation of the CspA-CspC complex. This seems a plausible hypothesis, but what is the experimental evidence it does so? In absence of such evidence, the claim should be written less affirmatively.": The evidence comes from concentration-dependence for taurocholate in formation of the CspA:CspC complex, our biophysical measurements, the native-PAGE assay and the X-ray structure for CspA monomer and homodimer bound to taurocholate and the structure of CspA:CspC:taurocholate complex.

"- Can the authors speculate on the biological relevance and role of the proCspB oligomers? Ideally, the biochemical and biophysical findings would be evaluated in a biological context, to see if these result in germination artefacts.": We observed that the protein was capable of forming oligomeric assemblies of variable length. These oligomers displayed a defined and extended architecture, characterized by a linear arrangement without detectable curvature, suggesting a relatively rigid conformation. The formation of such rigid and elongated assemblies may reflect an intrinsic propensity of the protein to establish ordered intermolecular contacts in environments with high macromolecular crowding and partial dehydration. These physicochemical conditions mimic those within the spore core. We propose that the formation of CspB fibrils may function to concentrate or immobilize CspB within the spore environment. Oligomerization could serve to stabilize the protease under extreme environmental conditions or regulate its activity by sequestering or scaffolding it in an inactive state until germination signaling promotes its activation. A previous report documented another protease capable of forming an oligomeric structure. The case is that of the serine peptidase Vpr forming fibrils outside bacilli (Cheng *et al*., 2023).

References:

Darkoh, C., Brown, E. L., Kaplan, H. B., & DuPont, H. L. (2013). Bile Salt Inhibition of Host Cell Damage by Clostridium Difficile Toxins. PLOS ONE, 8(11), e79631. https://doi.org/10.1371/journal.pone.0079631

Cheng, Y., Han, J., Song, M., Zhang, S., & Cao, Q. (2023). Serine peptidase Vpr forms enzymatically active fibrils outside Bacillus bacteria revealed by cryo-EM. *Nature Communications*, *14*(1), 7503. https://doi.org/10.1038/s41467-023-43359-z

Francis, M. B., Allen, C. A., & Sorg, J. A. (2015). Spore Cortex Hydrolysis Precedes Dipicolinic Acid Release during Clostridium difficile Spore Germination. Journal of Bacteriology, 197(14), 2276-2283. https://doi.org/10.1128/JB.02575-14

Kevorkian, Y., Shirley, D. J., & Shen, A. (2016). Regulation of Clostridium difficile spore germination by the CspA pseudoprotease domain. Biochimie, 122, 243-254. https://doi.org/10.1016/j.biochi.2015.07.023

Rohlfing, A. E., Eckenroth, B. E., Forster, E. R., Kevorkian, Y., Donnelly, M. L., Benito De La Puebla, H., Doublié, S., & Shen, A. (2019). The CspC pseudoprotease regulates germination of Clostridioides difficile spores in response to multiple environmental signals. PLOS Genetics, 15(7), e1008224. https://doi.org/10.1371/journal.pgen.1008224

The reviewers' comments are reproduced verbatim in blue text and in quotation marks. My replies follow them in black text.

Reviewer #1:

"The revised manuscript by Luna et al. presents a detailed biophysical and structural analysis of the germinosome components involved in Clostridioides difficile spore germination. The authors have made a number of changes in response to the reviewers' comments, and several aspects of the revision improve the clarity and robustness of the study.": I thank the Reviewer for the positive evaluation of the changes in the manuscript.

"First, the authors have removed the potentially confusing CHAPS-bound structure and replaced it with a crystal structure of the CspA–taurocholate complex, which strengthens the structural basis of the proposed germinant-recognition mechanism. This change substantially clarifies the interpretation of the ligand-binding data and avoids the possibility that detergent binding might have influenced pocket geometry or allosteric behavior. The new structure therefore directly supports the central claim that CspA participates in taurocholate recognition.

Second, the authors have addressed an important mechanistic gap regarding glycine recognition. In the revised manuscript they report additional SPR experiments demonstrating that glycine binds the CspA:CspC:taurocholate complex, but not the individual proteins or the heterodimer alone. This result strengthens the proposed model of co-germinant sensing and resolves one of the key conceptual concerns raised in the original review.

Third, the authors have clarified the mechanistic model of germination by replacing the previous schematic with a new model figure (now Fig. 6). The revised model more clearly separates established structural findings from the inferred sequence of events involving YabG processing, formation of the CspA:CspC complex, ligand binding, and downstream activation of CspB leading to SleC processing. This revision improves the logical flow of the manuscript and responds appropriately to earlier concerns about the clarity of the model.": I appreciate this summary of the new data included in the revised work.

"Despite these improvements, several of the central limitations noted in the initial review remain only partially addressed(minor comments for revision);": I note that Reviewer #1 does not have major comments for revision. I appreciate these minor comments, which I address below.

"-The principal concern continues to be the absence of physiological validation in spores. The central conclusion—that CspA functions as the germinant receptor—still relies almost entirely on purified protein biophysics and structural data. While these experiments are technically strong and internally consistent, the manuscript would be considerably strengthened by demonstrating that mutations at the identified taurocholate-binding sites affect germination in vivo. The authors argue that such genetic analyses are beyond the scope of the current work, but this remains an important gap when linking ligand binding to biological function and wording in the manuscropt should reflect such needed caution.": I appreciate this point on the need for wording and I have introduced suitable text to address it, per reviewer's request. Kindly see page 30, lines 16-18 and page 32, last four lines of the revised draft.

"-Similarly, the functional significance of the CspB fibrillar assemblies remains speculative. Although the cryo-EM structure of the fibril is intriguing, the manuscript still does not provide evidence that such assemblies occur in spores or contribute to germination. The revised discussion appropriately frames this observation more cautiously, but the biological relevance of this structure remains unclear.": We find the fibrillar structure very intriguing. It likely is not a chance occurrence and we agree that the biological relevance must be further explored. In any case our reporting of the fibrillar structure, does not affect the main message of the manuscript and provides new information that we trust will be of general interest for the readership of the journal and the scientific community.

“-Finally, some methodological issues discussed in the original review—such as the interpretation of binding affinities obtained with different biophysical techniques and the limitations in determining stoichiometry for taurocholate binding—are acknowledged but not fundamentally resolved. While the use of multiple orthogonal methods increases confidence in the interaction network, a unified thermodynamic description of ligand binding across the different sites would still be desirable.”: I agree entirely that it is desirable. However, for a system that does not lend itself to the “unified thermodynamics description” because of its complexity, the next best thing is to do the analysis by multiple orthogonal methods. That is what we have done. If the unified thermodynamics description could be done, I assure the reviewer that we would have been delighted to do it.

“Overall, the revisions improve the clarity of the structural and biochemical narrative and address several specific concerns raised during review, particularly regarding ligand binding and the mechanistic model. As indicted the manuscript still lacks direct physiological validation connecting the proposed molecular mechanism to germination in spores. The study nevertheless represents a substantial contribution to understanding the structural basis of germinant sensing in C. difficile, and the remaining limitations primarily relate to the scope of the work rather than to the quality of the data presented. I can see that based on the proposed data others can work on in vivo validation.”: I am very grateful to the reviewer for this thoughtful summation.

Reviewer #2:

“The authors have responded to my prior comments and now provide new data regarding the binding of the CspA:C:TA complex with glycine. Moreover, the newly added crystal structure is a strength. However, I do disagree with their response that genetic study is beyond the scope of the work. This would bring the data to biological relevance.”: I thank reviewer #2. While the genetic study would nicely complement our biochemical and structural work, I regret that I am not able to perform the mutagenesis experiments at this point for two reasons. I hope that the reviewer appreciates my situation. (1) First, my lab does mutagenesis in association with determination of the structures of the variant proteins to back up the mechanistic claims that we would make with structures. I am uncomfortable doing these experiments any other way, as it might leave us open to a need for a correction at a later date (possibly by others). Furthermore, this will be a major undertaking for us due to the multivalent nature of taurocholate recognition in all the three taurocholate-binding sites and the nature of the interface of the CspA-CspC complex. (2) We lack presently the personnel to commit to these experiments, which will take us several months to complete once the suitable staff is in place. I sincerely hope that the reviewer would relate to my difficulty.

Per suggestion of Reviewer #1, I added some text to the revised manuscript addressing this issue (page 30, lines 16-18 and page 32, last four lines). We recognize in the revised draft that the biological relevance of the models that we propose should be explored by mutational analysis in *C. difficile* in support of the model that we have proposed in the manuscript.

Reviewer #3:

“The authors responded to all comments and suggestions, and made changes to the manuscript accordingly. These further increase the clarity and impact of the manuscript. Particularly the new finding on the cooperative binding of taurocholate and glycine to the CspA:CspC complex bring substantial additional insights.

The germination of bacterial spores, which reside in long-term metabolic dormancy, is a remarkable and fascinating molecular process. This study brings important new insights in the cascade leading up to germination, and will be of great interest to the field. ”: I am indebted to Reviewer #3 for the thoughtful and laudatory comments.
